# Oral anticoagulants: a systematic overview of reviews on efficacy and safety, genotyping, self-monitoring, and stakeholder experiences

**DOI:** 10.1186/s13643-022-02098-w

**Published:** 2022-10-28

**Authors:** Claire Khouja, Ginny Brunton, Michelle Richardson, Gillian Stokes, Laurence Blanchard, Helen Burchett, Meena Khatwa, Ruth Walker, Kath Wright, Amanda Sowden, James Thomas

**Affiliations:** 1 Centre for Reviews and Dissemination, University of York, York, UK; 2 EPPI-Centre, UCL Institute of Education, London, UK; 3 London School of Hygiene and Tropical Medicine, London, UK

**Keywords:** Atrial fibrillation, Oral anticoagulants, Overview, Qualitative reviews, Systematic review, Venous thromboembolism

## Abstract

**Background:**

This systematic overview was commissioned by England’s Department of Health and Social Care (DHSC) to assess the evidence on direct (previously ‘novel’) oral anticoagulants (OACs), compared with usual care, in adults, to prevent stroke related to atrial fibrillation (AF), and to prevent and treat venous thromboembolism (VTE). Specifically, to assess efficacy and safety, genotyping, self-monitoring, and patient and clinician experiences of OACs.

**Methods:**

We searched MEDLINE, Embase, ASSIA, and CINAHL, in October, 2017, updated in November 2021. We included systematic reviews, published from 2014, in English, assessing OACs, in adults. We rated review quality using AMSTAR2 or the JBI checklist. Two reviewers extracted and synthesised the main findings from the included reviews.

**Results:**

We included 49 systematic reviews; one evaluated efficacy, safety, and cost-effectiveness, 17 assessed genotyping, 23 self-monitoring or adherence, and 15 experiences (seven assessed two topics). Generally, the direct OACs, particularly apixaban (5 mg twice daily), were more effective and safer than warfarin in preventing AF-related stroke. For VTE, there was little evidence of differences in efficacy between direct OACs and low-molecular-weight heparin (prevention), warfarin (treatment), and warfarin or aspirin (secondary prevention). The evidence suggested that some direct OACs may reduce the risk of bleeding, compared with warfarin. One review of genotype-guided warfarin dosing assessed AF patients; no significant differences in stroke prevention were reported. Education about OACs, in patients with AF, could improve adherence. Pharmacist management of coagulation may be better than primary care management. Patients were more adherent to direct OACs than warfarin. Drug efficacy was highly valued by patients and most clinicians, followed by safety. No other factors consistently affected patients’ choice of anticoagulant and adherence to treatment. Patients were more satisfied with direct OACs than warfarin.

**Conclusions:**

For stroke prevention in AF, direct OACs seem to be more effective and safer than usual care, and apixaban (5 mg twice daily) had the best profile. For VTE, there was no strong evidence that direct OACs were better than usual care. Education and pharmacist management could improve coagulation control. Both clinicians and patients rated efficacy and safety as the most important factors in managing AF and VTE.

**Systematic review registration:**

PROSPERO CRD42017084263—one deviation; efficacy and safety were from one review.

**Supplementary Information:**

The online version contains supplementary material available at 10.1186/s13643-022-02098-w.

## Background

Oral anticoagulants (OACs) are routinely used in the UK to prevent stroke in atrial fibrillation (AF) and to prevent or treat venous thromboembolism (VTE). The 2014 NICE guidance [1, 2] recommended the use of both warfarin (a vitamin K antagonist) and direct (or direct-acting or non-vitamin K antagonist, previously referred to as novel) OACs; dabigatran, rivaroxaban, apixaban, and edoxaban. This guidance was updated between 2018 and 2021 [3–5]. With exceptions for specific conditions, the new guidance recommends any of the four direct OACs for stroke prevention in AF; for VTE treatment, apixaban and rivaroxaban are recommended; for secondary prevention, continued treatment or apixaban are recommended; and for primary prevention, various options are recommended depending on the reason for hospitalisation.

Clinicians and patients still have choices about which recommended OAC to prescribe or take. Although warfarin was standardly used before the approval of direct OACs, it requires regular patient monitoring to maintain coagulation in the target range [1, 6]. Patients are also required to avoid foods containing vitamin K, and to have a low alcohol intake. Common side effects are bleeding (leading to bruising, nosebleeds, and headaches), mild rash and hair loss [7], and warfarin is known to interact with other medications [7]. These factors can lead to poor adherence. Direct OACs only require annual rather than daily to quarterly blood tests [8], and there are fewer indications of interactions with other medications [9, 10]. They are more expensive than warfarin, but if they are more effective due to better adherence, they could be more cost-effective.

This systematic review of systematic reviews (overview from this point on) was commissioned, in 2018, by the Department of Health and Social Care (DHSC) in England with the aim of identifying, assessing, and summarising the relevant research evidence that had been published since the 2014 NICE guidance. Since our full report was completed in 2018, new evidence has become available, filling some of the gaps that we had identified. Although the NICE guidance has been updated, this overview incorporates new research and is a useful guide for practitioners. It compares direct OACs with warfarin, in adults, to prevent stroke in AF, or to prevent or treat VTE. We focussed on efficacy and safety, self-monitoring, genotype-guided dosing, and patient and clinician experiences of OACs. Given the large volume of research, an overview of existing systematic reviews was considered to be the most appropriate method [11, 12]. Here we provide a summary of the 2018 report [13], and of the systematic reviews published since that report.

### Research questions addressed

RQ1. What evidence syntheses have been conducted to address the efficacy of UK-approved OAC therapy with respect to:

- Warfarin versus direct OACs in different patient cohorts?
- The evidence for an optimised pathway on genotyping?
- The evidence for an optimised pathway on self-monitoring?

RQ2. What evidence syntheses have been conducted to address the safety of UK-approved OAC therapy with respect to:

- Renal function and the long-term use of direct OACs?
- Complications associated with warfarin and direct OACs, including bleeding and stroke risk?

RQ3. What are patient and clinician experiences of UK-approved OAC therapy concerning:

- The impact of direct OACs and warfarin on patient lifestyle?
- Medicines adherence and compliance of direct OACs and warfarin?
- Clinician perceptions of direct OACs and warfarin?
- Monitoring international normalised ratio (INR)s in patients receiving vitamin K antagonists and the effect on patient adherence?

## Methods

The protocol for this systematic overview was registered on PROSPERO (CRD42017084263). Academic, clinician, NHS, and DHSC stakeholders were consulted throughout the review. The overview is reported in accordance with the PRISMA statement, and the checklist is in Additional file 1. We initially searched MEDLINE, Embase, ASSIA, and CINAHL, in October, 2017, and we updated these searches on 30 November, 2021. Search terms covered oral anticoagulants, systematic review and their synonyms, and the medical condition (where appropriate). The searches were limited to articles published since 2014, when the previous NICE guidance on OACs was issued [1]. The original and updated MEDLINE strategies are in Additional files 2 and 3.

To be included, articles had to meet the following criteria:

- Published since 2014
- Available in English
- Focussed on OECD settings
- Focussed on adults eligible for oral anticoagulation
- Focussed on OACs for the prevention of stroke related to AF, or the acute treatment, or primary or secondary (after a VTE) prevention, of VTE
- Focussed on warfarin, dabigatran, rivaroxaban, edoxaban, or apixaban
- Be a systematic review, with a search of more than one database
- Assess therapeutic doses of warfarin, compared with a direct OAC, or a comparison between direct OACs
- Report health or cost outcomes or stakeholder experiences

At least two reviewers independently screened titles and abstracts, until agreement was over 90%, then single reviewers completed screening of abstracts and potentially relevant full texts. For the update search, we used priority screening [14] (a new feature of EPPI-Reviewer web) [15] to identify and prioritise those articles that were most likely, based on the screening results for the initial search, to meet the inclusion criteria. We stopped screening when we were including, for assessment on full text, fewer than one in fifty articles. Data were extracted, quality assessed and synthesised by single reviewers, and checked by and agreed with another reviewer. EPPI-Reviewer© software [15, 16] was used to manage the process.

We extracted pre-defined descriptive characteristics from the reviews, including.

- Year of publication;
- Date range of included primary studies;
- Setting (community, hospital, etc.);
- Main topic focus (efficacy, safety, experiences, or cost);
- Target population (health condition, or at-risk group);
- Participant characteristics (age, gender, etc.);
- Intervention characteristics (type of oral anticoagulant, or self-monitoring);
- Number, countries, and designs of primary studies included in the review;
- Type of outcomes measured (health outcomes, hospitalisation, health-related quality of life, stakeholder experiences, etc.); and
- Author and year of primary studies (to assess overlap between reviews).

We coded the review characteristics and assigned reviews to each research question or subsection. We narratively synthesised or summarised the data. For self-monitoring or adherence, the primary outcome was time in therapeutic range (TTR) or proportion of days covered (PDC). For genotyping, where the outcomes were not reported for patients with AF or VTE separately from patients with other conditions, we described the reviews, rather than extracting efficacy or safety data. Summary tables were produced (see Table 2, Table 3, Table 4, and Additional file 4).

The included systematic reviews were assessed for risk of bias using AMSTAR2 [17] or the JBI Critical Appraisal Checklist for Systematic Reviews and Research Syntheses [18], as appropriate. Where we included multiple reviews addressing the same question, overlap in primary studies was assessed [19]. Overlap means that the same primary studies are included in more than one review, giving their results more weight than others, which can bias the conclusions [11].

## Results

**Fig. 1:**
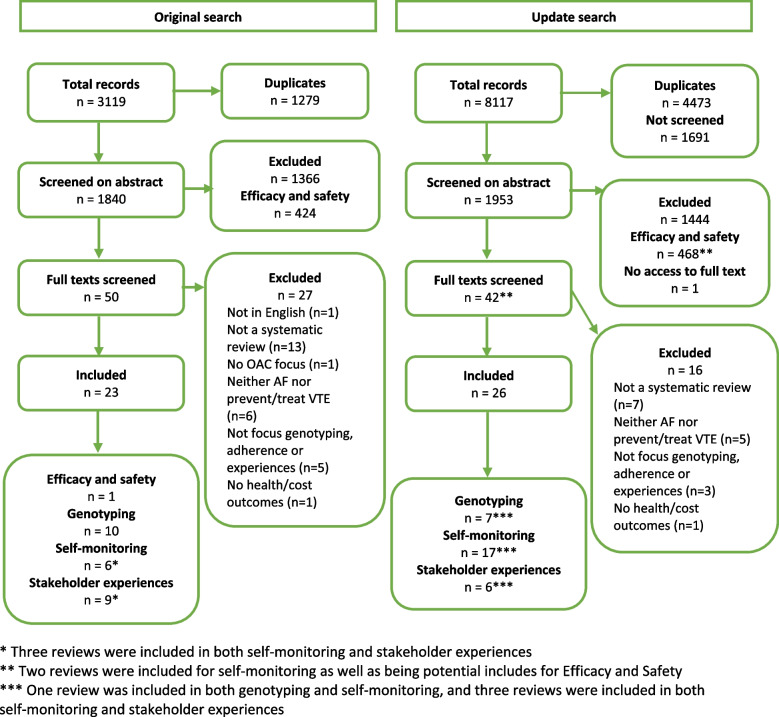
Flow diagram

Figure 1 shows the flow diagram for the articles included in this overview. The initial search identified 1840 unique articles, and the update identified an additional 3644 unique articles. After the initial search, we consulted with NHS commissioners and together decided to focus on one recent, rigorous, and comprehensive systematic review to address the two questions of effectiveness and safety (RQ1 and RQ2) [20]. This systematic review included network meta-analyses on the prevention of stroke in AF, and the primary and secondary prevention and treatment of VTE. The remaining 424 articles that were identified as possibly relevant, based on their titles and abstracts, are listed with the full report [13]. The update search and screening identified an additional 468 articles as possibly relevant, and these are listed in Additional file 5, with brief details of five overviews that were identified; four [21–24] on AF and one [25] on pulmonary embolism. Many of the additional reviews focussed on patients with specific conditions, such as renal disease, cancer, or diabetes, or undergoing various procedures, or elderly or obese patients. From the initial search, 50 articles were screened on full text and 23 of these were included. From the update search, we screened 1953 articles on title and abstract and 42 on full text, and included 26 additional reviews. In addition to the review on efficacy and safety, we included a total of 17 reviews for genotyping, 23 reviews for self-monitoring or adherence, and 15 reviews for stakeholder experiences (six reviews were relevant to both self-monitoring and stakeholder experiences, and one to both self-monitoring and genotyping). Based on full text, the most common reason for exclusion was not reporting systematic review methods; the reasons for exclusion are reported in Additional file 6. Table 1 summarises the findings for all the research questions.

**Table 1:** Summary of results for all research questions

Research question	Summary of findings	Risk of bias in the primary evidence^a^
RQ1 (a) Efficacy of warfarin versus direct OACs in different patient cohorts?	For AF, direct OACs were more effective and safer than usual care, and apixaban 5 mg twice daily had the best profile. For VTE, overall, direct OACs were no better than low-molecular-weight heparin (prevention in hip or knee surgery), warfarin (treatment), and warfarin or aspirin (secondary prevention)	Overall, low risk of bias. Some outcomes had low, high, or unclear risks Few direct comparisons
RQ2 (b) Complications associated with warfarin and direct OACs, including bleeding and stroke risk?		
RQ1 (b) The evidence for an optimised pathway on genotyping?	One review found no difference between genotype-guided warfarin dosing and direct OACs for stroke prevention in patients with AF. Systematic reviews of genotype-guided dosing for direct OACs, in patients with AF or VTE are needed	Very low to moderate quality (GRADE). The most common flaw was a lack of blinding
RQ1 (c) The evidence for an optimised pathway on self-monitoring?	In patients with AF, education with or without a decision aid improved time in therapeutic range, while self-monitoring and self-testing made little difference. Evidence was lacking for patients with VTE	Low-to-moderate or uncertain quality
RQ2 (a) Safety relating to renal function and the long-term use of direct OACs?	Several reviews were identified and are listed in Additional file 5	Not applicable
RQ3 (a) The impact of direct OACs and warfarin on patient lifestyle?	Patients were more satisfied with pharmacist management, and with direct OACs, than with usual care	The quality of the primary studies varied
RQ3 (b) Medicines adherence and compliance of direct OACs and warfarin?	For patients, it seems that knowledge, past experience, disease-related issues, and support needs influence OAC choices and adherence. Adherence was better with direct OACs than with warfarin	
RQ3 (c) Clinician perceptions of direct OACs and warfarin?	Efficacy was the main driver of the choice of OAC, followed by safety, except for geriatricians, where safety was more important than efficacy	
RQ3 (d) Monitoring INRs in patients receiving vitamin K antagonists and the effect on patient adherence?	Pharmacist management could improve time in therapeutic range, compared with usual primary care	Low-, uncertain-, and high-quality evidence

*RQ* research question, *AF* atrial fibrillation, *OAC* oral anticoagulant, *VTE* venous thromboembolism, *GRADE* Grading of Recommendations Assessment, Development and Evaluation

^a^As reported by the authors of the reviews included from the original search

### Efficacy and safety

The systematic review [20] on efficacy, safety, and cost-effectiveness was assessed as having low risk of bias in 11 of the 16 AMSTAR2 domains; full results are in Additional file 7. The summary characteristics are described in Table 2 below. As the four topics were distinct, there was no overlap in primary studies between the sections.

**Table 2:** Efficacy and safety review characteristics and main findings

Sterne et al. (2017)[20] review section	Methods and study details	Primary outcomes (number of studies) Main comparator	Main findings
Prevention of AF-related stroke	**Search:** March 2014, updated September 2014 **Included**: 23 RCTs on AF (41 articles); 94,656 participants **Published:** 1989 to 2014 **Quality tool**: Cochrane Risk of Bias	**Efficacy:** stroke or systemic embolism (15); ischaemic stroke (13); myocardial infarction (15) **Safety:** major bleeding (18); clinically relevant bleeding (12); intracranial bleeding (6); all-cause mortality (18) Main comparator: warfarin	The analyses suggested that direct OACs were better than warfarin for most efficacy and safety outcomes Apixaban (5 mg twice daily) was likely to be one of the best options for almost all outcomes.^* ^For example, all-cause mortality (OR 0.88, 95% CI 0.79 to 0.98; versus warfarin, INR 2 to 3); and expected incremental net benefit £7533 (95% CI 489.9 to 18,228; at a willingness-to-pay threshold of £20,000 per quality-adjusted life-year)
VTE primary prevention (mainly in hip and knee surgery)	**Search:** March 2014, updated September 2014 **Included**: 43 RCTs on VTE (46 articles); 77,563 participants **Published:** 1996 to 2012 **Quality tool**: Cochrane Risk of Bias	**Efficacy:** symptomatic VTE (29);^a^ symptomatic DVT (25); symptomatic PE (35) **Safety:** myocardial infarction (9); major bleeding (39); clinically relevant bleeding (27); all-cause mortality (28) **Main comparator:** low-molecular-weight heparin	There was no strong evidence to support direct OACs; no direct comparisons with warfarin and few events Warfarin (INR 2 to 3) was likely to be best (*p* > 0.9)^* ^for major bleeding (OR 0.57, 95% CI 0.39 to 0.82), and low-molecular-weight heparin for clinically relevant bleeding (*p* > 0.6) Rivaroxaban was most likely to be cost-effective, but very uncertain; incremental net monetary benefit (INMB), total replacement of hip £453, 95% CI − 485 to 1312; knee £16, 95% CI − 406 to 329; £20,000 threshold
Acute treatment of VTE	**Search:** March 2014, updated September 2014 **Included**: 9 RCTs on VTE (10 articles); 28,803 participants **Published:** 2007 to 2014 **Quality tool**: Cochrane Risk of Bias	**Efficacy:** symptomatic VTE (8);^b^ symptomatic DVT (9); symptomatic PE (9); myocardial infarction (5) **Safety:** major bleeding (9); clinically relevant bleeding (8); all-cause mortality (8) Main comparator: warfarin	Analyses suggested that direct OACs were no better than warfarin, but apixaban (5 mg twice daily, e.g., major bleeding OR 0.33, 95% CI 0.18 to 0.56) and rivaroxaban (15 mg twice daily then 20 mg once daily, e.g., major bleeding OR 0.55, 95% CI 0.37 to 0.80) may be better for avoiding bleeding Apixaban (5 mg twice daily) was likely to be one of the best for most outcomes (e.g., *p* > 0.9 for major bleeding; INMB £710, 95% CI − 1322 to 2185; £20,000 threshold)^*^
Secondary prevention of VTE	**Search**: March 2014, updated September 2014 **Included:** 10 RCTs on VTE (11 articles); 10,390 participants **Published**: 1999 to 2013 **Quality tool**: Cochrane Risk of Bias	**Efficacy:** symptomatic VTE (10); symptomatic DVT (9); symptomatic PE (9) **Safety:** myocardial infarction (5); major bleeding (10); clinically relevant bleeding (6); all-cause mortality (9) Main comparator: warfarin	Inconsistent evidence suggested; apixaban (2.5 mg twice daily) was worse than warfarin for symptomatic PE (OR 10.1, 95% CI 1.66 to 102), but better for avoiding bleeding (HR 0.24, 95% CI 0.09 to 0.61); dabigatran (150 mg twice daily) was also better for bleeding (HR 0.54, 95% CI 0.41 to 0.71) There were not enough data for the authors to calculate the likelihood of being the best option None of the treatments was cost-effective, except possibly aspirin (INMB £623, 95% CI − 6404 to 4602; £20,000 threshold)

^a^In the analyses, 28 trials were included for VTE, 20 for DVT, 30 for PE, nine for myocardial infarction, 34 for major bleeding, 25 for clinically relevant bleeding, and 24 for mortality

^b^Table 107 in Sterne’s report shows eight studies with this outcome, while the summary (p171) only mentions seven

^*^These probabilities of being best are from rankograms, where a higher probability indicates a higher likelihood of being the best option. See https://methods.cochrane.org/cmi/glossary for the definition of a rankogram

*AF* atrial fibrillation, *RCT* randomised controlled trial, *OR* odds ratio, *INR* international normalised ratio, *CI* confidence interval, *DVT* deep vein thrombosis, *PE* pulmonary embolism, *INMB* incremental net monetary benefit, *HR* hazard ratio

Randomised controlled trials (RCTs) were included in the systematic review [20] if they assessed adults who were eligible for OACs. Apixaban, edoxaban, rivaroxaban, dabigatran, and betrixaban (not licenced in the UK) were compared with warfarin, low-molecular-weight heparin, or antiplatelets (aspirin or clopidogrel). There were no direct comparisons (i.e., compared within the same trial) between direct OACs; they were evaluated using a common comparator across trials, usually warfarin, low-molecular-weight heparin, or placebo, in Bayesian fixed-effect network meta-analyses. The outcomes were stroke, symptomatic VTE, bleeding events, and death. Two of the 23 trials on patients with AF were conducted in the UK. Most of the included trials were rated by the review authors as at low risk of bias, except for the prevention of stroke in patients with AF, where the trials were rated as at a mix of low, high, and unclear risk. Most trials were relatively short (3 to 42 months, for stroke prevention in AF; 4 to 183 days, for VTE primary prevention; 12 to 48 weeks, for VTE treatment; and 3 to 52 months, for VTE secondary prevention) and funded by drug manufacturers.

For the prevention of stroke in patients with AF, direct OACs were generally more effective and safer than warfarin. Comparing direct OACs, apixaban (5 mg twice daily) had the best efficacy and safety profile and was most likely to be cost-effective. For patients at risk of VTE (undergoing hip or knee surgery or with a medical condition), there was no strong evidence that efficacy or safety differed between direct OACs and low-molecular-weight heparin. Rivaroxaban was most likely to be cost-effective, but with high uncertainty. For the treatment of VTE, there was little evidence that efficacy differed between direct OACs and warfarin, but apixaban (5 mg twice daily) and rivaroxaban (15 mg twice daily, then 20 mg once daily) could reduce the risk of (major and clinically relevant) bleeding, and apixaban (5 mg twice daily) was most likely to be cost-effective. For secondary prevention of VTE, there was little evidence that efficacy differed between direct OACs and warfarin, but apixaban (2.5 or 5 mg twice daily) and dabigatran (150 mg twice daily) could reduce the risk of bleeding. Aspirin was most likely to be cost-effective, but this was uncertain.

### Genotyping

From the initial search, none of the 10 reviews [26–35] on genotyping focussed exclusively on patients with AF or VTE. These reviews assessed patients receiving OACs for any condition, including cardiomyopathy, heart-valve replacement, and rheumatic heart disease, as well as AF or VTE (see Additional file 4 for details). The update searches identified seven reviews [36–42] that met the inclusion criteria (see Additional file 4 for details). One [38] of these reported data for patients with AF. This review met 12 and partly met one of the 16 relevant AMSTAR criteria and found no significant difference in efficacy of stroke prevention, between each direct OAC and genotype-guided warfarin dosing (the gene was not specified, but references indicated it was CYP2C9). The authors assessed the primary evidence as having moderate-to-high risk of bias.

All reviews assessed genotype-guided dosing for warfarin. We were unable to obtain the full text for one review that reported information on genotyping (ABCB1) for direct OACs, but without specifying the condition[43]. Overall, the reviews were rated as at moderate risk of bias. The AMSTAR2 risk of bias assessment for all reviews is in Additional file 7. Most of the 155 studies within the reviews (see Additional file 8) included patients with AF or VTE, but all reviews, except one [38], presented findings for patients with all conditions without subgroup analyses by AF or VTE. Therefore, we were unable to extract any results specifically for patients with AF or VTE from the remaining reviews. Overlap across the 17 reviews was slight (CCA: 4.5%); however, six studies were in 10 or 11 reviews.

### Self-monitoring

From the initial search, we identified six reviews [44–49] that addressed self-monitoring, and from the update search, a further 15 reviews [38, 50–63] that addressed self-monitoring or adherence. The summary characteristics are presented in Table 3, below. Quality assessment results are in Additional file 7.

**Table 3:** Self-monitoring review characteristics and main findings

Review authors (year), and topic focus	Methods and study details	Main findings for time in therapeutic range (TTR), proportion of days covered (PDC), or adherence
**Education, decision aids and self-management (*n* = 4)**		
Clarkesmith et al. (2017); [44] education; decision aids; self-management plus education	**Search:** update of 2013 review; February 2016 **Included**: 11 RCTs on AF (20 articles); 2246 adults **Published:** 1999 to 2014 **Quality tool:** Cochrane and GRADE **Primary outcome**: TTR, days in range and INR values in range	Low-quality evidence (six studies) suggests that education, with or without a decision aid or self-management, may improve values or time in range (e.g., mean TTR 69% SD 25.1 intervention, 64% SD 28.2% control; and self-management plus education MD 6.31%, 95% CI − 5.63 to 18.25)
Jang (2021) [55] Education, warfarin	**Search:** May 2020 **Included:** 12 studies, 4 RCTs and 1 other on AF, 1 RCT and 6 other on mixed conditions **Published:** 2014 to 2020 **Quality tool:** Downs and Black **Outcomes**: TTR, MMAS, knowledge, QoL, bleeding, mortality	All measures of knowledge were improved INR measures (TTR), mortality and readmission all improved with education
Song et al. (2021) [62] Decision aids DOAC and VKA	**Search:** January 2021 **Included:** 10 studies on AF **Published:** 1999 to 2018 **Quality tool:** Cochrane **Outcomes**: Adherence, knowledge, uptake, stroke and bleeding	Effects unclear for adherence (3 studies). Two studies found improved adherence with the decision aid at 3 months; one found no difference at 6 months
Torres Roldan (2021) [64] Decision aids DOAC and warfarin	**Search:** May 2020 **Included:** 2 RCTs, 4 other studies on AF **Published:** 2013 to 2020 **Quality tool:** Cochrane and NOS **Outcomes**: Adherence, knowledge, decision conflict, QoL	Adherence (MMAS and PQA) improved (two studies)
**Self-monitoring (*n* = 7)**		
Dhippayom et al. (2020) [54] Telemedicine warfarin	**Search:** September 2019 **Included:** 3 RCTs and 9 other studies, 11,478 patients, mixed conditions **Published:** 2005 to 2018 **Quality tool:** Cochrane EPOC **Outcomes**: TTR (undefined), INR in range, bleeding and thromboembolic events	For TTR (11 studies), self-testing with remote automated management was better than usual face-to-face care (MD 8.78%; 95% CI 0.06 to 17.50). Self-testing was the preferred option for TTR
Dhippayom et al. (2021) [53] Self-care warfarin	**Search:** May 2020 **Included:** 16 RCTs, 5859 patients, 2 AF and 14 mixed conditions **Published:** 2001 to 2020 **Quality tool:** Cochrane EPOC **Outcomes**: TTR, INR in range, bleeding, thromboembolic events, and mortality	For TTR (13 trials), more time was in range with weekly self-management (MD 7.67%, 95% CI 0.26 to 15.08), and weekly self-testing with remote management (MD 5.65%, 95% CI 0.04 to 11.26), compared with usual care
Heneghan et al. (2016); [46] self-testing or self-management	**Search:** update of 2010 review; July, 2015 **Included**: 28 RCTs (27 articles); two on AF, 20 mixed; 8950 participants **Published:** 1989 to 2012 **Quality tool:** GRADE **Primary outcome**: TTR, INR values in range	Low-quality evidence suggests no difference between self-testing and usual care for AF patients (one trial) Moderate-quality evidence for and against self-testing on time in range (three trials longer, four shorter TTR); and supporting self-testing on values in range (two trials, *p* < 0.05), in mixed populations Low-quality evidence that self-management improves time in range for AF patients (one trial *p* = 0.0061) Moderate-quality evidence for and against self-management on time in range (three trials longer, and three shorter TTR); and supporting self-management on values in range (eight trials), in mixed populations
Ng et al. (2020) [38] Self-care of warfarin vs DOACs	**Search:** November 2017 **Included:** 37 RCTs, 100,142 patients; 4 RCTs AF and 4 mixed for warfarin bundles **Published:** 2004 to 2014 **Quality tool:** Cochrane **Outcomes**: TTR, efficacy, stroke, bleeding and mortality	TTR was improved with warfarin care bundles (8 RCTs) that included genotype-guided dosing, self-management, self-testing and/or device implantation (mean 68.9%) compared with warfarin usual care (mean 61.1%)
Sharma et al. (2015); [48] self-testing or self-management	**Search:** update of 2007 Cochrane review;^a^ from 2007 to May 2013 **Included**: 26 RCTs (45 articles); two on AF, 18 mixed; 8763 participants **Published:** 1996 to 2012 **Quality tool:** Cochrane **Primary outcome**: TTR (% of time), INR values in range	Low-quality evidence suggests no difference between self-testing and usual care for AF patients (one trial), while self-testing may improve time and values in range in mixed populations (time; WMD 4.44%, 95% CI 1.71 to 7.18) Self-management improves time in range for AF patients (one trial), but in mixed populations, no effect on time in range (six trials), and conflicting evidence for values in range (five trials more values in range, two fewer)
Tran et al. (2021) [63] Telepharmacy warfarin	**Search:** November 2020 **Included:** 11 studies, 8,395 patients with mixed conditions **Published:** 2005 to 2018 **Quality tool:** Downs and Black **Outcomes**: TTR, thromboembolic events, bleeding, extreme INR, hospitalisation, mortality	No significant difference in TTR between in-person and remote pharmacist management (WMD − 0.02, 95% CI − 5.3 to 5.3; six studies, *n* = 957). Heterogeneity was moderate. Two studies did not use the Rosendaal [65] method
Xia et al. (2018) [61] Telemedicine (self-testing) warfarin	**Search:** July 2017 **Included:** 10 studies, 16,915 patients with mixed conditions **Published:** 2005 to 2017 **Quality tool:** NOS **Outcomes**: TTR, bleeding, thromboembolic events, hospital visits and admissions	TTR no significant differences between online and hospital management (OR − 0.55, 95% CI − 9.06 to 7.95; five studies, *n* = 2366). Heterogeneity was high
**Pharmacist management (*n* = 4)**		
Entezari-Maleki et al. (2016); [45] pharmacist-managed warfarin therapy	**Search:** January 2014 **Included**: 24 RCTs and non-RCTs on AF and VTE; 11,607 participants **Published:** 1995 to 2013 **Quality tool:** Downs and Black, and Jadad **Primary outcome**: TTR	Uncertain-quality evidence that pharmacist management may improve time in range (84.3% v 82.2%, 95% CI − 26.3 to 30.5, three trials; 72.1% v 56.7%, 95% CI 4.2 to 26.6, five observational studies)
Hou (2017) [66] Pharmacist management, warfarin	**Search:** April 2017 **Included:** 8 RCTs, 9 observational studies, 2 AF and VTE, 15 mixed conditions **Published:** 1998 to 2016 **Quality tool:** Cochrane, NOS and GRADE **Outcomes**: TTR, time in expanded range, bleeding, thrombosis, mortality, satisfaction, and costs	TTR (3 RCTs and 3 cohort studies) improved with pharmacist management (WMD: 8.03, 95% CI 2.19–13.88, *p* = 0.007); no significant difference for RCTs alone, nor for expanded range
Manzoor et al. (2017); [47] pharmacist-managed anticoagulation services	**Search:** May 2017 **Included**: 25 RCTs and non-RCTs; 23 on AF or VTE, two mixed; 12,252 adults **Published:** 1985 to 2016 **Quality tool:** Downs and Black **Primary outcome**: TTR, INR values in range, mean prothrombin	Uncertain-quality evidence that pharmacist management may improve time and values in range (23 out of 25 studies; improvement 1.7 to 28.0%; 19 statistically significant)
Zhou et al. (2016); [49] pharmacist-managed warfarin services	**Search:** July 2015 **Included**: eight RCTs on mixed conditions; 1493 adults **Published:** 2003 to 2013 **Quality tool:** Cochrane and GRADE **Primary outcome**: TTR	High-quality evidence that pharmacist management may improve time in range (MD 3.66, 95% CI 2.20 to 5.11; four trials), although this was not significant for time in extended therapeutic range (moderate-quality evidence)
**Adherence, discontinuation, switching, and persistence (*n* = 8)**		
Afzal et al. (2019) [50] Adherence DOACs	**Search:** November 2018 **Included:** 5 RCTs and 16 other studies; for adherence, 3 on AF, 1 on VTE and 1 on AF and VTE **Published:** 2013 to 2018 **Quality tool:** Cochrane and NOS **Outcomes**: MMAS-8, satisfaction, HRQoL, compliance, expectations	Adherence similar between DOACs and VKA (five studies). Higher adherence with more knowledge of OAC treatment (one study)
Buck et al. (2021) [51] Discontinuing DOAC or VKA	**Search:** 2019 **Included:** 12 studies on AF **Published:** 2014 to 2019 **Quality tool:** Gough’s dimension A **Outcomes**: Discontinuation	For VKA, at 1 year, discontinuation ranged from 6.8 to 17.3%, and for dabigatran was 36.8%. Similar rates VKA to dabigatran at 2 years. Discontinuation at 2 years ranged from 5.7 to 12% for warfarin, and 4.5 to 5.9% for other DOACs
Deitelzweig et al. (2021) [52] Persistence DOAC vs VKA	**Search:** July 2019 **Included:** 36 studies, on AF; 18 in the NMA, 395,593 patients **Published:** 2014 to 2019 **Quality tool:** ROBINS-I and GRADE **Outcomes**: Odds ratio on non-persistence at 30, 60 and 90 days	At 30 and 90 days all DOACs had lower odds of non-persistence than VKA. At 60 days, dabigatran had higher odds than, and apixaban and rivaroxaban were not significantly different to, VKAs. Over all measures, apixaban was most likely to have the lowest non-persistence (*p* = 95.7% at 30 days, *p* = 76.9% at 60 days and *p* = 98.4% at 90 days)
Ozaki et al. (2020) [56] Persistence DOACs	**Search:** June 2018 **Included:** 48 studies, 594,784 patients with AF **Published:** 2013 to 2018 **Quality tool:** NOS **Outcomes**: PDC, adherence (PDC ≥ 80%), and persistence	Mean PDC apixaban 81%, rivaroxaban 79%, dabigatran 72% (14 studies). Adherence was 71% (95% CI 64 to 78) for apixaban, 60% (95% CI 52 to 68) for dabigatran, and 70% (95% CI 64 to 75) for rivaroxaban (21 studies). Apixaban and rivaroxaban had higher persistence than VKA (OR 1.44, 95% CI 1.12 to 1.86; 24 studies)
Prentice et al. (2020) [57] Adherence to Rivaroxaban and Dabigatran	**Search:** August 2018 **Included:** 5 studies, 80,230 patients with AF **Published:** 2015 to 2017 **Quality tool:** GRACE checklist **Outcomes**: PDC ≥ 80%	Adherence higher with rivaroxaban than dabigatran (RR 1.08, 95% CI 1.03 to 1.12). PDC ≥ 80% for rivaroxaban ranged from 59.5 to 83.5%; for dabigatran ranged from 57.3 to 78.3%
Romoli et al. (2021) [58] Switching DOACs	**Search:** March 2020 **Included:** 5 studies, 259,308 patients with AF **Published:** 2017 to 2019 **Quality tool:** NOS **Outcomes**: risk of switching	Apixaban lower risk of switching than dabigatran (OR 0.29, 95% CI 0.25 to 0.34), and rivaroxaban (OR 0.58, 95% CI 0.50 to 0.67). Dabigatran higher risk than rivaroxaban (OR 2.35 95% CI 1.89 to 2.81)
Salmasi et al. (2020) [59] Adherence to DOACs and warfarin	**Search:** March 2019 **Included:** 30 studies, 593,683 patients with AF **Published:** 2001 to 2019 **Quality tool:** STROBE and ISPOR **Outcomes**: PDC ≥ 80%, MPR ≥ 80%, and compliance	Mean adherence (PDC ≥ 80%) at 1 year, for apixaban was 82% (95% CI 74 to 89), rivaroxaban 77% (95% CI 69 to 86), and dabigatran 75% (95% CI 68 to 82)
Shehab et al. (2019) [60] Adherence DOAC and VKA	**Search:** June 2016 **Included:** 6 studies, 1,640,157 patients (one study 1.5 million), on AF **Published:** 2015 to 2016 **Quality tool:** STROBE **Outcomes**: PDC > 80%, MMAS-8, and phone interview	Dabigatran 72.7% (95% CI 62.5 to 82.9), apixaban 59.9% (95% CI 32.0 to 123.1), rivaroxaban 59.3% (95% CI 38.7 to 80.0), heterogeneity was very high. VKA 29.5%

*TTR* time in therapeutic range, *INR* international normalised ratio, *AF* atrial fibrillation, *GRADE* Grading of Recommendations Assessment, Development and Evaluation, *SD* standard deviation, *MD* mean difference, *CI* confidence interval, *RCT* randomised controlled trial, *WMD* weighted mean difference, *VTE* venous thromboembolism, *DOAC* direct oral anticoagulant, *VKA* vitamin K antagonist, *p* probability, *RR* relative risk, *PDC* proportion of days covered, *OR* odds ratio, *MPR* medication possession ratio, *MMAS* Morisky Medication Adherence Scale, *NOS* Newcastle Ottawa Scale, *EPOC* Effective Practice and Organisation of Care, *GRACE* Good Research for Comparative Effectiveness, *STROBE* Strengthening the Reporting of Observational Studies in Epidemiology, *ISPOR* International Society of Pharmacoeconomics and Outcomes Research, *PQA* Pharmacy Quality Alliance

^a^Garcia-Alamino JM, Ward AM, Alonso-Coello P, Perera R, Bankhead C, Fitzmaurice D, et al. Self-monitoring and self-management of oral anticoagulation. Cochrane Database Syst Rev 2010;4:CD003839

Interventions to improve adherence included the following: education and/or decision aids (four reviews) [44, 55, 62, 64]; self-testing with clinician dosing or self-management with dosing according to rules (eight reviews) [38, 44, 46, 48, 53, 54, 61, 63]; and pharmacist management (four reviews) [45, 47, 49, 66]. The most common outcomes reported were TTR, proportion of INR measurements in range, or PDC (a measure used for direct OACs as they do not require as frequent blood monitoring as warfarin). Adherence [50, 57, 59, 60], persistence [52, 56], discontinuation [51], and switching [58] were also reported. Most reviews assessed patients with any condition (including AF or VTE); nine reviews focussed on patients with AF; one focussed on AF or VTE; none focussed on patients with VTE.

Overall, the reviews were rated as at low-to-moderate risk of bias (see Additional file 7), with few major flaws. One review [44] met 15 of 16 AMSTAR2 domains, one [48] fully or partly met 14, and one [54] fully or partly met 13. Five reviews met just under half of their relevant criteria [51, 57, 58, 61, 62], and five reviews met just over half of their relevant criteria [47, 50, 60, 63, 64]. The remaining reviews met most of their relevant criteria. Eight reviews mainly included RCTs; 15 reviews mainly included other study designs. The authors of six reviews rated primary-study quality as high or good [45, 49, 56, 57, 60, 61]. The authors of one review [47] reported scores ranging from 16 to 28 (mean 19.5; where 28 is the highest score) using the Downs and Black Checklist [67]. The remaining reviews reported a range of risks of bias (from low to high) or flaws in study design across included studies.

For patients with AF, low-quality evidence from a few studies in one review [44] suggested that education with or without a decision aid could improve control of coagulation, compared with usual care without education, at least in the short term (less than 1 year). One review [62] described the evidence on decision aids as unclear; in two studies, they improved adherence and in one they did not. Another review [64] found improved adherence, but the evidence was at high risk of bias. Compared with usual care, self-management appeared to improve coagulation, in most of the low-to-moderate-quality trials in three reviews [45, 47, 49]. The findings of a meta-analysis of two RCTs suggested that self-management plus education improved TTR, although the difference was not significant, compared with usual care without education (mean difference 6.31%, 95% confidence interval − 5.63 to 18.25) [44]. Together, four reviews [56–59] focussing on AF patients found higher PDC or less switching with apixaban than rivaroxaban, followed by dabigatran, then warfarin; one review [60] found that dabigatran had highest adherence, followed by apixaban, then rivaroxaban, and lastly vitamin K antagonists. One review [51] reported lower discontinuation at 2 years with direct OACs compared with VKA, but not at 1 year, and another review [52] reported the lowest likelihood of non-persistence with apixaban.

For patients requiring an OAC for a range of conditions (including AF and VTE), education improved adherence and TTR[55], as did decision aids[64]. Self-testing improved INR values in range, compared with usual care, but the low-to-moderate-quality evidence was contradictory for TTR, with both longer and shorter TTR reported across studies, within reviews[46, 48]. Three reviews reported improvements in TTR with self-testing over usual care [38, 53, 54]. Self-management was associated with both more and fewer values in range, and longer and shorter TTR [44, 46, 48], and TTR with remote management was not significantly different from with in-person management [61, 63]. A meta-analysis of four high-quality RCTs found a higher percentage of TTR with pharmacist management than with usual (primary) care (mean difference 3.66, 95% confidence interval 2.20 to 5.11; although this was not significant for time in extended therapeutic range) [49]. The findings of another meta-analysis were consistent [66]. Similarly, low- or uncertain-quality evidence suggested that pharmacist management could improve TTR [45, 47].

The extent of overlap (corrected covered area[19], CCA; see Additional file 8 for details) between studies included in the reviews was slight across four reviews for education or decision aids (4.9%), high across eight reviews for self-testing with or without self-management (13.7%); very high across four reviews for pharmacist management (21.2%); and moderate across eight reviews for adherence measures (7.0%).

### Stakeholder experiences

From the original search, we included nine reviews [44, 45, 49, 68–73] that focussed on the experiences of patients, and three of these also examined the experiences of physicians [68, 70, 72]. From the update, we included four reviews [50, 55, 74, 75] that focussed on patients, one [76] on clinicians, and one [51] was an analysis of reasons reported in medical records. The summary characteristics are presented in Table 4. Quality assessment results are in Additional file 9.

**Table 4:** Stakeholder experiences review characteristics and themes

Review authors (year), and topic focus	Methods and study details	Themes
Afzal et al. (2019); [50] patient-reported outcomes of DOAC vs warfarin	**Search:** November 2018 **Included:** 21 studies, AF and VTE, participants NR **Published**: 2013 to 2018 **Quality tool:** Cochrane and NOS	**Patients**: Equivalent health-related quality of life. Greater satisfaction with DOACs. Expectations, compliance and adherence were equivalent between DOACs and warfarin
Alamneh et al. (2016); [68] practices of anticoagulation in AF, uptake, impact, and persisting challenges	**Search**: NR **Included:** 140 observational studies, reviews, RCTs, experimental studies and guidelines; participants NR **Published**: 1991 to 2015 **Quality tool**: NR	**Patients: ** The lack of a specific reversal agent was a major concern (also for practitioners). Other major concerns were medication adherence and continuation of medication use, higher costs, and the lack of data for some groups of patients. A lesser concern was difficulty in remembering to take direct OACs without the requirement for regular monitoring **Practitioners: ** The uptake of direct OACs has been variable, with slow integration into clinical practice in most countries and limited impact on prescribing
Buck et al. (2021); [51] reasons for discontinuing DOAC or warfarin	**Search:** 2019 **Included:** 12 studies, AF, participants NR **Published**: 2014 to 2019 **Quality tool:** Gough’s weight of evidence	**Medical records:** Reasons for discontinuation – bleeding, gastrointestinal events, frailty and risk of fall
Clarkesmith et al. (2017); [44] educational and behavioural interventions	**Search:** update of 2013 review; February 2016 **Included:** 11 RCTs on AF; 2246 participants **Published:** 1999 to 2014 **Quality tool:** Cochrane and GRADE	**Patients: ** Small positive effects of education on anxiety (MD − 0.62, 95% CI − 1.21 to − 0.04; HADS score) and depression (MD − 0.74, 95% CI − 1.34 to − 0.14), compared with usual care, over 12 months. Decision aids had no significant impact on AF patients’ anxiety levels or satisfaction. One study found a decline in both anxiety and depression at 6-month follow-up. Patients may feel more anxious and depressed in the first few months after diagnosis
Entezari-Maleki et al. (2016); [45] pharmacist-managed warfarin therapy	**Search:** January 2014 **Included:** 24^a^ RCTs and non-RCTs on AF and VTE; 11,607 participants **Published:** 1995 to 2013 **Quality tool:** Downs and Black, and Jadad	**Patients: ** All patients in the pharmacist management group and 55% of the usual care group preferred pharmacist management. Patients believed that pharmacists were more expert in OAC control than their physicians. One study reported that health-related quality of life was similar between pharmacist and usual care
Generalova et al. (2018); [76] views and experiences of DOAC vs warfarin	**Search:** July 2017 **Included:** 10 studies, 1246 participants, NVAF **Published**: 2013 to 2016 **Quality tool:** STROBE and COREQ	**Clinicians**: DOAC perceived to be equally, or more, effective and safer than warfarin, particularly better for those who might miss appointments, but concerns about reversal and bleeding
Jang (2021) [55]; education on warfarin	**Search:** May 2020 **Included:** 12 studies, participants NR, AF or other condition **Published**: 2014 to 2020 **Quality tool:** Downs and Black	**Patients**: Education improves knowledge, adherence, satisfaction and clinical outcomes and a positive effect on continuing health care
Katerenchuk et al.^b^(2021) [74]; satisfaction with DOAC vs VKAs	**Search:** September 2019 **Included:** 20 studies, 18,684 participants, AF or VTE **Published**: 2013 to 2019 **Quality tool:** Cochrane, NOS and GRADE	**Patients:** Improvements in satisfaction score on switching to DOACs. Higher satisfaction on DOACs vs VKA. Mainly due to lower treatment burden with DOACs
Loewen et al. (2017); [69] values and preferences for treatment, and patient-specific factors that affect them	**Search:** September 2016 **Included:** 25 discrete-choice experiments; 641 participants **Published:** 1996 to 2016 **Quality tool:** CONSORT, STROBE, COREQ, ISPOR	**Patients: ** Stroke prevention was highly valued. After efficacy and safety, one versus two daily doses, antidote availability, absence of dietary restrictions and drug-drug interactions were moderately important, but this varied by study. Treatment choices were unpredictable. Cultural or family attitudes, beliefs, and personal experiences could affect OAC choice. As preferences varied, values and preferences should be discussed with patients
Mas Dalmau et al. (2017); [70] perceptions and attitudes to vitamin K antagonists, and factors related to underuse	**Search:** May 2013 **Included:** nine qualitative or mixed-methods studies; 250 patients and 91 physicians **Published:** 1999 to 2012 **Quality tool:** CASP	**Patients: ** Lack of information and understanding of OACs was patients’ main concern. The choice of OAC was determined by the individual’s experiences and values, as well as the downsides of treatment. The impact of treatment on daily life was important to patients **Practitioner: ** Physicians regarded the lack of a specific recommended OAC for each type of patient, the need for individual decision-making, and the delegated responsibility in decision-making as the main difficulties in using OACs. Some of the guidelines were ambiguous, and the included populations did not usually represent most patients (i.e., the very elderly). It was considered crucial to improve the quality of the information provided to patients because this was the main dissatisfaction with therapy
Pandya et al. (2017); [71] factors underpinning non-adherence	**Search**: NR **Included:** 47 surveys, interviews, or discrete-choice experiments on AF; 4151 participants **Published:** 1991 to 2014 **Quality tool**: NR	**Patients: ** The main reason for non-adherence was a lack of understanding about AF and stroke, and the importance of taking OACs. Reluctance to take warfarin was due to factors negatively affecting daily life (such as regular monitoring, dose adjustments, and diet). Some patients found it harder to accept, manage and adhere to direct OACs due to the absence of regular monitoring, limited access to antidotes, high costs of the medications, twice-daily dosing (dabigatran and apixaban) and timing of doses with respect to meals (dabigatran and rivaroxaban). Forgetfulness, attitudes toward stroke and bleeding risk, condition-related factors, social and economic factors, and healthcare system-related factors could affect adherence to direct OACs in a similar way to warfarin
Salmasi et al. (2019); [75] knowledge gaps on condition and treatment	**Search:** May 2018 **Included:** 21 studies, participants NR, AF **Published**: 2002 to 2018 **Quality tool:** STROBE and COREQ	**Patients:** Knowledge gaps on AF, stroke, medications, medical terms, and actions on missing a dose
Wilke et al. (2017); [72] preferences for OAC treatment	**Search:** 1980 to 2015 **Included:** 27 quantitative preference studies on AF; 7295 patients and 266 physicians **Published:** 1996 to 2016 **Quality tool**: unnamed	**Patients: ** AF patient preferences for OACs were inconsistent, except that some patients who did not mind a risk of bleeding chose the same OAC, while those who were more averse to bleeding preferred other OACs. Patients valued clinical attributes, such as bleeding risk, over convenience. Where OACs were similar in efficacy and safety, convenience, such as mode of application and availability of an antidote, affected choice
Willett and Morrill (2017) [73]; dosing for direct OACs, use in renal-impaired patients, and adherence, satisfaction and cost	**Search:** week 1, 2016 (MEDLINE) and week 2, 2017 (Embase) **Included:** 10 systematic reviews, trials or surveys on AF or VTE (nine cited); participants NR **Published:** 2001 to 2016 **Quality tool:** NR	**Patients: ** Most studies focussed on patients’ willingness to switch from warfarin to dabigatran or their satisfaction with dabigatran. Frequency of blood tests, along with dosing frequency and drug–food interactions, was less important than efficacy and safety. Cost was important; direct OACs became more attractive as their cost decreased. Adherence studies suggested that direct OACs that were taken daily were preferred over those taken twice daily
Zhou et al. (2016); [49] Pharmacist-managed anticoagulation control of warfarin	**Search:** July 2015 **Included:** eight RCTs; 1493 participants **Published:** 2003 to 2013 **Quality tool:** Cochrane and GRADE	**Patients: ** High satisfaction (MD 0.41, 95% CI 0.01 to 0.81) with pharmacist management was attributed to improved patient quality of life (e.g., self-efficacy, daily hassles, and distress), pharmacist service, interpersonal manner, communication, time spent, and accessibility. Pharmacists focussed on clinical counselling, patient education, home-visit monitoring, anticoagulation clinics, standardised follow-up, and comprehensive pharmaceutical care

*RCT* randomised controlled trial, *AF* atrial fibrillation, *OAC* oral anticoagulant, *NR* not reported, *NVAF* non-valvular atrial fibrillation, *GRADE* Grading of Recommendations Assessment, Development and Evaluation, *MD* mean difference, *CI* confidence interval, *CONSORT* Consolidated Standards of Reporting Trials, *STROBE* STrengthening the Reporting of OBservational studies in Epidemiology, *COREQ* consolidated criteria for reporting qualitative research, *ISPOR* International Society for Pharmacoeconomics and Outcomes Research, *CASP* Critical Appraisal Skills Programme, *VTE* venous thromboembolism, *OR* odds ratio, *DOAC* direct oral anticoagulant, *VKA* vitamin K antagonist

^a^Only six of these 24 studies (11,607 participants) were relevant to this part of the review

^b^No access to full text, but sufficient information in the abstract to include

From the original search, we included six reviews that focussed on patients with AF [44, 68–72] and three that included patients with a range of conditions including AF and VTE [45, 49, 73]. One review [73] focussed on direct OACs in patients with AF, VTE, or other conditions, specifically in patients with renal disease. From the update, we included three reviews that focussed on AF [51, 75, 76], two on AF and VTE [50, 74], and one on any condition [55]; four focussed on direct OACs (three compared with warfarin) [50, 51, 74, 76], and two included any OAC [55, 75].

Seven reviews investigated clinicians’ [76] or patients’ (and clinicians’ in two reviews) [68, 70] perceptions and attitudes to warfarin [70], warfarin, and direct OACs [68, 71, 74] or direct OACs [50, 73, 76]. Two reviews [69, 72] investigated patients’ (and physicians’ in one review) [72] experiences of vitamin K antagonists and direct OACs in discrete-choice experiments. One review [51] examined the reasons for discontinuation, given in patient records. The other five reviews examined patients’ knowledge gaps [75], or views of education or behavioural interventions [44, 55], or pharmacist management [45, 49]. Across the reviews, the number of included primary studies ranged between eight and 140, with between 341 and 11,607^1^ patients, where reported.

Seven reviews [44, 49, 51, 69, 70, 74, 76] met 10 of the 11 JBI criteria [18], and five [45, 50, 55, 59, 72] met nine, while one [71] met seven, one [68] met six, and one [73] met five criteria (see Additional file 9). Overall, we assessed the reviews to be at low risk of bias.

**Fig. 2:**
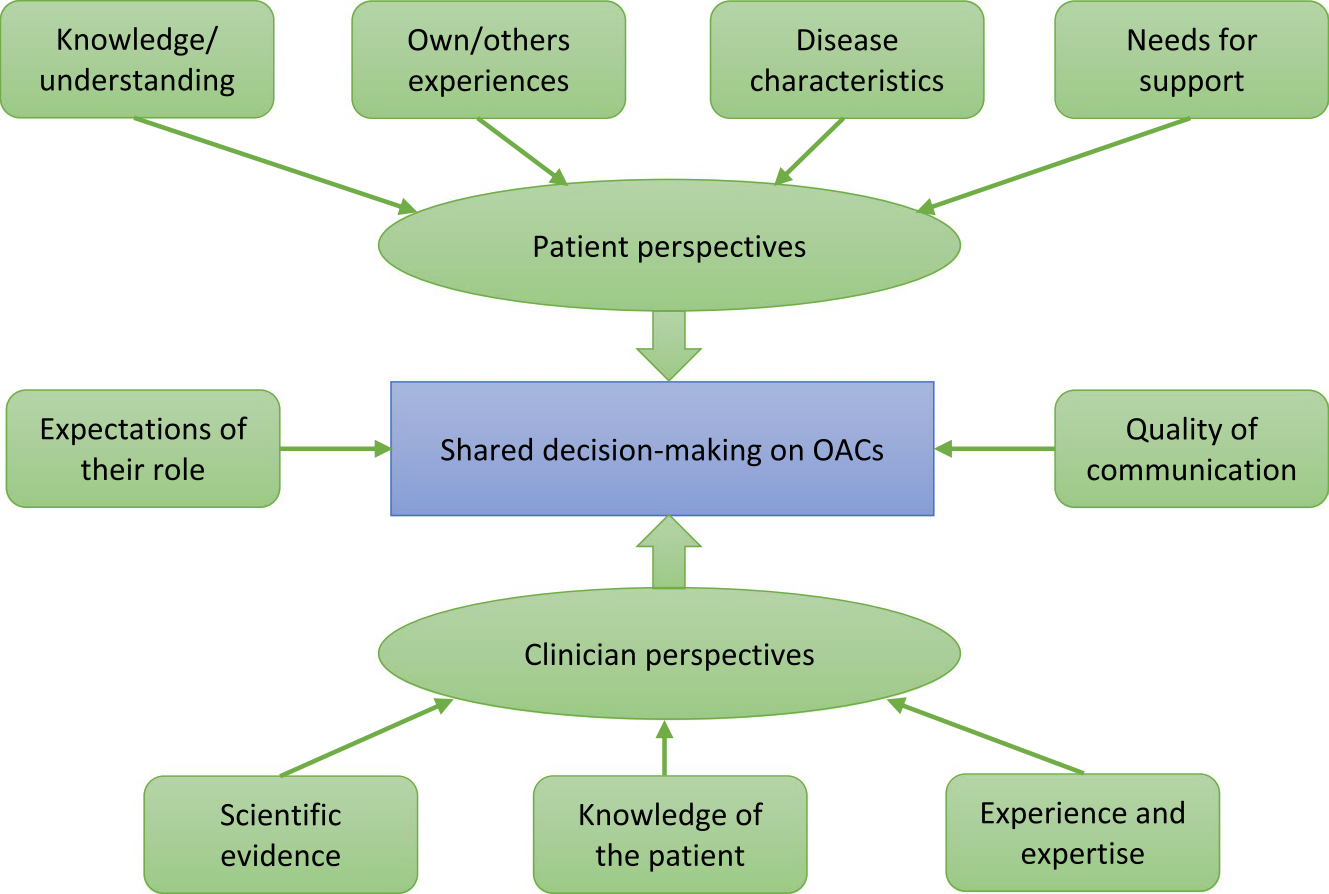
Themes influencing OAC decisions

Our synthesis of the original reviews showed that patients and physicians were most concerned with drug efficacy, followed by safety, except in one review [70] where geriatricians reported that safety was most important. Convenience or daily management factors were found to be important for adherence, although there was no consistent pattern across the reviews. A wide range of factors were reported as influencing patients’ decisions about starting, switching, or continuing OACs with no consistency about which were the most important for which groups of patients, at which point in their treatment. Figure 2 indicates the themes that were identified across these reviews. The evidence from the reviews identified in the update was consistent with these themes.

For patients, knowledge and the need for information influenced their decisions about which OAC to start with and whether or not to switch treatment. One review [44] suggested that improving knowledge could improve quality of life, anxiety, and depression. Past experience of stroke, bleeding, and/or OACs, and the experiences and support of families, also influenced patients’ decisions. Patients expressed a need for support and information in managing their therapy, and two reviews [45, 49] suggested that patients were more satisfied with pharmacist management than usual care. Three reviews found higher satisfaction with direct OACs than with warfarin, or with versus without education [50, 55, 74]. One review found gaps in knowledge about OACs and health conditions [75].

For clinicians, their knowledge of the patient, past experience, and expertise plus scientific evidence influenced their decisions. Poor communication between professionals (specialists and primary care physicians) who were involved in the patient’s care could complicate decision-making where their approaches differed [70]. One review [76], identified in the update, found that clinicians thought that direct OACs were safer, particularly for patients who missed appointments, but they had concerns about bleeding and reversal.

The review of medical notes [51] listed reasons for discontinuation that included bleeding, gastrointestinal events, frailty, and fall risk.

Where reviews included both clinicians and patients [68, 70, 72], two factors were found to influence decisions on oral anticoagulants: expectations of their role in decision-making (who was responsible for making the decision) and the quality of communication between clinician and patient. The use of decision aids did not improve patient satisfaction nor reduce decision conflict [44]. Both clinicians and patients wanted improved communication.

The 15 reviews included a total of 237 primary studies. Of these, 38 (16%) studies were included in two reviews, three were included in three reviews, and one was included in four reviews, suggesting slight overlap overall (CCA 1.4%).

## Discussion

### Summary of the evidence

One high-quality systematic review found that for the prevention of stroke in patients with AF (in accordance with NICE guidance), most direct OACs were more effective and safer than warfarin; apixaban 5 mg twice daily was most likely to be best and cost-effective (based on high- and low-quality primary studies). For the primary prevention of VTE (based on high-quality primary studies), there was no strong evidence that direct OACs should replace low-molecular-weight heparin (which is recommended by NICE for hip or knee replacement) [3]. For both the treatment and secondary prevention of VTE, there was little evidence that direct OACs were better than warfarin, but some of them may reduce the risks of bleeding (the 2020 NICE guidance recommends apixaban or rivaroxaban) [4]. For VTE treatment, apixaban (5 mg twice daily) was most likely to be cost-effective, and for secondary prevention, aspirin was most likely to be cost-effective (based on high-quality primary studies).

None of the reviews of genotyping, identified by the first search, reported results separately for patients with AF or VTE. In the update, one low risk-of-bias review focussed on stroke prevention in patients with AF and found no significant differences with genotype-guided warfarin dosing, compared with usual care. There remains a lack of evidence on genotype-guided dosing for patients with VTE. Most of the 23 reviews of self-monitoring were at moderate or low risk of bias. The evidence suggested that in patients with AF, education with or without a decision aid improved time in therapeutic range (based on high risk-of-bias primary studies), while self-management and self-testing had little effect (based on low-to-high-risk-of-bias primary studies); three of the reviews published in 2020 to 2021 found improvements in TTR based on evidence at high or medium risk of bias. Pharmacist management improved time in range (based on low risk-of-bias primary studies). Both adherence (PDC) and satisfaction were higher with direct OACs. Most of the 15 reviews of stakeholder experiences were at a low risk of bias. Based on primary studies of low-to-high or unclear risk of bias, efficacy was the main driver of the choice of OAC, followed by safety. Other factors were important (see Fig. 2), but these varied by patient and by time-point of decision. Patients were more satisfied with pharmacist management than with usual care, and with direct OACs than with warfarin.

#### Key messages

##### What is known about oral anticoagulants.

- The 2014 NICE guidelines recommended both warfarin and direct oral anticoagulants (OACs) to prevent and treat stroke related to atrial fibrillation (AF), and for venous thromboembolism (VTE).
- These guidelines were updated between 2018 and 2021, making direct OACs the first choice in some cases. This is in accordance with the evidence presented in our original 2018 overview.

##### What this systematic overview adds.

- For AF, direct OACs were more effective and safer than usual care, and apixaban 5 mg twice daily had the best profile. For VTE, overall, direct OACs were no better than low-molecular-weight heparin (prevention), warfarin (treatment), and warfarin or aspirin (secondary prevention).
- One review found no difference between genotype-guided warfarin dosing and direct OACs for stroke prevention in patients with AF. Reviews of genotype-guided dosing for direct OACs, in patients with AF or VTE, are needed.
- In patients with AF, education with or without a decision aid improved time in therapeutic range, while self-monitoring or self-testing made little difference. Evidence was lacking for patients with VTE. Pharmacist management could improve time in therapeutic range. Adherence was better with direct OACs than with warfarin.
- Efficacy was the main driver of the choice of OAC, followed by safety, except for geriatricians, where safety was more important than efficacy. For patients, it seems that knowledge, past experience, disease-related issues, and support needs influence OAC choices and adherence. Patients were more satisfied with pharmacist management, and with direct OACs, than with usual care.

### Strengths and limitations

Although this overview, commissioned to inform policy, was carried out within a short timescale, our processes were robust and key decisions were discussed with stakeholders. We updated our search to identify new evidence published since the original review was completed. A new review on genotyping in patients with AF, and a new review on satisfaction with direct oral anticoagulants, filled two evidence gaps. The main conclusions of the original review remain unchanged. This overview meets the reporting requirements of PRISMA (see Additional file 1). The included systematic reviews were rated as at low-to-moderate risk of bias, but the primary studies within the reviews had some limitations, for example, short follow-up; enrolling younger, healthier patients than would be found in usual practice; funding from drug manufacturers; and review authors assessed some of the primary studies as having a high risk of bias. Renally impaired patients were not specifically addressed by the included efficacy review; we identified a few reviews that may address this question (see the full report[13] and Additional file 5).

### Recommendations for research

None of the reviews examined the effects of switching from warfarin to direct OACs on the workload of monitoring clinics, which was an aim of the original review. Evidence is needed from trials that include the types of patients found in clinical practice, with long-term follow-up, that directly compare direct OACs, and that are not funded by drug manufacturers. Systematic reviews are needed on genotype-guided dosing of direct OACs for patients with AF or VTE, and on adherence to direct OACs for the treatment and prevention of VTE. The reasons for the limited effectiveness of decision aids could be investigated further. The views of older patients, particularly over 80 years of age, should be investigated. Further exploration, by gender, age, and ethnicity, of self-monitoring, adherence, and patient and clinician views of interventions would add value.

## Conclusion

Based on a comprehensive systematic overview of available reviews, the evidence suggests that direct OACs are safer and more effective than warfarin to prevent stroke in patients with AF, especially apixaban 5 mg twice daily. For VTE, there was no strong evidence that any direct OAC should replace those OACs currently recommended by NICE. Effectiveness of the treatment is the most important consideration for patients and clinicians, although older patients might be given the safest option. Genotype-guided dosing may not affect the risk of stroke in patients with AF. Pharmacist management of warfarin may be effective and patients preferred it over management in primary care. Patients were more satisfied with direct OACs than with warfarin. Educating patients about their condition and the use of OACs could improve their adherence and coagulation control.

## Supplementary Information

**Additional file 1. **PRISMA checklist.

**Additional file 2. **MEDLINE search strategy.

**Additional file 3. **Update search strategies.

**Additional file 4. **Genotyping review characteristics.

**Additional file 5. **Potential efficacy and safety includes identified using title and abstract from the update searches.

**Additional file 6. **Articles excluded at full-text assessment.

**Additional file 7. **Quality assessment of the included reviews for efficacy and safety, genotyping, and self-monitoring.

**Additional file 8. **Overlap in studies included in reviews for each section.

**Additional file 9. **Risk of bias assessment for reviews of stakeholder experiences

## Data Availability

All the data in this report are publically available, but not necessarily without charge.

## Abbreviations

AF: Atrial fibrillation
DHSC: Department of Health and Social Care
DVT: Deep-vein thrombosis
INR: International normalised ratio
NICE: National Institute for Health and Care Excellence
OAC: Oral anticoagulant
OECD: Organisation for Economic Co-operation and Development
PDC: Proportion of days covered
PE: Pulmonary embolism
RCT: Randomised controlled trial
TTR: Time in therapeutic range
VTE: Venous thromboembolism

## Footnotes

1 Only six of the 24 studies (11,607 participants) were relevant to this part of the review.
