# Supplementary material for: Oral anticoagulants: a systematic overview of reviews on efficacy and safety, genotyping, self-monitoring, and stakeholder experiences
Source: Syst Rev. 2022 Oct 28;11:232. doi: 10.1186/s13643-022-02098-w (PMC9615370; doi:10.1186/s13643-022-02098-w)
Supplement: Supplementary file 2 — Additional file 2. MEDLINE search strategy. [file 13643_2022_2098_MOESM2_ESM.docx]

Additional file 2: MEDLINE search strategy (October, 2017)

Database: Ovid MEDLINE(R) Epub Ahead of Print, In-Process & Other Non-Indexed Citations, Ovid MEDLINE(R) Daily and Ovid MEDLINE(R) <1946 to Present>

1 Anticoagulants/ (70885)

2 Administration, Oral/ (140536)

3 1 and 2 (5342)

4 (oral$ adj3 anticoagulant$).mp. (11057)

5 (oral$ adj3 anticoagulation).mp. (4884)

6 (OAC adj3 (treat$ or therap$)).ti,ab. (506)

7 DOAC$.mp. (818)

8 NOAC$.mp. (1588)

9 (warfarin or coumadin).ti,ab. (23229)

10 warfarin/ (18867)

11 (apixaban or eliquis).ti,ab. (2180)

12 Dabigatran/ (2503)

13 (dabigatran or pradaxa).ti,ab. (3813)

14 (edoxaban or lixiana).ti,ab. (894)

15 Rivaroxaban/ (2172)

16 (rivaroxaban or xarelto).ti,ab. (3482)

17 Aspirin/ (45105)

18 aspirin.ti,ab. (46772)

19 17 or 18 (65844)

20 Stroke/ or Ischaemic Attack, Transient/ (87442)

21 Myocardial Infarction/ or Venous Thrombosis/ (193741)

22 Thromboembolism/ or Pulmonary Embolism/ or Atrial Fibrillation/ (107659)

23 Anticoagulants/ (70885)

24 20 or 21 or 22 or 23 (410719)

25 19 and 24 (12727)

26 (aspirin adj3 (stroke$ or transient ischaemic attack$ or transient ischemic attack$ or TIA or heart attack$)).ti,ab. (822)

27 (aspirin adj3 (thrombosis or embolism or thromboembolism or atrial fibrillation)).ti,ab. (337)

28 (aspirin adj3 anticoagul$).ti,ab. (689)

29 "Vitamin K"/ (11631)

30 vitamin K.ti,ab. (13391)

31 29 or 30 (19806)

32 31 and 24 (5825)

33 (vitamin K adj3 (stroke$ or transient ischaemic attack$ or transient ischemic attack$ or TIA or heart attack$)).ti,ab. (67)

34 (VKA$ adj3 (stroke$ or transient ischaemic attack$ or transient ischemic attack$ or TIA or heart attack$)).ti,ab. (62)

35 (vitamin K adj3 (thrombosis or embolism or atrial fibrillation)).ti,ab. (125)

36 (VKA$ adj3 (thrombosis or embolism or thromboembolism or atrial fibrillation)).ti,ab. (67)

37 (vitamin K adj3 anticoagul$).ti,ab. (1549)

38 vitamin K antagonist$.ti,ab. (4736)

39 3 or 4 or 5 or 6 or 7 or 8 or 9 or 10 or 11 or 12 or 13 or 14 or 15 or 16 or 25 or 26 or 27 or 28 or 32 or 33 or 34 or 35 or 37 or 38 (54568)

40 Meta-Analysis as Topic/ (17376)

41 meta analy$.tw. (132487)

42 metaanaly$.tw. (1966)

43 Meta-Analysis/ (94843)

44 (systematic adj (review$1 or overview$1)).tw. (121548)

45 exp Review Literature as Topic/ (10322)

46 or/40-45 (237989)

47 cochrane.ab. (61716)

48 embase.ab. (65920)

49 (psychlit or psyclit).ab. (957)

50 (psychinfo or psycinfo).ab. (22044)

51 (cinahl or cinhal).ab. (20870)

52 science citation index.ab. (2933)

53 cancerlit.ab. (679)

54 or/47-53 (106215)

55 reference list$.ab. (15901)

56 bibliograph$.ab. (16317)

57 hand-search$.ab. (6091)

58 relevant journals.ab. (1104)

59 manual search$.ab. (3840)

60 55 or 56 or 57 or 58 or 59 (38732)

61 selection criteria.ab. (28310)

62 data extraction.ab. (16717)

63 61 or 62 (42844)

64 Review/ (2480860)

65 63 and 64 (28687)

66 comment/ (735999)

67 letter/ (1035172)

68 editorial/ (470242)

69 animal/ (6598857)

70 human/ (18058112)

71 69 not (69 and 70) (4708497)

72 66 or 67 or 68 or 71 (6336983)

73 46 or 54 or 60 or 65 (284883)

74 73 not 72 (270201)

75 39 and 74 (2082)

76 qualitative systematic review$.ti,ab. (511)

77 (systematic review and qualitative).ti,ab. (5075)

78 evidence synthesis.ti,ab. (3012)

79 realist synthesis.ti,ab. (155)

80 (qualitative and synthesis).ti,ab. (5761)

81 (meta-synthesis$ or meta synthesis$ or metasynthesis$).ti,ab. (868)

82 (meta-ethnograph$ or metaethnograph$ or meta ethnograph$).ti,ab. (412)

83 (meta-study or metastudy or meta study).ti,ab. (85)

84 76 or 77 or 78 or 79 or 80 or 81 or 82 or 83 (12431)

85 39 and 84 (23)

86 75 or 85 (2088)

87 limit 86 to yr="2014 -Current" (895)
