# Supplementary material for: Oral anticoagulants: a systematic overview of reviews on efficacy and safety, genotyping, self-monitoring, and stakeholder experiences
Source: Syst Rev. 2022 Oct 28;11:232. doi: 10.1186/s13643-022-02098-w (PMC9615370; doi:10.1186/s13643-022-02098-w)

**Additional file 3: Update search strategies (November 2021)**

Update searches were carried out on all the databases that were originally searched, with the exception of DARE, which closed in March 2015. All original search strategies were checked, and any additional subject headings added, before re-running. In total, 8117 records were retrieved. These records were imported into EPPI-Reviewer and deduplicated against each other (between sources for the new searches), and against the original search results.

**Search strategies:**

**Ovid MEDLINE(R) ALL** <1946 to November 29, 2021>

Searched on: 30^th^ November 2021

Records retrieved: 2094

1 Anticoagulants/ (82019)

2 Administration, Oral/ (149926)

3 1 and 2 (7781)

4 (oral$ adj3 anticoagulant$).mp. (16992)

5 (oral$ adj3 anticoagulation).mp. (6428)

6 (OAC adj3 (treat$ or therap$)).ti,ab. (862)

7 DOAC$.mp. (3234)

8 NOAC$.mp. (2812)

9 (warfarin or coumadin).ti,ab. (26127)

10 Warfarin/ (20478)

11 (apixaban or eliquis).ti,ab. (3870)

12 Dabigatran/ (3504)

13 (dabigatran or pradaxa).ti,ab. (5169)

14 (edoxaban or lixiana).ti,ab. (1592)

15 Rivaroxaban/ (3989)

16 (rivaroxaban or xarelto).ti,ab. (5980)

17 Aspirin/ (46715)

18 aspirin.ti,ab. (50976)

19 17 or 18 (70023)

20 Stroke/ or Ischemic Attack, Transient/ (131139)

21 Myocardial Infarction/ or Venous Thrombosis/ (199134)

22 Thromboembolism/ or Pulmonary Embolism/ or Atrial Fibrillation/ (123249)

23 Anticoagulants/ (82019)

24 20 or 21 or 22 or 23 (474764)

25 19 and 24 (13967)

26 (aspirin adj3 (stroke$ or transient ischaemic attack$ or transient ischemic attack$ or TIA or heart attack$)).ti,ab. (876)

27 (aspirin adj3 (thrombosis or embolism or thromboembolism or atrial fibrillation)).ti,ab. (390)

28 (aspirin adj3 anticoagul$).ti,ab. (792)

29 Vitamin K/ (12237)

30 vitamin K.ti,ab. (16368)

31 29 or 30 (22572)

32 24 and 31 (7757)

33 (vitamin K adj3 (stroke$ or transient ischaemic attack$ or transient ischemic attack$ or TIA or heart attack$)).ti,ab. (87)

34 (VKA$ adj3 (stroke$ or transient ischaemic attack$ or transient ischemic attack$ or TIA or heart attack$)).ti,ab. (112)

35 (vitamin K adj3 (thrombosis or embolism or thromboembolism or atrial fibrillation)).ti,ab. (229)

36 (VKA$ adj3 (thrombosis or embolism or thromboembolism or atrial fibrillation)).ti,ab. (102)

37 (vitamin K adj3 anticoagul$).ti,ab. (2773)

38 vitamin K antagonist$.ti,ab. (6860)

39 3 or 4 or 5 or 6 or 7 or 8 or 9 or 10 or 11 or 12 or 13 or 14 or 15 or 16 or 25 or 26 or 27 or 28 or 32 or 33 or 34 or 35 or 36 or 37 or 38 (65927)

40 "systematic review"/ (177924)

41 network meta-analysis/ (3047)

42 40 or 41 (179681)

43 meta-analysis as topic/ (20561)

44 meta analy$.tw. (217275)

45 metaanaly$.tw. (2348)

46 Meta-Analysis/ (147970)

47 (systematic adj (review$1 or overview$1)).tw. (225082)

48 exp Review Literature as Topic/ (18334)

49 or/43-48 (372526)

50 cochrane.ab. (106230)

51 embase.ab. (119507)

52 (psychlit or psyclit).ab. (916)

53 (psychinfo or psycinfo).ab. (46048)

54 (cinahl or cinhal).ab. (36026)

55 science citation index.ab. (3396)

56 cancerlit.ab. (636)

57 or/50-56 (192331)

58 reference list$.ab. (20066)

59 bibliograph$.ab. (20205)

60 hand-search$.ab. (7739)

61 relevant journals.ab. (1263)

62 manual search$.ab. (5163)

63 or/58-62 (48838)

64 selection criteria.ab. (33064)

65 data extraction.ab. (26139)

66 64 or 65 (56728)

67 Review/ (2902074)

68 66 and 67 (31023)

69 comment/ (940176)

70 letter/ (1160667)

71 editorial/ (588129)

72 animal/ (6977363)

73 human/ (19954674)

74 72 not (72 and 73) (4889136)

75 69 or 70 or 71 or 74 (6833732)

76 42 or 49 or 57 or 63 or 68 (452379)

77 76 not 75 (429831)

78 39 and 77 (3225)

79 qualitative systematic review$.ti,ab. (996)

80 (systematic review and qualitative).ti,ab. (11233)

81 evidence synthesis.ti,ab. (5427)

82 realist synthesis.ti,ab. (277)

83 (qualitative and synthesis).ti,ab. (10282)

84 (meta-synthesis$ or meta synthesis$ or metasynthesis$).ti,ab. (1514)

85 (meta-ethnograph$ or metaethnograph$ or meta ethnograph$).ti,ab. (698)

86 (meta-study or metastudy or meta study).ti,ab. (122)

87 79 or 80 or 81 or 82 or 83 or 84 or 85 or 86 (22767)

88 39 and 87 (44)

89 78 or 88 (3232)

90 limit 89 to yr="2014 -Current" (2094)

**Embase** via Ovid <1974 to 2021 November 29>

Searched on: 30th November 2021

Records retrieved: 4567

1 anticoagulant agent/ (115707)

2 oral drug administration/ (391529)

3 1 and 2 (4456)

4 (oral$ adj3 anticoagulant$).mp. (28729)

5 (oral$ adj3 anticoagulation).mp. (11429)

6 (OAC adj3 (treat$ or therap$)).ti,ab. (1780)

7 DOAC$.mp. (6358)

8 NOAC$.mp. (5837)

9 (warfarin or coumadin).ti,ab. (43988)

10 warfarin/ (97883)

11 apixaban/ (15631)

12 (apixaban or eliquis).ti,ab. (8543)

13 dabigatran/ (15998)

14 (dabigatran or pradaxa).ti,ab. (10514)

15 edoxaban/ (5865)

16 (edoxaban or lixiana).ti,ab. (2947)

17 rivaroxaban/ (21885)

18 (rivaroxaban or xarelto).ti,ab. (12585)

19 acetylsalicylic acid/ (225346)

20 aspirin.ti,ab. (78351)

21 19 or 20 (234431)

22 cerebrovascular accident/ or transient ischemic attack/ (263194)

23 heart infarction/ or vein thrombosis/ (319219)

24 thromboembolism/ or arterial thromboembolism/ or venous thromboembolism/ or lung embolism/ or atrial fibrillation/ (267189)

25 anticoagulant agent/ (115707)

26 22 or 23 or 24 or 25 (820586)

27 21 and 26 (66488)

28 (aspirin adj3 (stroke$ or transient ischaemic attack$ or transient ischemic attack$ or TIA$ or heart attack$)).ti,ab. (1323)

29 (aspirin adj3 (thrombosis or embolism or thromboembolism or atrial fibrillation)).ti,ab. (538)

30 (aspirin adj3 anticoagul$).ti,ab. (1406)

31 antivitamin K/ (16623)

32 vitamin K.ti,ab. (23874)

33 31 or 32 (31993)

34 26 and 33 (17992)

35 (vitamin K adj3 (stroke$ or transient ischaemic attack$ or transient ischemic attack$ or TIA$ or heart attack$)).ti,ab. (139)

36 (VKA$ adj3 (stroke$ or transient ischaemic attack$ or transient ischemic attack$ or TIA$ or heart attack$)).ti,ab. (235)

37 (vitamin K adj3 (thrombosis or embolism or thromboembolism or atrial fibrillation)).ti,ab. (371)

38 (VKA$ adj3 (thrombosis or embolism or thromboembolism or atrial fibrillation)).ti,ab. (218)

39 (vitamin K adj3 anticoagul$).ti,ab. (4580)

40 vitamin K antagonist$.ti,ab. (11742)

41 3 or 4 or 5 or 6 or 7 or 8 or 9 or 10 or 11 or 12 or 13 or 14 or 15 or 16 or 17 or 18 or 27 or 28 or 29 or 30 or 34 or 35 or 36 or 37 or 38 or 39 or 40 (191612)

42 exp meta analysis/ (231558)

43 ((meta adj analy$) or metaanalys$).tw. (281390)

44 (systematic adj (review$1 or overview$1)).tw. (273753)

45 or/42-44 (467400)

46 cancerlit.ab. (740)

47 cochrane.ab. (135790)

48 embase.ab. (149320)

49 (psychlit or psyclit).ab. (1005)

50 (psychinfo or psycinfo).ab. (44026)

51 (cinahl or cinhal).ab. (42012)

52 science citation index.ab. (3920)

53 bids.ab. (738)

54 or/46-53 (231626)

55 reference lists.ab. (21507)

56 bibliograph$.ab. (25615)

57 hand-search$.ab. (9423)

58 manual search$.ab. (6161)

59 relevant journals.ab. (1506)

60 or/55-59 (57860)

61 data extraction.ab. (31809)

62 selection criteria.ab. (40639)

63 61 or 62 (69993)

64 review.pt. (2821153)

65 63 and 64 (32999)

66 letter.pt. (1198500)

67 editorial.pt. (708653)

68 animal/ (1535710)

69 human/ (22824990)

70 68 not (68 and 69) (1125235)

71 or/66-67,70 (3014604)

72 45 or 54 or 60 or 65 (553456)

73 72 not 71 (539101)

74 41 and 72 (8924)

75 qualitative systematic review$.ti,ab. (1082)

76 (systematic review and qualitative).ti,ab. (13075)

77 evidence synthesis.ti,ab. (5945)

78 realist synthesis.ti,ab. (270)

79 (qualitative and synthesis).ti,ab. (11457)

80 (meta-synthesis$ or meta synthesis$ or metasynthesis$).ti,ab. (1677)

81 (meta-ethnograph$ or metaethnograph$ or meta ethnograph$).ti,ab. (781)

82 (meta-study or metastudy or meta study).ti,ab. (140)

83 or/75-82 (25862)

84 41 and 83 (108)

85 74 or 84 (8941)

86 limit 85 to yr="2014 -Current" (4567)

**CINAHL Plus**

via Ebsco

Inception to 29^th^ November 2021

Searched on: 30th November 2021

Records retrieved: 1333

| S1 | (MH "Anticoagulants") | 21,394 |
| --- | --- | --- |
| S2 | (MH "Administration, Oral") | 24,028 |
| S3 | S1 AND S2 | 2,321 |
| S4 | TI ORAL* N3 ANTICOAGULANT* OR AB ORAL* N3 ANTICOAGULANT* | 4,994 |
| S5 | TI ORAL* N3 ANTICOAGULATION OR AB ORAL* N3 ANTICOAGULATION | 1,796 |
| S6 | TI ( OAC N3 (therap* or treat*) ) OR AB ( OAC N3 (therap* or treat*) ) | 277 |
| S7 | TI ( DOAC* OR NOAC* ) OR AB ( DOAC* OR NOAC* ) | 1,735 |
| S8 | S3 OR S4 OR S5 OR S6 OR S7 | 7,022 |
| S9 | (MH "Warfarin") | 8,460 |
| S10 | TI ( warfarin or coumadin ) OR AB ( warfarin or coumadin ) | 8,456 |
| S11 | TI ( apixaban or eliquis ) OR AB ( apixaban or eliquis ) | 1,473 |
| S12 | (MH "Dabigatran Etexilate") | 706 |
| S13 | TI ( dabigatran or pradaxa ) OR AB ( dabigatran or pradaxa ) | 2,226 |
| S14 | TI ( edoxaban or lixiana ) OR AB ( edoxaban or lixiana ) | 556 |
| S15 | (MH "Rivaroxaban") | 699 |
| S16 | TI ( rivaroxaban or xarelto ) OR AB ( rivaroxaban or xarelto ) | 2,357 |
| S17 | S9 OR S10 OR S11 OR S12 OR S13 OR S14 OR S15 OR S16 | 14,843 |
| S18 | S8 OR S17 | 18,630 |
| S19 | (MH "Aspirin") | 12,715 |
| S20 | TI aspirin OR AB aspirin | 12,782 |
| S21 | S19 OR S20 | 17,992 |
| S22 | (MH "Stroke") | 74,493 |
| S23 | (MH "Cerebral Ischemia, Transient") | 5,263 |
| S24 | (MH "Myocardial Infarction") | 46,499 |
| S25 | (MH "Venous Thrombosis") | 10,340 |
| S26 | (MH "Thromboembolism") | 5,892 |
| S27 | (MH "Pulmonary Embolism") | 10,512 |
| S28 | (MH "Atrial Fibrillation") | 27,460 |
| S29 | (MH "Anticoagulants") | 21,394 |
| S30 | S22 OR S23 OR S24 OR S25 OR S26 OR S27 OR S28 OR S29 | 170,991 |
| S31 | S21 AND S30 | 5,698 |
| S32 | TI ( aspirin N3 (stroke* or "transient ischaemic attack*" or "transient ischemic attack*" or TIA* or "heart attack*") ) OR AB ( aspirin N3 (stroke* or "transient ischaemic attack*" or "transient ischemic attack*" or TIA* or "heart attack*") ) | 582 |
| S33 | TI ( aspirin N3 (thrombosis or embolism or thromboembolism or "atrial fibrillation") ) OR AB ( aspirin N3 (thrombosis or embolism or thromboembolism or "atrial fibrillation") ) | 197 |
| S34 | TI aspirin N3 anticoagul* OR AB aspirin N3 anticoagul* | 312 |
| S35 | S32 OR S33 OR S34 | 1,039 |
| S36 | (MH "Vitamin K") | 3,016 |
| S37 | TI "vitamin K" OR AB "vitamin K" | 3,784 |
| S38 | S36 OR S37 | 5,114 |
| S39 | S30 AND S38 | 2,241 |
| S40 | TI ( "vitamin K" N3 (stroke* or "transient ischaemic attack*" or "transient ischemic attack*" or TIA* or "heart attack*") ) OR AB ( "vitamin K" N3 (stroke* or "transient ischaemic attack*" or "transient ischemic attack*" or TIA* or "heart attack*") ) OR TI ( VKA* N3 (stroke* or "transient ischaemic attack*" or "transient ischemic attack*" or TIA* or "heart attack*") ) OR AB ( VKA* N3 (stroke* or "transient ischaemic attack*" or "transient ischemic attack*" or TIA* or "heart attack*") ) | 103 |
| S41 | TI ( "vitamin K" N3 (thrombosis or embolism or thromboembolism or "atrial fibrillation") ) OR AB ( "vitamin K" N3 (thrombosis or embolism or thromboembolism or "atrial fibrillation") ) OR TI ( VKA* N3 (thrombosis or embolism or thromboembolism or "atrial fibrillation") ) OR AB ( VKA* N3 (thrombosis or embolism or thromboembolism or "atrial fibrillation") ) | 149 |
| S42 | TI "vitamin K" N3 anticoagul* OR TI "vitamin K antagonist*" OR AB "vitamin K" N3 anticoagul* OR AB "vitamin K antagonist*" | 2,078 |
| S43 | S39 OR S40 OR S41 OR S42 | 2,800 |
| S44 | S18 OR S31 OR S35 OR S43 | 23,529 |
| S45 | (MH "Meta Analysis") | 58,934 |
| S46 | TX Meta analys* OR TX Metaanaly* | 223,814 |
| S47 | (MH "Literature Review+") | 115,209 |
| S48 | TX systematic N1 (review or overview) | 322,076 |
| S49 | S45 OR S46 OR S47 OR S48 | 414,698 |
| S50 | S44 AND S49 | 2,363 |
| S51 | TX "qualitative systematic review*" OR TX ( "systematic review" and qualitative ) OR TX "evidence synthesis" OR TX "realist synthesis" OR TX ( qualitative and synthesis ) OR TX ( meta-synthesis* or "meta synthesis*" or metasynthesis* ) OR TX ( meta-ethnograph* or metaethnograph* or "meta ethnograph*" ) OR TX ( meta-study or metastudy or "meta study" ) | 70,476 |
| S52 | S44 AND S51 | 97 |
| S53 | S50 OR S52 Limiters - Published Date: 20140101-20211231 | 1,333 |

**ASSIA**

via ProQuest

Searched on: 30^th^ November 2021

Records retrieved: 116

| Set# | Searched for | Databases | Results |
| --- | --- | --- | --- |
| S1 | MAINSUBJECT.EXACT.EXPLODE("Anticoagulant drugs") | Applied Social Sciences Index & Abstracts (ASSIA) | 45 |
| S2 | (MAINSUBJECT.EXACT.EXPLODE("Anticoagulant drugs") OR ((oral* NEAR/3 anticoagulant*) OR (oral* NEAR/3 anticoagulation) OR (OAC NEAR/3 (treat* or therap*)) OR DOAC* OR NOAC*) OR (MAINSUBJECT.EXACT("Warfarin") OR (warfarin OR coumadin)) OR ((apixaban or eliquis) OR (dabigatran or pradaxa) OR (edoxaban or lixiana) OR (rivaroxaban or xarelto))) OR ((MAINSUBJECT.EXACT("Aspirin") OR aspirin) AND (MAINSUBJECT.EXACT("Strokes") OR MAINSUBJECT.EXACT("Transient ischaemic attacks") OR MAINSUBJECT.EXACT("Heart attacks") OR (MAINSUBJECT.EXACT("Deep vein thrombosis") OR MAINSUBJECT.EXACT("Thrombosis") OR MAINSUBJECT.EXACT("Arterial thrombosis") OR MAINSUBJECT.EXACT("Venous thrombosis")) OR MAINSUBJECT.EXACT("Pulmonary embolism") OR MAINSUBJECT.EXACT("Atrial fibrillation"))) OR ((aspirin NEAR/3 stroke* ) OR (aspirin NEAR/3 "transient ischaemic attack") OR (aspirin NEAR/3 "transient ischemic attack") OR (aspirin NEAR/3 TIA*) OR (aspirin NEAR/3 "heart attack") OR (aspirin NEAR/3 (thrombosis or embolism or thromboembolism or "atrial fibrillation" or anticoagul*))) OR ((MAINSUBJECT.EXACT("Vitamin K") OR "vitamin K") AND (MAINSUBJECT.EXACT("Strokes") OR MAINSUBJECT.EXACT("Transient ischaemic attacks") OR MAINSUBJECT.EXACT("Heart attacks") OR (MAINSUBJECT.EXACT("Deep vein thrombosis") OR MAINSUBJECT.EXACT("Thrombosis") OR MAINSUBJECT.EXACT("Arterial thrombosis") OR MAINSUBJECT.EXACT("Venous thrombosis")) OR MAINSUBJECT.EXACT("Pulmonary embolism") OR MAINSUBJECT.EXACT("Atrial fibrillation"))) OR (("vitamin k" NEAR/3 stroke*) OR ("vitamin k" NEAR/3 "transient ischaemic attack") OR ("vitamin k" NEAR/3 "transient ischemic attack") OR ("vitamin k" NEAR/3 TIA) OR ("vitamin k" NEAR/3 "heart attack") OR ("vitamin K" NEAR/3 (thrombosis or embolism or thromboembolism or fibrillation or anticoagul*)) OR "vitamin K antagonist*") | Applied Social Sciences Index & Abstracts (ASSIA) | 1825 |
| S3 | MAINSUBJECT.EXACT("Meta-analysis") OR (Meta analys* OR Metaanaly* ) OR MAINSUBJECT.EXACT.EXPLODE("Literature reviews") OR (systematic N1 (review or overview) ) OR "evidence synthesis" OR "realist synthesis" OR "qualitative synthesis" OR (meta-synthesis* or "meta synthesis*" or metasynthesis) OR (meta-ethnograph* or metaethnograph* or "meta ethnograph*" ) OR (meta-study or metastudy or "meta study") | Applied Social Sciences Index & Abstracts (ASSIA) | 64989 |
| S4 | ((MAINSUBJECT.EXACT.EXPLODE("Anticoagulant drugs") OR ((oral* NEAR/3 anticoagulant*) OR (oral* NEAR/3 anticoagulation) OR (OAC NEAR/3 (treat* OR therap*)) OR DOAC* OR NOAC*) OR (MAINSUBJECT.EXACT("Warfarin") OR (warfarin OR coumadin)) OR ((apixaban OR eliquis) OR (dabigatran OR pradaxa) OR (edoxaban OR lixiana) OR (rivaroxaban OR xarelto))) OR ((MAINSUBJECT.EXACT("Aspirin") OR aspirin) AND (MAINSUBJECT.EXACT("Strokes") OR MAINSUBJECT.EXACT("Transient ischaemic attacks") OR MAINSUBJECT.EXACT("Heart attacks") OR (MAINSUBJECT.EXACT("Deep vein thrombosis") OR MAINSUBJECT.EXACT("Thrombosis") OR MAINSUBJECT.EXACT("Arterial thrombosis") OR MAINSUBJECT.EXACT("Venous thrombosis")) OR MAINSUBJECT.EXACT("Pulmonary embolism") OR MAINSUBJECT.EXACT("Atrial fibrillation"))) OR ((aspirin NEAR/3 stroke*) OR (aspirin NEAR/3 "transient ischaemic attack") OR (aspirin NEAR/3 "transient ischemic attack") OR (aspirin NEAR/3 TIA*) OR (aspirin NEAR/3 "heart attack") OR (aspirin NEAR/3 (thrombosis OR embolism OR thromboembolism OR "atrial fibrillation" OR anticoagul*))) OR ((MAINSUBJECT.EXACT("Vitamin K") OR "vitamin K") AND (MAINSUBJECT.EXACT("Strokes") OR MAINSUBJECT.EXACT("Transient ischaemic attacks") OR MAINSUBJECT.EXACT("Heart attacks") OR (MAINSUBJECT.EXACT("Deep vein thrombosis") OR MAINSUBJECT.EXACT("Thrombosis") OR MAINSUBJECT.EXACT("Arterial thrombosis") OR MAINSUBJECT.EXACT("Venous thrombosis")) OR MAINSUBJECT.EXACT("Pulmonary embolism") OR MAINSUBJECT.EXACT("Atrial fibrillation"))) OR (("vitamin k" NEAR/3 stroke*) OR ("vitamin k" NEAR/3 "transient ischaemic attack") OR ("vitamin k" NEAR/3 "transient ischemic attack") OR ("vitamin k" NEAR/3 TIA) OR ("vitamin k" NEAR/3 "heart attack") OR ("vitamin K" NEAR/3 (thrombosis OR embolism OR thromboembolism OR fibrillation OR anticoagul*)) OR "vitamin K antagonist*")) AND (MAINSUBJECT.EXACT("Meta-analysis") OR (Meta analys* OR Metaanaly*) OR MAINSUBJECT.EXACT.EXPLODE("Literature reviews") OR (systematic N1 (review OR overview)) OR "evidence synthesis" OR "realist synthesis" OR "qualitative synthesis" OR (meta-synthesis* OR "meta synthesis*" OR metasynthesis) OR (meta-ethnograph* OR metaethnograph* OR "meta ethnograph*") OR (meta-study OR metastudy OR "meta study")) | Applied Social Sciences Index & Abstracts (ASSIA)  These databases are searched for part of your query. | 395 |
| S5 | ((MAINSUBJECT.EXACT.EXPLODE("Anticoagulant drugs") OR ((oral* NEAR/3 anticoagulant*) OR (oral* NEAR/3 anticoagulation) OR (OAC NEAR/3 (treat* OR therap*)) OR DOAC* OR NOAC*) OR (MAINSUBJECT.EXACT("Warfarin") OR (warfarin OR coumadin)) OR ((apixaban OR eliquis) OR (dabigatran OR pradaxa) OR (edoxaban OR lixiana) OR (rivaroxaban OR xarelto))) OR ((MAINSUBJECT.EXACT("Aspirin") OR aspirin) AND (MAINSUBJECT.EXACT("Strokes") OR MAINSUBJECT.EXACT("Transient ischaemic attacks") OR MAINSUBJECT.EXACT("Heart attacks") OR (MAINSUBJECT.EXACT("Deep vein thrombosis") OR MAINSUBJECT.EXACT("Thrombosis") OR MAINSUBJECT.EXACT("Arterial thrombosis") OR MAINSUBJECT.EXACT("Venous thrombosis")) OR MAINSUBJECT.EXACT("Pulmonary embolism") OR MAINSUBJECT.EXACT("Atrial fibrillation"))) OR ((aspirin NEAR/3 stroke*) OR (aspirin NEAR/3 "transient ischaemic attack") OR (aspirin NEAR/3 "transient ischemic attack") OR (aspirin NEAR/3 TIA*) OR (aspirin NEAR/3 "heart attack") OR (aspirin NEAR/3 (thrombosis OR embolism OR thromboembolism OR "atrial fibrillation" OR anticoagul*))) OR ((MAINSUBJECT.EXACT("Vitamin K") OR "vitamin K") AND (MAINSUBJECT.EXACT("Strokes") OR MAINSUBJECT.EXACT("Transient ischaemic attacks") OR MAINSUBJECT.EXACT("Heart attacks") OR (MAINSUBJECT.EXACT("Deep vein thrombosis") OR MAINSUBJECT.EXACT("Thrombosis") OR MAINSUBJECT.EXACT("Arterial thrombosis") OR MAINSUBJECT.EXACT("Venous thrombosis")) OR MAINSUBJECT.EXACT("Pulmonary embolism") OR MAINSUBJECT.EXACT("Atrial fibrillation"))) OR (("vitamin k" NEAR/3 stroke*) OR ("vitamin k" NEAR/3 "transient ischaemic attack") OR ("vitamin k" NEAR/3 "transient ischemic attack") OR ("vitamin k" NEAR/3 TIA) OR ("vitamin k" NEAR/3 "heart attack") OR ("vitamin K" NEAR/3 (thrombosis OR embolism OR thromboembolism OR fibrillation OR anticoagul*)) OR "vitamin K antagonist*")) AND (MAINSUBJECT.EXACT("Meta-analysis") OR (Meta analys* OR Metaanaly*) OR MAINSUBJECT.EXACT.EXPLODE("Literature reviews") OR (systematic N1 (review OR overview)) OR "evidence synthesis" OR "realist synthesis" OR "qualitative synthesis" OR (meta-synthesis* OR "meta synthesis*" OR metasynthesis) OR (meta-ethnograph* OR metaethnograph* OR "meta ethnograph*") OR (meta-study OR metastudy OR "meta study")) AND pd(20140101-20211130) | Applied Social Sciences Index & Abstracts (ASSIA)  These databases are searched for part of your query. | 116 |

**HTA Database**

via CRD Databases <https://www.crd.york.ac.uk/CRDWeb/>

Search date: 30^th^ November 2021

Records retrieved: 7

The HTA database closed in 2018, therefore, the update search was limited to records with a publication year between 2014 and 2018.


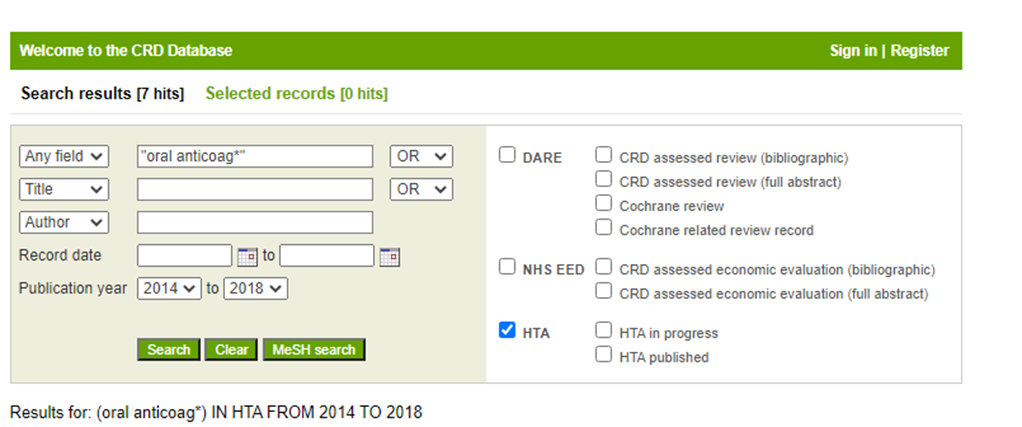

Supplement: Supplementary file 3 — Additional file 3. Update search strategies. [file 13643_2022_2098_MOESM3_ESM.docx]
