# Supplementary material for: Oral anticoagulants: a systematic overview of reviews on efficacy and safety, genotyping, self-monitoring, and stakeholder experiences
Source: Syst Rev. 2022 Oct 28;11:232. doi: 10.1186/s13643-022-02098-w (PMC9615370; doi:10.1186/s13643-022-02098-w)
Supplement: Supplementary file 4 — Additional file 4. Genotyping review characteristics. [file 13643_2022_2098_MOESM4_ESM.docx]

**Additional file 4**. Genotyping review characteristics

Patients with AF

| **Review authors (year), genes and OAC** | **Methods and study details** | **Primary outcomes** |
| --- | --- | --- |
| Ng et al. (2020),^1^ not specified;* warfarin | **Search**: November 2017  **Included**: 37 studies, 100142 patients with AF  **Published**: 1989 to 2017  **Quality tool:** Cochrane, GRADE | Stroke, mortality, major bleeding |

* the references suggested this was CYP2C9

****Patients with any condition requiring OAC; original searches****

| **Review authors (year), genes and OAC** | **Methods and study details** | **Primary outcomes** |
| --- | --- | --- |
| Chen et al. (2016),^2^ CYP4F2*3 and CYP4F2*1; coumarin | **Search**: not reported  **Included**: eight case-control or cohort studies; seven on AF/VTE; 3,101 adults  **Published**: 2009 to 2014 | Total haemorrhage, major haemorrhage, INR<4, and over-anticoagulation |
| Dahal et al. (2015),^3^ CYP2C9*2 and *3, VKORC1, CYP2C9, and CYP4F2; warfarin | **Search**: March 2014  **Included**: 10 RCTs; nine on AF/VTE; 2,505 adults  **Published**: 2005 to 2013 | Percentage of time in therapeutic INR range (TTR) |
| Franchini et al. (2014),^4^ CYP2C9 and VKORC1; vitamin K antagonists | **Search**: March 2014  **Included**: nine RCTs on AF/VTE; 2,812 adults  **Published**: 2005 to 2013 | Incidence of major bleeding, thrombosis and death |
| Goulding et al. (2014),^5^ CYP2C29, VKORC1, CYP4F2, HLA-B*5701, HIV anti-retroviral resistance mutations, TMPT, CYP2C19 and CYP3A5; warfarin | **Search**: January 1980 to December 2013  **Included**: 15 RCTs; eight on AF/VTE; 5,688 adults  **Published**: 2002 to 2013 | Percentage of TTR and adverse drug events |
| Jin et al. (2014),^6^ VKORC1-1639G>A; warfarin | **Search**: June 2013, updated in July 2013  **Included**: 32 prospective trials; 25 on AF/VTE; 5,005 adults  **Published**: 2005 to 2013 | Weighted mean maintenance dosage of warfarin |
| Shi et al. (2015),^7^ CYP2C9, VKORC1 and CYP4F2; warfarin | **Search**: March 2015  **Included**: 11 RCTs; eight on AF/VTE; 2,678 adults  **Published**: 2005 to 2013 | TTR |
| Sun et al. (2016),^8^ CYP4F2; warfarin | **Search**: August 2015  **Included**: 22 studies; 11 on AF/VTE; 4,549 adults  **Published**: 2009 to 2015 | Mean difference (MD) in daily warfarin dose (MDs represent the relative differences in the maintenance dose due to the normalisation procedure) |
| Tang et al. (2015),^9^ VKORC1, and CYP2C9; coumarin | **Search**: January 2000 to March 2014  **Included**: eight RCTs; seven on AF/VTE; 1,805 adults  **Published**: 2007 to 2013 | Mean difference in percentage of TTR |
| Xu et al. (2014),^10^ CYP2C9, VKORC1, and CYP4F2; warfarin | **Search**: January 2014  **Included**: eight RCTs; seven on AF/VTE; 2,098 adults  **Published**: 2005 to 2013 | TTR |
| Yu et al. (2016),^11^ Apolipoprotein E (ApoE); warfarin | **Search**: July 2015  **Included**: nine RCTs on AF/VTE; 1,766 adults  **Published**: 2005 to 2014 | The warfarin dose (mean and SD) associated with each genotype |

TTR = time in therapeutic range, INR = international normalised ratio, AF = atrial fibrillation, VTE = venous thromboembolism, RCT = randomised controlled trial, MD = mean difference, SD = standard deviation, ApoE = apolipoprotein E

**Patients with any condition requiring OAC; update searches**

| **Review authors (year), genes and OAC** | **Methods and study details** | **Primary outcomes** |
| --- | --- | --- |
| Asiimwe et al. (2020),^12^ CYP2C9, VKORC1, CYP4F2, CALU1, and NQO1; warfarin | **Search**: October 2018  **Included**: 48 studies, 2336 patients  **Published**: 2000 to 2019 | Warfarin dose change associated with each genotype |
| Kheiri et al. (2018),^13^ CYP2C9, CYP4F2, GGCX and VKORC1; VKAs | **Search**: October 2017  **Included**: 20 RCTs, 5980 patients  **Published**: 2005 to 2017 | TTR, bleeding (various), and mortality |
| Sridharan et al. (2021),^14^ CYP2C9, CYP4F2 and VKORC1; warfarin | **Search**: August 2020  **Included**: 26 studies, 7898 patients  **Published**: 2005 to 2020 | TTR, time to therapeutic or stable INR, bleeding, thromboembolism, over anticoagulation, mortality, dose required |
| Tian et al. (2021),^15^ NQO1; warfarin | **Search:** July 2021**  **Included:** 4 studies, 757 patients  **Published**: 2007 to 2020 | Warfarin dose change associated with each genotype |
| Tse et al. (2018),^16^ CYP2C9, CYP4F2 and VKORC1; warfarin | **Search**: October 2017  **Included**: 18 RCTs, 5230 patients  **Published**: 2005 to 2017 | Time to therapeutic or stable INR, TTR, bleeding, thromboembolism, mortality |
| Yang et al. (2019),^17^ CYP2C9, CYP4F2 and VKORC1; warfarin | **Search**: October 2017  **Included**: 15 RCTs, 4852 patients  **Published**: 2005 to 2017 | TTR, time to INR in range, bleeding, thromboembolism, mortality |

** Search date after publication submitted
TTR = time in therapeutic range, INR = international normalised ratio, AF = atrial fibrillation, RCT = randomised controlled trial, VKA = vitamin K antagonist, DOAC = direct oral anticoagulant

**Patients with any condition requiring OAC; unable to obtain full text**

| **Review authors (year), genes and OAC** | **Methods and study details** | **Primary outcomes** |
| --- | --- | --- |
| Xie et al. (2018),^18^ ABCB1; DOACs | **Search:** May 2018  **Included:** 10 studies, 2609 patients  **Published**: not in abstract | Plasma concentration, stroke, embolism, bleeding (various) |

1. Ng SS, Lai NM, Nathisuwan S, et al. Comparative efficacy and safety of warfarin care bundles and novel oral anticoagulants in patients with atrial fibrillation: a systematic review and network meta-analysis. *Scientific Reports* 2020;10:662. doi: <https://dx.doi.org/10.1038/s41598-019-57370-2>

2. Chen P, Sun Y-Q, Yang G-P, et al. Influence of the CYP4F2 polymorphism on the risk of hemorrhagic complications in coumarin-treated patients. *Saudi Med J* 2016;37(4):361-68. doi: 10.15537/smj.2016.4.14036

3. Dahal K, Sharma SP, Fung E, et al. Meta-analysis of randomized controlled trials of genotype-guided vs standard dosing of warfarin. *Chest* 2015;148(3):701-10. doi: 10.1378/chest.14-2947 [published Online First: 2015/03/26]

4. Franchini M, Mengoli C, Cruciani M, et al. Effects on bleeding complications of pharmacogenetic testing for initial dosing of vitamin K antagonists: a systematic review and meta-analysis. *J Thromb Haemost* 2014;12(9):1480-7. doi: 10.1111/jth.12647 [published Online First: 2014/07/22]

5. Goulding R, Dawes D, Price M, et al. Genotype-guided drug prescribing: a systematic review and meta-analysis of randomized control trials. *Br J Clin Pharmacol* 2015;80(4):868-77. doi: 10.1111/bcp.12475 [published Online First: 2015/07/22]

6. Jin B, Hong Y, Zhu J, et al. The impact of VKORC1-1639G > A genetic polymorphism upon warfarin dose requirement in different ethnic populations. *Curr Med Res Opin* 2014;30(8):1505-11. doi: 10.1185/03007995.2014.912982 [published Online First: 2014/04/09]

7. Shi C, Yan W, Wang G, et al. Pharmacogenetics-based versus conventional dosing of warfarin: a meta-analysis of randomized controlled trials. *PloS one* 2015;10(12):e0144511. doi: 10.1371/journal.pone.0144511 [published Online First: 2015/12/18]

8. Sun X, Yu WY, Ma WL, et al. Impact of the CYP4F2 gene polymorphisms on the warfarin maintenance dose: a systematic review and meta-analysis. *Biomed Rep* 2016;4(4):498-506. doi: 10.3892/br.2016.599 [published Online First: 2016/04/14]

9. Tang T, Liu J, Zuo K, et al. Genotype-guided dosing of coumarin anticoagulants: a meta-analysis of randomized controlled trials. *J Cardiovasc Pharmacol Ther* 2015;20(4):387-94. doi: 10.1177/1074248414565666 [published Online First: 2015/01/13]

10. Xu H, Xie X, Wang B, et al. Meta-analysis of efficacy and safety of genotype-guided pharmacogenetic dosing of warfarin. *Int J Cardiol* 2014;177(2):654-57. doi: 10.1016/j.ijcard.2014.09.174

11. Yu WY, Sun X, Wadelius M, et al. Influence of APOE gene polymorphism on interindividual and interethnic warfarin dosage requirement: a systematic review and meta-analysis. *Cardiovasc Ther* 2016;34(5):297-307. doi: 10.1111/1755-5922.12186 [published Online First: 2016/04/12]

12. Asiimwe IG, Zhang EJ, Osanlou R, et al. Genetic Factors Influencing Warfarin Dose in Black-African Patients: A Systematic Review and Meta-Analysis. *Clin Pharmacol Ther* 2020;107:1420-33. doi: <https://dx.doi.org/10.1002/cpt.1755>

13. Kheiri B, Abdalla A, Haykal T, et al. Meta-Analysis of Genotype-Guided Versus Standard Dosing of Vitamin K Antagonists. *Am J Cardiol* 2018;121:879-87. doi: <https://dx.doi.org/10.1016/j.amjcard.2017.12.023>

14. Sridharan K, Sivaramakrishnan G. A network meta-analysis of CYP2C9, CYP2C9 with VKORC1 and CYP2C9 with VKORC1 and CYP4F2 genotype-based warfarin dosing strategies compared to traditional. *J Clin Pharm Ther* 2021;46:640-48. doi: <https://dx.doi.org/10.1111/jcpt.13334>

15. Tian L, Xiao P, Zhou B, et al. Influence of NQO1 Polymorphisms on Warfarin Maintenance Dose: A Systematic Review and Meta-Analysis (rs1800566 and rs10517). *Cardiovascular therapeutics* 2021;2021:5534946. doi: <https://dx.doi.org/10.1155/2021/5534946>

16. Tse G, Gong M, Li G, et al. Genotype-guided warfarin dosing vs. conventional dosing strategies: a systematic review and meta-analysis of randomized controlled trials. *British journal of clinical pharmacology* 2018;84:1868-82. doi: <https://dx.doi.org/10.1111/bcp.13621>

17. Yang T, Zhou Y, Chen C, et al. Genotype-guided dosing versus conventional dosing of warfarin: A meta-analysis of 15 randomized controlled trials. *J Clin Pharm Ther* 2019;44:197-208. doi: <https://dx.doi.org/10.1111/jcpt.12782>

18. Xie Q, Xiang Q, Mu G, et al. Effect of ABCB1 Genotypes on the Pharmacokinetics and Clinical Outcomes of New Oral Anticoagulants: A Systematic Review and Meta-analysis. *Curr Pharm Des* 2018;24:3558-65. doi: <https://dx.doi.org/10.2174/1381612824666181018153641>
