# Supplementary material for: Oral anticoagulants: a systematic overview of reviews on efficacy and safety, genotyping, self-monitoring, and stakeholder experiences
Source: Syst Rev. 2022 Oct 28;11:232. doi: 10.1186/s13643-022-02098-w (PMC9615370; doi:10.1186/s13643-022-02098-w)
Supplement: Supplementary file 5 — Additional file 5. Potential efficacy and safety includes identified using title and abstract from the update searches. [file 13643_2022_2098_MOESM5_ESM.docx]

**Additional file 5: Potential efficacy and safety reviews identified using title and abstract from the update searches**

**Overviews**

In addition to the potentially included systematic reviews listed below, we found five potential systematic reviews of systematic reviews (overviews).^1-5^ Four were on efficacy and safety, and one^4^ was on genotyping, self-management, pharmacy management and DOACs versus warfarin for patients with AF only. Four overviews only included AF patients (one included only obese AF patients^5^); the fifth overview^3^ only included patients with pulmonary embolism (a VTE), and this found that DOACs were more effective and safer than heparin plus VKA. In one overview,^2^ the reviewers just looked at overlap and found very high overlap (24%) between efficacy and safety systematic reviews in AF patients; they found 66 systematic reviews that mainly relied on 18 RCTs.

1. Cope S, et al., Critical appraisal of network meta-analyses evaluating the efficacy and safety of new oral anticoagulants in atrial fibrillation stroke prevention trials. Value in Health, 2015. **18**: p. 234-49.

2. Doundoulakis I, et al., Overview of Systematic Reviews of Non-Vitamin K Oral Anticoagulants in Atrial Fibrillation. Circulation. Cardiovascular Quality & Outcomes, 2018. **11**: p. e004769.

3. Doundoulakis I, et al., Non-Vitamin K Antagonist Oral Anticoagulants in Pulmonary Embolism: An Overview of Systematic Reviews*.* Current Pharmaceutical Design, 2020. **26**: p. 2686-2691.

4. Ng SS, et al., Interventions and Strategies to Improve Oral Anticoagulant Use in Patients with Atrial Fibrillation: A Systematic Review of Systematic Reviews. Clinical Drug Investigation, 2018. **38**: p. 579-591.

5. Shaikh F, et al., Effectiveness of Direct Oral Anticoagulants in Obese Adults With Atrial Fibrillation: A Systematic Review of Systematic Reviews and Meta-Analysis. Frontiers in Cardiovascular Medicine, 2021. **8**: p. 732828.

**Articles identified n=468**

1. Abdool M, Kunutsor SK, Khunti K, et al. Does the presence of diabetes mellitus confer an increased risk of stroke in patients with atrial fibrillation on direct oral anticoagulants? A systematic review and meta-analysis. Diabetes & Metabolic Syndrome 2020;14:1725-33. doi: https://dx.doi.org/10.1016/j.dsx.2020.08.038

2. Abdulla A, Davis W, Ratnaweera N, et al. Case fatality rate of recurrent venous thromboembolism and major bleeding events among patients treated for cancer-associated venous thromboembolism: A systematic review. Blood Conference: 60th Annual Meeting of the American Society of Hematology, ASH 2018;132 doi: http://dx.doi.org/10.1182/blood-2018-99-110271

3. Abdulla A, Davis WM, Ratnaweera N, et al. A Meta-Analysis of Case Fatality Rates of Recurrent Venous Thromboembolism and Major Bleeding in Patients with Cancer. Thrombosis and haemostasis 2020;120(4):702-13. doi: http://dx.doi.org/10.1055/s-0040-1708481

4. Abdullah HM, Ullah W, Jafar M, et al. Apixaban for Stroke Prevention in End-Stage Kidney Disease Patients with Atrial Fibrillation: A Meta-Analysis. J Am Coll Cardiol 2020;75(11):514. doi: http://dx.doi.org/10.1016/S0735-1097%2820%2931141-4

5. Abdullah HM, Ullah W, Jafar MS, et al. Safety and Efficacy of Apixaban, Rivaroxaban, and Warfarin in End-Stage Renal Disease With Atrial Fibrillation: A Systematic Review and Meta-Analysis. Cardiovascular Revascularization Medicine 2021;30:26-32. doi: https://dx.doi.org/10.1016/j.carrev.2020.09.041

6. Abildgaard A, Madsen SA, Hvas AM. Dosage of Anticoagulants in Obesity: Recommendations Based on a Systematic Review. Semin Thromb Hemost 2020;46:932-69. doi: https://dx.doi.org/10.1055/s-0040-1718405

7. Acanfora D, Ciccone MM, Carlomagno V, et al. A Systematic Review of the Efficacy and Safety of Direct Oral Anticoagulants in Atrial Fibrillation Patients with Diabetes Using a Risk Index. Journal of Clinical Medicine 2021;10:29. doi: https://dx.doi.org/10.3390/jcm10132924

8. Acanfora D, Ciccone MM, Scicchitano P, et al. Efficacy and Safety of Direct Oral Anticoagulants in Patients With Atrial Fibrillation and High Thromboembolic Risk. A Systematic Review. Frontiers in Pharmacology 2019;10:1048. doi: https://dx.doi.org/10.3389/fphar.2019.01048

9. Ahmed A, Ahmed R, Ali SS, et al. Intracerebral hemorrhage outcomes in patients using direct oral anticoagulants versus vitamin K antagonists: a meta-analysis. Clinical Neurology & Neurosurgery 2020;198:106146. doi: https://dx.doi.org/10.1016/j.clineuro.2020.106146

10. Aibar J, Schulman S. Arterial Thrombosis in Patients with Antiphospholipid Syndrome: A Review and Meta-Analysis. Semin Thromb Hemost 2021;47:709-23. doi: https://dx.doi.org/10.1055/s-0041-1725057

11. Aijaz T, Obi ON, Khokhar N, et al. Safety and Efficacy of Direct Oral Anticoagulants in Morbidly Obese Patients: A Meta-Analysis. Blood 2019;134(Supplement 1):1166. doi: http://dx.doi.org/10.1182/blood-2019-125461

12. Al Mukdad M, Al-Badriyeh D, Elewa HF. Cost-effectiveness Evaluations Among the Direct Oral Anticoagulants for the Prevention and Treatment of Venous Thromboembolism: Systematic Review. Clinical & Applied Thrombosis/Hemostasis 2019;25:1076029619849103. doi: https://dx.doi.org/10.1177/1076029619849103

13. Al Said S, Alabed S, Kaier K, et al. Non-vitamin K antagonist oral anticoagulants (NOACs) post-percutaneous coronary intervention: a network meta-analysis. Cochrane Database of Systematic Reviews 2019;12:CD013252. doi: https://dx.doi.org/10.1002/14651858.CD013252.pub2

14. Al-Abcha A, Herzallah K, Saleh Y, et al. The Role of Direct Oral Anticoagulants Versus Vitamin K Antagonists in the Treatment of Left Ventricular Thrombi: A Meta-Analysis and Systematic Review. Am J Cardiol 2021;21:435-41. doi: https://dx.doi.org/10.1007/s40256-020-00458-2

15. Alhousani M, Malik SU, Abu-Hashyeh A, et al. Using oral anticoagulants among chronic kidney disease patients to prevent recurrent venous thromboembolism: A systematic review and meta-analysis. Thromb Res 2021;198:103-14. doi: https://dx.doi.org/10.1016/j.thromres.2020.11.036

16. Almelor-Sembrana LG, Gloria MA, Alejandria MM. Novel oral anticoagulants plus antiplatelet therapy vs. vitamin K antagonists plus antiplatelet therapy in atrial fibrillation patients who underwent percutaneous coronary intervention:a meta-analysis. Eur Heart J 2019;40(Supplement 1):2975. doi: http://dx.doi.org/10.1093/eurheartj/ehz745.1147

17. Aloysius MM, Perisetti A, Goyal H, et al. Direct-acting oral anticoagulants versus warfarin in relation to risk of gastrointestinal bleeding: a systematic review and meta-analysis of randomized controlled trials. Annals of Gastroenterology 2021;34:651-59. doi: https://dx.doi.org/10.20524/aog.2021.0658

18. Alwafi H, Alotaibi B, Naser AY, et al. The safety and efficacy of the use of oral anticoagulant medications in patients with diabetes mellitus: A systematic review. Saudi Pharmaceutical Journal 2021 doi: http://dx.doi.org/10.1016/j.jsps.2021.11.001

19. Anghel L, Sascau R, Trifan A, et al. Non-Vitamin K Antagonist Oral Anticoagulants and the Gastrointestinal Bleeding Risk in Real-World Studies. Journal of Clinical Medicine 2020;9:09. doi: https://dx.doi.org/10.3390/jcm9051398

20. Anonymous. Corrigendum to: Reduced-dose direct oral anticoagulants in the extended treatment of venous thromboembolism: a systematic review and meta-analysis (Journal of Thrombosis and Haemostasis, (2018), 16, 7, (1288-1295), 10.1111/jth.14156). Journal of Thrombosis and Haemostasis 2021;19(9):2365-66. doi: http://dx.doi.org/10.1111/jth.15446

21. Antza C, Doundoulakis I, Akrivos E, et al. Non-vitamin K oral anticoagulants in nonvalvular atrial fibrillation: a network meta-analysis. Scand Cardiovasc J 2019;53:48-54. doi: https://dx.doi.org/10.1080/14017431.2019.1594353

22. Aronow WS, Shamliyan TA. Comparative Effectiveness and Safety of Rivaroxaban in Adults With Nonvalvular Atrial Fibrillation. Am J Ther 2019;26:e679-e703. doi: https://dx.doi.org/10.1097/MJT.0000000000000890

23. Aronow WS, Shamliyan TA. Comparative Clinical Outcomes of Edoxaban in Adults With Nonvalvular Atrial Fibrillation. Am J Ther 2020;27:e270-e85. doi: https://dx.doi.org/10.1097/MJT.0000000000000848

24. Aryal MR, Gosain R, Donato A, et al. Systematic review and meta-analysis of the efficacy and safety of apixaban compared to rivaroxaban in acute VTE in the real world. Blood Advances 2019;3:2381-87. doi: https://dx.doi.org/10.1182/bloodadvances.2019000572

25. Ashley J, Sood MM. Novel oral anticoagulants in chronic kidney disease: ready for prime time? Current Opinion in Nephrology & Hypertension 2018;27:201-08. doi: https://dx.doi.org/10.1097/MNH.0000000000000410

26. Avendano R, Romero J, Lupercio F, et al. Clinical outcomes in patients with atrial fibrillation receiving amiodarone on NOACs vs. warfarin. J Interv Card Electrophysiol 2019;54:73-80. doi: https://dx.doi.org/10.1007/s10840-018-0427-y

27. Bawazeer GA, Alkofide HA, Alsharafi AA, et al. Interrupted versus uninterrupted anticoagulation therapy for catheter ablation in adults with arrhythmias. Cochrane Database of Systematic Reviews 2021;2021(10) (no pagination) doi: http://dx.doi.org/10.1002/14651858.CD013504.pub2

28. Becattini C, Cimini LA. Long term use of anticoagulant therapy for patients with pulmonary embolism. Expert Review of Hematology 2020;13:709-18. doi: https://dx.doi.org/10.1080/17474086.2020.1770589

29. Bennaghmouch N, de Veer A, Bode K, et al. Efficacy and Safety of the Use of Non-Vitamin K Antagonist Oral Anticoagulants in Patients With Nonvalvular Atrial Fibrillation and Concomitant Aspirin Therapy: A Meta-Analysis of Randomized Trials. Circulation 2018;137:1117-29. doi: https://dx.doi.org/10.1161/CIRCULATIONAHA.117.028513

30. Bhatt NR, Davis NF, Nolan WJ, et al. Incidence of Visible Hematuria Among Antithrombotic Agents: A Systematic Review of Over 175,000 Patients. Urology 2018;114:27-32. doi: https://dx.doi.org/10.1016/j.urology.2017.11.023

31. Bhogal S, Mawa K, Bhandari T, et al. Use of NOACs Versus Vitamin K Antagonist in Atrial Fibrillation Catheter Ablation: An Updated Meta-analysis With Subgroup Analysis. Am J Ther 2021;18:18. doi: https://dx.doi.org/10.1097/MJT.0000000000001391

32. Bixby AL, Lichvar AB, Salerno D, et al. Use of direct-acting oral anticoagulants in solid organ transplantation: A systematic review. Pharmacotherapy:The Journal of Human Pharmacology & Drug Therapy 2021;41:28-43. doi: https://dx.doi.org/10.1002/phar.2485

33. Blandino A, Bianchi F, Biondi-Zoccai G, et al. Apixaban for periprocedural anticoagulation during catheter ablation of atrial fibrillation: A systematic reviewand meta-analysis. Europace 2016;18(Supplement 1):i62. doi: http://dx.doi.org/10.1093/europace/euw158

34. Bonanad C, Garcia-Blas S, Torres L, et al. Direct Oral Anticoagulants versus Warfarin in Octogenarians with Nonvalvular Atrial Fibrillation: A Systematic Review and Meta-Analysis. Journal of Clinical Medicine 2021;10:12. doi: https://dx.doi.org/10.3390/jcm10225268

35. Bose G, Graveline J, Yogendrakumar V, et al. Direct oral anticoagulants in treatment of cerebral venous thrombosis: a systematic review. BMJ Open 2021;11:e040212. doi: https://dx.doi.org/10.1136/bmjopen-2020-040212

36. Brandao GMS, Candido RCF, Rollo HA, et al. Direct oral anticoagulants for treatment of deep vein thrombosis: overview of systematic reviews. Jornal Vascular Brasileiro 2018;17:310-17. doi: https://dx.doi.org/10.1590/1677-5449.005518

37. Brandao GMS, Malgor RD, Vieceli T, et al. A network meta-analysis of direct factor Xa inhibitors for the treatment of cancer-associated venous thromboembolism. Vascular 2021:17085381211002726. doi: https://dx.doi.org/10.1177/17085381211002726

38. Briceno DF, Spinetto PV, Cyrille N, et al. Left atrial appendage occlusion device and novel oral anticoagulants versus warfarin for stroke prevention in non-valvular atrial fibrillation: A systematic review and meta-analysis of randomized control trials. J Am Coll Cardiol 2015;1):A304.

39. Briere JB, Bowrin K, Coleman C, et al. Real-world clinical evidence on rivaroxaban, dabigatran, and apixaban compared with vitamin K antagonists in patients with nonvalvular atrial fibrillation: a systematic literature review. Expert Review of Pharmacoeconomics & Outcomes Research 2019;19:27-36. doi: https://dx.doi.org/10.1080/14737167.2018.1518134

40. Briere JB, Bowrin K, Millier A, et al. Number needed to treat based on real-world evidence for non-vitamin K antagonist oral anticoagulants versus vitamin K antagonist oral anticoagulants in stroke prevention in patients with non-valvular atrial fibrillation. J Med Econ 2019;22:760-65. doi: https://dx.doi.org/10.1080/13696998.2019.1606001

41. Briere JB, Wu O, Bowrin K, et al. Impact of methodological choices on a meta-analysis of real-world evidence comparing non-vitamin-K antagonist oral anticoagulants with vitamin K antagonists for the treatment of patients with non-valvular atrial fibrillation. Curr Med Res Opin 2019;35:1867-72. doi: https://dx.doi.org/10.1080/03007995.2019.1647020

42. Brockmeyer M, Lin Y, Parco C, et al. Uninterrupted direct oral anticoagulants and vitamin K antagonists during ablation for atrial fibrillation: An updated meta-analysis. Eur Heart J 2020;41(SUPPL 2):569. doi: http://dx.doi.org/10.1093/ehjci/ehaa946.0569

43. Brockmeyer M, Lin Y, Parco C, et al. Uninterrupted anticoagulation during catheter ablation for atrial fibrillation: no difference in major bleeding and stroke between direct oral anticoagulants and vitamin K antagonists in an updated meta-analysis of randomised controlled trials. Acta Cardiol 2021;76:288-95. doi: https://dx.doi.org/10.1080/00015385.2020.1724689

44. Brokmeier H, Kido K. Off-label Use for Direct Oral Anticoagulants: Valvular Atrial Fibrillation, Heart Failure, Left Ventricular Thrombus, Superficial Vein Thrombosis, Pulmonary Hypertension-a Systematic Review. Ann Pharmacother 2021;55:995-1009. doi: https://dx.doi.org/10.1177/1060028020970618

45. Bruins S, K M, Berge E. Factor Xa inhibitors versus vitamin K antagonists for preventing cerebral or systemic embolism in patients with atrial fibrillation. Cochrane Database of Systematic Reviews 2018;3:CD008980. doi: https://dx.doi.org/10.1002/14651858.CD008980.pub3

46. Brunetti ND, Tarantino N, De Gennaro L, et al. Direct oral anticoagulants versus standard triple therapy in atrial fibrillation and PCI: meta-analysis. Open Heart 2018;5:e000785. doi: https://dx.doi.org/10.1136/openhrt-2018-000785

47. Brunetti ND, Tarantino N, De Gennaro L, et al. Direct oral anti-coagulants compared to vitamin-K antagonists in cardioversion of atrial fibrillation: an updated meta-analysis. Journal of Thrombosis & Thrombolysis 2018;45:550-56. doi: https://dx.doi.org/10.1007/s11239-018-1622-5

48. Brunetti ND, Tricarico L, De Gennaro L, et al. Meta-analysis study on direct oral anticoagulants vs warfarin therapy in atrial fibrillation and PCI: Dual or triple approach? International Journal of Cardiology Heart & Vasculature 2020;29:100569. doi: https://dx.doi.org/10.1016/j.ijcha.2020.100569

49. Brunetti ND, Tricarico L, Tilz RR, et al. Lower Major Bleeding Rates with Direct Oral Anticoagulants in Catheter Ablation of Atrial Fibrillation: an Updated Meta-analysis of Randomized Controlled Studies. Cardiovascular Drugs & Therapy 2020;34:209-14. doi: https://dx.doi.org/10.1007/s10557-020-06947-5

50. Buck MM, Haddon AM, Paneccasio A, et al. Safety and Efficacy of Rivaroxaban and Apixaban in Patients with Increased Body Mass: a Systematic Review. Clin Drug Investig 2021;41:353-69. doi: https://dx.doi.org/10.1007/s40261-021-01019-4

51. Burmeister C, Beran A, Mhanna M, et al. Efficacy and Safety of Direct Oral Anticoagulants Versus Vitamin K Antagonists in the Treatment of Left Ventricular Thrombus: A Systematic Review and Meta-analysis. Am J Ther 2021;28:e411-e19. doi: https://dx.doi.org/10.1097/MJT.0000000000001351

52. Burr N, Lummis K, Corp A, et al. The risk of gastrointestinal bleeding with new novel non-vitamin K Antagonist, oral anticoagulant medications. Systematic review and network meta-analysis. Gastroenterology 2016;1):S835.

53. Caldeira D, Barra M, Ferreira A, et al. Risk of major gastrointestinal bleeding with non-vitamin K antagonist oral anticoagulants: Systematic review with meta-analysis. Rev Port Cardiol 2016;35(1 Supplement 1):60.

54. Caldeira D, Nunes-Ferreira A, Rodrigues R, et al. Non-vitamin K antagonist oral anticoagulants in elderly patients with atrial fibrillation: A systematic review with meta-analysis and trial sequential analysis. Arch Gerontol Geriatr 2019;81:209-14. doi: https://dx.doi.org/10.1016/j.archger.2018.12.013

55. Camilli M, Lombardi M, Del Buono MG, et al. Direct oral anticoagulants vs. vitamin K antagonists for the treatment of left ventricular thrombosis: a systematic review of the literature and meta-analysis. European Heart Journal Cardiovascular Pharmacotherapy 2021;7:e21-e25. doi: https://dx.doi.org/10.1093/ehjcvp/pvaa134

56. Camilli M, Lombardi M, Giuseppe M, et al. Direct oral anticoagulants vs. vitamin K antagonists for left ventricular thrombosis: A meta-analysis. European Heart Journal, Supplement 2020;22(SUPPL N):N26-N27. doi: http://dx.doi.org/10.1093/eurheartj/suaa194

57. Cao B, Yao X, Zhang L, et al. Efficacy and Safety of Direct Oral Anticoagulants in Patients with Diabetes and Nonvalvular Atrial Fibrillation: Meta-Analysis of Observational Studies. Cardiovascular therapeutics 2021;2021:5520027. doi: https://dx.doi.org/10.1155/2021/5520027

58. Caputo R, Pyle J, Kuriakose P, et al. A systematic review of apixaban in prevention and treatment of cancer-associated venous thromboembolism. J Am Pharm Assoc 2021;61:e26-e38. doi: https://dx.doi.org/10.1016/j.japh.2021.06.005

59. Carmo J, Costa FM, Ferreira J, et al. Dabigatran in the real world of atrial fibrillation: Meta-analysis of observational comparison studies with vitamin K antagonists. Eur Heart J 2016;37(Supplement 1):504. doi: http://dx.doi.org/10.1093/eurheartj/ehw432

60. Casula M, Fabris F, Leonardi S, et al. Efficacy and safety of direct oral anticoagulants vs. vitamin K antagonists in subgroups of patients with atrial fibrillation undergoing percutaneous coronary intervention. European Heart Journal, Supplement 2019;21(SUPPL J):J35-J36. doi: http://dx.doi.org/10.1093/eurheartj/suz247

61. Casula M, Fabris F, Leonardi S, et al. Efficacy and safety of direct oral anticoagulants versus vitamin K antagonists in subgroups of patients with atrial fibrillation undergoing percutaneous coronary intervention. G Ital Cardiol 2019;20(12 Supplement 1):89S.

62. Casula M, Fortuni F, Fabris F, et al. Efficacy and safety of direct Xa oral inhibitors versus warfarin in patients with atrial fibrillation and cancer: A meta-analysis of randomized controlled trials. Europace 2020;22(SUPPL 1):i209.

63. Casula M, Fortuni F, Fabris F, et al. Direct oral Xa inhibitors versus warfarin in patients with cancer and atrial fibrillation: a meta-analysis. Journal of Cardiovascular Medicine 2020;21:570-76. doi: https://dx.doi.org/10.2459/JCM.0000000000001041

64. Cavallari I, Verolino G, Patti G. Efficacy and safety of non-vitamin K oral anticoagulants in patients with atrial fibrillation and cancer. Eur Heart J 2019;40(Supplement 1):1849. doi: http://dx.doi.org/10.1093/eurheartj/ehz745.0021

65. Cen Z, Meng Q, Cui K. New oral anticoagulants for nonvalvular atrial fibrillation with stable coronary artery disease: A meta-analysis. Pacing & Clinical Electrophysiology 2020;43:1393-400. doi: https://dx.doi.org/10.1111/pace.14081

66. Cerda P, Becattini C, Iriarte A, et al. Direct oral anticoagulants versus vitamin K antagonists in antiphospholipid syndrome: A meta-analysis. European journal of internal medicine 2020;79:43-50. doi: https://dx.doi.org/10.1016/j.ejim.2020.05.012

67. Cerrud-Rodriguez RC, Romero J, Diaz JC, et al. New evidence of significant incremental benefit of uninterrupted direct oral anticoagulants vs. uninterrupted vitamin K antagonists during catheter ablation of atrial fibrillation: A systematic review and meta-analysis of randomized controlled trials. Circulation Conference: American Heart Association Scientific Sessions, AHA 2019;140 doi: http://dx.doi.org/10.1161/circ.140.suppl_1.9566

68. Cerrud-Rodriguez RC, Romero J, Diaz JC, et al. Uninterrupted direct oral anticoagulants vs. uninterrupted vitamin k antagonists during catheter ablation of non-valvular atrial fibrillation: A systematic review and meta-analysis. Circulation Conference 2018;138

69. Cerrud-Rodríguez Roberto C, Romero J, Diaz Juan C, et al. Abstract 10363: Uninterrupted Direct Oral Anticoagulants vs. Uninterrupted Vitamin K Antagonists During Catheter Ablation of Non-Valvular Atrial Fibrillation: A Systematic Review and Meta-Analysis. Circulation 2018;138:A10363-A63.

70. Chan YH, Lee HF, Chao TF, et al. Real-world Comparisons of Direct Oral Anticoagulants for Stroke Prevention in Asian Patients with Non-valvular Atrial Fibrillation: a Systematic Review and Meta-analysis. Cardiovascular Drugs & Therapy 2019;33:701-10. doi: https://dx.doi.org/10.1007/s10557-019-06910-z

71. Chaudhary R, Bliden K, Tantry U, et al. Safety and efficacy of direct oral anticoagulants in elderly patients undergoing treatment for venous thromboembolism: Systemic review and network meta-analysis. Eur Heart J 2017;38(Supplement 1):283. doi: http://dx.doi.org/10.1093/eurheartj/ehx502.P1416

72. Chaudhary R, Bliden KP, Tantry US, et al. Safety and efficacy of direct oral anticoagulants in obese patients undergoing treatment for venous thromboembolism: Systemic review and network meta-analysis. Eur Heart J 2017;38(Supplement 1):669. doi: http://dx.doi.org/10.1093/eurheartj/ehx502.P3282

73. Chaudhary R, Pagali S, Garg J, et al. DOACs Versus VKAs in Older Adults Treated for Acute Venous Thromboembolism: Systematic Review and Meta-Analysis. Journal of the American Geriatrics Society 2020;68:2021-26. doi: https://dx.doi.org/10.1111/jgs.16549

74. Chen C, Cao Y, Zheng Y, et al. Effect of Rivaroxaban or Apixaban in Atrial Fibrillation Patients with Stage 4-5 Chronic Kidney Disease or on Dialysis. Cardiovascular Drugs & Therapy 2021;35:273-81. doi: https://dx.doi.org/10.1007/s10557-021-07144-8

75. Chen F, Zhou Y, Wan Q, et al. Effect of non-vitamin K antagonist oral anticoagulants versus warfarin in heart failure patients with atrial fibrillation. Heart Failure Reviews 2021;26:1391-97. doi: https://dx.doi.org/10.1007/s10741-020-09946-8

76. Chen HB, Xiu J, Li YH, et al. The risk of bleeding and all-cause mortality with edoxaban versus vitamin K antagonists: A meta-analysis of phase III randomized controlled trials. Thromb Res 2020;194:82-90. doi: https://dx.doi.org/10.1016/j.thromres.2020.06.009

77. Chen HY, Ou SH, Huang CW, et al. Efficacy and Safety of Direct Oral Anticoagulants vs Warfarin in Patients with Chronic Kidney Disease and Dialysis Patients: A Systematic Review and Meta-Analysis. Clin Drug Investig 2021;41:341-51. doi: https://dx.doi.org/10.1007/s40261-021-01016-7

78. Chen R, Zhou J, Liu C, et al. Direct oral anticoagulants versus vitamin K antagonists for patients with left ventricular thrombus: a systematic review and meta-analysis. Polish Archives Of Internal Medicine 2021;131:429-38. doi: https://dx.doi.org/10.20452/pamw.15923

79. Chen Y, Mao M, Chang J, et al. Safety and efficacy of new oral anticoagulants compared to those of warfarin in AF patients with cancer: a meta-analysis of randomized clinical trials and observational studies. Eur J Clin Pharmacol 2021;77:849-57. doi: https://dx.doi.org/10.1007/s00228-021-03132-x

80. Chen Y, Zhao Y, Dang G, et al. Stroke event rates and the optimal antithrombotic choice of patients with paroxysmal atrial fibrillation: A systematic review and meta-analysis of randomized controlled trials. Medicine (United States) 2015;94(52) (no pagination) doi: http://dx.doi.org/10.1097/MD.0000000000002364

81. Cheng W, Liu W, Li B, et al. Relationship of Anticoagulant Therapy With Cognitive Impairment Among Patients With Atrial Fibrillation: A Meta-Analysis and Systematic Review. J Cardiovasc Pharmacol 2018;71:380-87. doi: https://dx.doi.org/10.1097/FJC.0000000000000575

82. Cheung CYS, Parikh J, Farrell A, et al. Direct Oral Anticoagulant Use in Chronic Kidney Disease and Dialysis Patients With Venous Thromboembolism: A Systematic Review of Thrombosis and Bleeding Outcomes. Ann Pharmacother 2021;55:711-22. doi: https://dx.doi.org/10.1177/1060028020967635

83. Chiorescu RM, Mocan M, Stoia MA, et al. Arguments for Using Direct Oral Anticoagulants in Cancer-Related Venous Thromboembolism. Healthcare 2021;9:28. doi: https://dx.doi.org/10.3390/healthcare9101287

84. Choi J, No JE, Lee JY, et al. Efficacy and Safety of Clinically Driven Low-Dose Treatment with Direct Oral Anticoagulants in Asians with Atrial Fibrillation: a Systematic Review and Meta-analysis. Cardiovascular Drugs & Therapy 2021;16:16. doi: https://dx.doi.org/10.1007/s10557-021-07171-5

85. Chokesuwattanaskul R, Thongprayoon C, Bathini T, et al. Efficacy and safety of anticoagulation for atrial fibrillation in patients with cirrhosis: A systematic review and meta-analysis. Dig Liver Dis 2019;51:489-95. doi: https://dx.doi.org/10.1016/j.dld.2018.12.001

86. Chokesuwattanaskul R, Thongprayoon C, Tanawuttiwat T, et al. Safety and efficacy of apixaban versus warfarin in patients with end-stage renal disease: Meta-analysis. Pacing & Clinical Electrophysiology 2018;41:627-34. doi: https://dx.doi.org/10.1111/pace.13331

87. Chokesuwattanaskul R, Thongprayoon C, Tanawuttiwat T, et al. Safety and efficacy of apixaban versus warfarin in patients with end-stage renal disease: Meta-analysis. Pacing and clinical electrophysiology : PACE 2018;41(7):878. doi: http://dx.doi.org/10.1111/pace.13390

88. Chopard R, Albertsen IE, Piazza G. Diagnosis and Treatment of Lower Extremity Venous Thromboembolism: A Review. JAMA 2020;324:1765-76. doi: https://dx.doi.org/10.1001/jama.2020.17272

89. Chopra R, Kohli V, Jim B. The Safety and Efficacy of Apixaban Versus Warfarin in Chronic Kidney Disease Patients with Atrial Fibrillation: A Meta-Analysis. Am J Kidney Dis 2019;73(5):664-65. doi: http://dx.doi.org/10.1053/j.ajkd.2019.03.090

90. Ciccone MM, Bellino MC, Cecere A, et al. Efficacy and safety of direct oral anticoagulants in patients with atrial fibrillation and high thromboembolic risk. A systematic review. G Ital Cardiol 2019;20(12 Supplement 1):161S.

91. Cohen AT, Berger SE, Milenkovic D, et al. Anticoagulant selection for patients with VTE-Evidence from a systematic literature review of network meta-analyses. Pharmacol Res 2019;143:166-77. doi: https://dx.doi.org/10.1016/j.phrs.2019.03.017

92. Cohen AT, Hill NR, Luo X, et al. A systematic review of network meta-analyses among patients with nonvalvular atrial fibrillation: A comparison of efficacy and safety following treatment with direct oral anticoagulants. Int J Cardiol 2018;269:174-81. doi: https://dx.doi.org/10.1016/j.ijcard.2018.06.114

93. Coleman CI, Briere JB, Fauchier L, et al. Meta-analysis of real-world evidence comparing non-vitamin K antagonist oral anticoagulants with vitamin K antagonists for the treatment of patients with non-valvular atrial fibrillation. Journal of Market Access & Health Policy 2019;7:1574541. doi: https://dx.doi.org/10.1080/20016689.2019.1574541

94. Coleman CI, Sasiela K, Smith E, et al. Comparative efficacy and safety of anticoagulation for the treatment of cancer-associated thrombosis: A systematic review and meta-analysis of randomized controlled trials. Research and Practice in Thrombosis and Haemostasis 2018;2(Supplement 1):248. doi: http://dx.doi.org/10.1002/rth2.12125

95. Cordero A, Ferreiro JL, Bertomeu-Gonzalez V, et al. Direct Oral Anticoagulants Versus Vitamin-K Antagonist After PCIs in Patients With AF: A Meta-analysis of Cardiac Ischemic Events. J Cardiovasc Pharmacol 2021;77:164-69. doi: https://dx.doi.org/10.1097/FJC.0000000000000938

96. Costello M, Murphy R, Judge C, et al. Effect of non-vitamin-K oral anticoagulants on stroke severity compared to warfarin: a meta-analysis of randomized controlled trials. Eur J Neurol 2020;27:413-18. doi: https://dx.doi.org/10.1111/ene.14134

97. Daei M, Khalili H, Heidari Z. Direct oral anticoagulant safety during breastfeeding: a narrative review. Eur J Clin Pharmacol 2021;77:1465-71. doi: https://dx.doi.org/10.1007/s00228-021-03154-5

98. Dai Q, Deng X, Zhou L, et al. Real-world use of nonvitamin K antagonist oral anticoagulant in atrial fibrillation patients with liver disease: A meta-analysis. Clin Cardiol 2020;43:676-83. doi: https://dx.doi.org/10.1002/clc.23408

99. Dalia T, Lahan S, Ranka S, et al. Warfarin versus direct oral anticoagulants for treating left ventricular thrombus: a systematic review and meta-analysis. Thrombosis Journal [Electronic Resource] 2021;19:7. doi: https://dx.doi.org/10.1186/s12959-021-00259-w

100. de Vries TAC, Hirsh J, Xu K, et al. Apixaban for Stroke Prevention in Atrial Fibrillation: Why are Event Rates Higher in Clinical Practice than in Randomized Trials?-A Systematic Review. Thromb Haemost 2020;120:1323-29. doi: https://dx.doi.org/10.1055/s-0040-1713889

101. Deitelzweig S, Cichewicz A, DiFusco M, et al. Pcv14 an Appraisal of Real-World Evidence (Rwe) in Patients with Non-Valvular Atrial Fibrillation (Nvaf) Treated with Non-Vitamin K Antagonist Oral Anticoagulants (Noacs). Value Health 2019;22(Supplement 2):S120. doi: http://dx.doi.org/10.1016/j.jval.2019.04.450

102. Deitelzweig S, Farmer C, Luo X, et al. Comparison of major bleeding risk in patients with non-valvular atrial fibrillation receiving direct oral anticoagulants in the real-world setting: a network meta-analysis. Curr Med Res Opin 2018;34:487-98. doi: https://dx.doi.org/10.1080/03007995.2017.1411793

103. Deitelzweig SB, Farmer C, Luo X, et al. Real-world comparison of major bleeding risk associated with direct oral anticoagulants or warfarin in patients with non-valvular atrial fibrillation: A systematic review and network meta-analysis. Eur Heart J 2017;38(Supplement 1):577-78. doi: http://dx.doi.org/10.1093/eurheartj/ehx502.P2691

104. Deng K, Cheng J, Rao S, et al. Efficacy and Safety of Direct Oral Anticoagulants in Elderly Patients With Atrial Fibrillation: A Network Meta-Analysis. Frontiers in Medicine 2020;7:107. doi: https://dx.doi.org/10.3389/fmed.2020.00107

105. Deng Y, Tong Y, Deng Y, et al. Non-Vitamin K Antagonist Oral Anticoagulants Versus Warfarin in Patients With Cancer and Atrial Fibrillation: A Systematic Review and Meta-Analysis. Journal of the American Heart Association 2019;8:e012540. doi: https://dx.doi.org/10.1161/JAHA.119.012540

106. Dentali F, Sironi AP, Gianni M, et al. Gender difference in efficacy and safety of direct oral anticoagulants in patients with non-valvular atrial fibrillation or venous thromboembolism. A systematic review and a meta-analysis of the literature. Thromb Res 2014;2):S88.

107. Desai R, Koipallil GK, Thomas N, et al. Efficacy and safety of direct oral anticoagulants for secondary prevention of cancer associated thrombosis: a meta-analysis of randomized controlled trials. Scientific Reports 2020;10:18945. doi: https://dx.doi.org/10.1038/s41598-020-75863-3

108. Dewar FA, Musgrave KM, Simpson J, et al. The role of primary thromboprophylaxis in people with cancer: A systematic review and meta-analysis. Research and Practice in Thrombosis and Haemostasis Conference 2021;5 doi: http://dx.doi.org/10.1002/rth2.12589

109. Dhanda S, Osborne V, Shakir S. The association between direct Oral anticoagulants and gynecological bleeding in women v. standard therapy: A systematic review. Pharmacoepidemiol Drug Saf 2019;28(Supplement 2):565. doi: http://dx.doi.org/10.1002/pds.4864

110. Di Monaco A, Guida P, Vitulano N, et al. Catheter ablation of atrial fibrillation with uninterrupted anticoagulation: a meta-analysis of six randomized controlled trials. Journal of Cardiovascular Medicine 2020;21:483-90. doi: https://dx.doi.org/10.2459/JCM.0000000000000939

111. DiRisio AC, Harary M, Muskens IS, et al. Outcomes of intraparenchymal hemorrhage after direct oral anticoagulant or vitamin K antagonist therapy: A systematic review and meta-analysis. Journal of Clinical Neuroscience 2019;62:188-94. doi: https://dx.doi.org/10.1016/j.jocn.2018.11.032

112. Djulbegovic M, Lee A. Direct oral anticoagulants for the prevention of recurrent venous thromboembolism: A systematic review & network meta-analysis. Blood Conference: 59th Annual Meeting of the American Society of Hematology, ASH 2017;130

113. Djulbegovic M, Lee AI, Chen K. Which patients with unprovoked venous thromboembolism should receive extended anticoagulation with direct oral anticoagulants? A systematic review, network meta-analysis, and decision analysis. J Eval Clin Pract 2020;26:7-17. doi: https://dx.doi.org/10.1111/jep.13194

114. Do C, Costa FM, Ferreira J, et al. Dabigatran in the real world of atrial fibrillation: Systematic review and meta-analysis of observational comparison studies with warfarin. Rev Port Cardiol 2016;35(1 Supplement 1):119.

115. Dong S, Zhang Y, Li Y, et al. Direct Oral Anticoagulant for the Treatment of VTE in Cancer Patients: A Systematic Review and Meta-analysis. Ann Pharmacother 2021;55:430-39. doi: https://dx.doi.org/10.1177/1060028020960037

116. Dong Y, Wang Y, Ma RL, et al. Efficacy and safety of direct oral anticoagulants versus low-molecular-weight heparin in patients with cancer: a systematic review and meta-analysis. Journal of Thrombosis & Thrombolysis 2019;48:400-12. doi: https://dx.doi.org/10.1007/s11239-019-01871-4

117. Dorreen AP, Miller CS, Barkun AN, et al. Non-Vitamin K Antagonist Oral Anticoagulants and Gastrointestinal Bleeding: A Network Meta-Analysis. Gastroenterology 2018;154(6 Supplement 1):S-784. doi: http://dx.doi.org/10.1016/S0016-5085%2818%2932702-1

118. Douros A, Durand M, Doyle CM, et al. Comparative Effectiveness and Safety of Direct Oral Anticoagulants in Patients with Atrial Fibrillation: A Systematic Review and Meta-Analysis of Observational Studies. Drug Saf 2019;42:1135-48. doi: https://dx.doi.org/10.1007/s40264-019-00842-1

119. Drogkaris S, Thomopoulos C, Kalos T, et al. Net clinical benefit of direct oral anticoagulants in atrial fibrillation patients with or without diabetes mellitus: A meta-analysis of outcome trials. Diabetes Res Clin Pract 2021;182:109147. doi: https://dx.doi.org/10.1016/j.diabres.2021.109147

120. Dufrost V, Reshetnyak T, Satybaldyeva M, et al. Increased risk of thrombosis in antiphospholipid syndrome patients treated with direct oral anticoagulants. Results from an international patient-level data Meta-analysis. Research and Practice in Thrombosis and Haemostasis 2019;3(Supplement 1):183-84. doi: http://dx.doi.org/10.1002/rth2.12227

121. Dufrost V, Risse J, Reshetnyak T, et al. Increased risk of thrombosis in antiphospholipid syndrome patients treated with direct oral anticoagulants. Results from an international patient-level data meta-analysis. Autoimmunity Reviews 2018;17:1011-21. doi: https://dx.doi.org/10.1016/j.autrev.2018.04.009

122. Dufrost V, Wahl D, Zuily S. Direct oral anticoagulants in antiphospholipid syndrome: Meta-analysis of randomized controlled trials. Autoimmunity Reviews 2021;20:102711. doi: https://dx.doi.org/10.1016/j.autrev.2020.102711

123. Elsebaie M, Langston A, Gaddh M. Direct oral anti-coagulants in patients with venous thromboembolism and thrombophilia: A systematic review and meta-analysis. Am J Hematol 2018;93(9):E17. doi: http://dx.doi.org/10.1002/ajh.25268

124. Elsebaie MAT, van Es N, Langston A, et al. Direct oral anticoagulants in patients with venous thromboembolism and thrombophilia: a systematic review and meta-analysis. Journal of Thrombosis & Haemostasis 2019;17:645-56. doi: https://dx.doi.org/10.1111/jth.14398

125. Elshafei MN, Mohamed MFH, El-Bardissy A, et al. Comparative effectiveness and safety of direct oral anticoagulants compared to warfarin in morbidly obese patients with acute venous thromboembolism: systematic review and a meta-analysis. Journal of Thrombosis & Thrombolysis 2021;51:388-96. doi: https://dx.doi.org/10.1007/s11239-020-02179-4

126. Enache B, Del Castillo-Carnevali H, Lairez O, et al. Minimally interrupted versus uninterrupted non-vitamin K anticoagulants for atrial fibrillation ablation. a meta-analysis of randomized controlled trials. Europace 2020;22(SUPPL 1):i201.

127. Escobar C, Barrios V, Lip GYH, et al. Effectiveness and Safety of Dabigatran Compared to Vitamin K Antagonists in Non-Asian Patients with Atrial Fibrillation: A Systematic Review and Meta-Analysis. Clin Drug Investig 2021;41:941-53. doi: https://dx.doi.org/10.1007/s40261-021-01091-w

128. Escobar C, Marti-Almor J, Perez C, et al. Direct Oral Anticoagulants Versus Vitamin K Antagonists in Real-life Patients With Atrial Fibrillation. A Systematic Review and Meta-analysis. Rev Esp Cardiol 2019;72:305-16. doi: https://dx.doi.org/10.1016/j.rec.2018.03.009

129. Escobar C, Marti-Almor J, Perez C, et al. Direct Oral Anticoagulants Versus Vitamin K Antagonists in Real-life Patients With Atrial Fibrillation. A Systematic Review and Meta-analysis Anticoagulantes orales directos frente a antagonistas de la vitamina K en pacientes con fibrilacion auricular de la practica clinica: revision sistematica y metanalisis. Rev Esp Cardiol 2019;72(4):305-16. doi: http://dx.doi.org/10.1016/j.recesp.2018.02.023

130. Eyileten C, Postula M, Jakubik D, et al. Non-Vitamin K Oral Anticoagulants (NOAC) Versus Vitamin K Antagonists (VKA) for Atrial Fibrillation with Elective or Urgent Percutaneous Coronary Intervention: A Meta-Analysis with a Particular Focus on Combination Type. Journal of Clinical Medicine 2020;9:14. doi: https://dx.doi.org/10.3390/jcm9041120

131. Feldberg J, Patel P, Farrell A, et al. A systematic review of direct oral anticoagulant use in chronic kidney disease and dialysis patients with atrial fibrillation. Nephrol Dial Transplant 2019;34:265-77. doi: https://dx.doi.org/10.1093/ndt/gfy031

132. Feng W, Wang X, Huang D, et al. Ranking the efficacy of anticoagulants for the prevention of venous thromboembolism after total hip or knee arthroplasty: A systematic review and a network meta-analysis. Pharmacol Res 2021;166:105438. doi: https://dx.doi.org/10.1016/j.phrs.2021.105438

133. Fiordellisi W, White K, Schweizer M. A Systematic Review and Meta-analysis of the Association Between Vitamin K Antagonist Use and Fracture. J Gen Intern Med 2019;34:304-11. doi: https://dx.doi.org/10.1007/s11606-018-4758-2

134. Flumignan RL, Tinoco JDS, Pascoal PI, et al. Prophylactic anticoagulants for people hospitalised with COVID-19. Cochrane Database of Systematic Reviews 2020;10:CD013739. doi: https://dx.doi.org/10.1002/14651858.CD013739

135. Fu Y, Zhu W, Zhou Y, et al. Non-vitamin K Antagonist Oral Anticoagulants Versus Warfarin in Patients with Atrial Fibrillation and Liver Disease: A Meta-Analysis and Systematic Review. Am J Cardiol 2020;20:139-47. doi: https://dx.doi.org/10.1007/s40256-019-00369-x

136. Fuentes HE, McBane R, Wysokinski W, et al. Direct oral factor Xa inhibitors for the treatment of acute cancer-associated venous thromboembolism: A systematic review and network meta-analysis. Journal of Clinical Oncology Conference 2019;37 doi: http://dx.doi.org/10.1200/JCO.2019.37.15_suppl.e23156

137. Fuentes HE, McBane RD, nd, et al. Direct Oral Factor Xa Inhibitors for the Treatment of Acute Cancer-Associated Venous Thromboembolism: A Systematic Review and Network Meta-analysis. Mayo Clin Proc 2019;94:2444-54. doi: https://dx.doi.org/10.1016/j.mayocp.2019.05.035

138. Galli M, Andreotti F, D'Amario D, et al. Dual therapy with direct oral anticoagulants significantly increases the risk of stent thrombosis compared to triple therapy. European Heart Journal - Cardiovascular Pharmacotherapy 2020;6(2):128-29. doi: http://dx.doi.org/10.1093/ehjcvp/pvz030

139. Garcia NT, Dahal K, Apte N, et al. Safety and efficacy of non-vitamin k antagonist oral anticoagulants versus warfarin in patients undergoing atrial fibrillation ablation: A meta-analysis of randomized controlled trials. Journal of the American College of Cardiology Conference: 67th Annual Scientific Session of the American College of Cardiology and i2 Summit: Innovation in Intervention, ACC 2018;71 doi: http://dx.doi.org/10.1016/S0735-1097%2818%2931020-9

140. Garg J, Chaudhary R, Krishnamoorthy P, et al. Safety of uninterrupted periprocedural apixaban in patients undergoing atrial fibrillation catheter ablation: A meta-analysis of 1,057 patients. J Am Coll Cardiol 2016;1):732.

141. Ge Z, Faggioni M, Baber U, et al. Safety and efficacy of nonvitamin K antagonist oral anticoagulants during catheter ablation of atrial fibrillation: A systematic review and meta-analysis. Cardiovascular therapeutics 2018;36:e12457. doi: https://dx.doi.org/10.1111/1755-5922.12457

142. Ge Z, Faggioni M, Usman B, et al. Safety and efficacy of non-vitamin K antagonist oral anticoagulants during transcatheter ablation of atrial fibrillation. Circulation Conference: Resuscitation Science Symposium, ReSS 2017;136

143. Ghaffarpasand E, Tehrani MD, Marszalek J, et al. Non-vitamin K antagonist oral anticoagulants for the treatment of intracardiac thrombosis. Journal of Thrombosis & Thrombolysis 2018;46:332-38. doi: https://dx.doi.org/10.1007/s11239-018-1693-3

144. Gibson CM, Basto AN, Howard ML. Direct Oral Anticoagulants in Cardioversion: A Review of Current Evidence. Ann Pharmacother 2018;52:277-84. doi: https://dx.doi.org/10.1177/1060028017737095

145. Goeldlin M, Seiffge DJ, Tatlisumak T, et al. Meta-analysis of haematoma volume, haematoma expansion and mortality in intracerebral haemorrhage associated with oral anticoagulant use. Eur J Neurol 2019;26(Supplement 1):65. doi: http://dx.doi.org/10.1111/ene.14017

146. Gomez-Outes A, Terleira-Fernandez AI, Lecumberri R, et al. Causes of Death in Patients with Venous Thromboembolism Anticoagulated with Direct Oral Anticoagulants: A Systematic Review and Meta-Analysis. Semin Thromb Hemost 2018;44:377-87. doi: https://dx.doi.org/10.1055/s-0038-1642644

147. Gorla R, Dentali F, Crippa M, et al. Perioperative Safety and Efficacy of Different Anticoagulation Strategies With Direct Oral Anticoagulants in Pulmonary Vein Isolation: A Meta-Analysis. JACC Clinical Electrophysiology 2018;4:794-806. doi: https://dx.doi.org/10.1016/j.jacep.2018.04.006

148. Grajek S, Kaluzna-Oleksy M, Siller-Matula JM, et al. Non-Vitamin K Antagonist Oral Anticoagulants and Risk of Myocardial Infarction in Patients with Atrial Fibrillation with or without Percutaneous Coronary Interventions: A Meta-Analysis. Journal of Personalized Medicine 2021;11:09. doi: https://dx.doi.org/10.3390/jpm11101013

149. Grymonprez M, Steurbaut S, De Sutter A, et al. Impact of a single non-sex-related stroke risk factor on atrial fibrillation and oral anticoagulant outcomes: a systematic review and meta-analysis. Open Heart 2020;7:12. doi: https://dx.doi.org/10.1136/openhrt-2020-001465

150. Grymonprez M, Vanspranghe K, Steurbaut S, et al. Non-vitamin K Antagonist Oral Anticoagulants (NOACs) Versus Warfarin in Patients with Atrial Fibrillation Using P-gp and/or CYP450-Interacting Drugs: a Systematic Review and Meta-analysis. Cardiovascular Drugs & Therapy 2021;12:12. doi: https://dx.doi.org/10.1007/s10557-021-07279-8

151. Gu ZC, Kong LC, Yang SF, et al. Net clinical benefit of non-vitamin K antagonist oral anticoagulants in atrial fibrillation and chronic kidney disease: a trade-off analysis from four phase III clinical trials. Cardiovascular Diagnosis & Therapy 2019;9:410-19. doi: https://dx.doi.org/10.21037/cdt.2019.07.09

152. Gu ZC, Wei AH, Zhang C, et al. Risk of Major Gastrointestinal Bleeding With New vs Conventional Oral Anticoagulants: A Systematic Review and Meta-analysis. Clinical Gastroenterology & Hepatology 2020;18:792-99.e61. doi: https://dx.doi.org/10.1016/j.cgh.2019.05.056

153. Gu ZC, Yan YD, Yang SY, et al. Direct versus conventional anticoagulants for treatment of cancer associated thrombosis: a pooled and interaction analysis between observational studies and randomized clinical trials. Annals of Translational Medicine 2020;8:95. doi: https://dx.doi.org/10.21037/atm.2019.12.152

154. Gu ZC, Zhou LY, Shen L, et al. Non-vitamin K Antagonist Oral Anticoagulants vs. Warfarin at Risk of Fractures: A Systematic Review and Meta-Analysis of Randomized Controlled Trials. Frontiers in Pharmacology 2018;9:348. doi: https://dx.doi.org/10.3389/fphar.2018.00348

155. Gui YY, Zou S, Yang WL, et al. The impact of renal function on efficacy and safety of new oral anticoagulant in atrial fibrillation patients: A systemic review and meta-analysis. Medicine 2019;98:e18205. doi: https://dx.doi.org/10.1097/MD.0000000000018205

156. Guo WQ, Chen XH, Tian XY, et al. Differences In Gastrointestinal Safety Profiles Among Novel Oral Anticoagulants: Evidence From A Network Meta-Analysis. Clinical Epidemiology 2019;11:911-21. doi: https://dx.doi.org/10.2147/CLEP.S219335

157. Guo Z, Ding X, Ye Z, et al. Non-vitamin K antagonist oral anticoagulants versus vitamin K antagonists in atrial fibrillation patients with previous stroke or intracranial hemorrhage: A systematic review and meta-analysis of observational studies. Clin Cardiol 2021;44:917-24. doi: https://dx.doi.org/10.1002/clc.23647

158. Gupta S, Um KJ, Pandey A, et al. Direct Oral Anticoagulants Versus Vitamin K Antagonists in Patients Undergoing Cardioversion for Atrial Fibrillation: a Systematic Review and Meta-analysis. Cardiovascular Drugs & Therapy 2019;33:339-52. doi: https://dx.doi.org/10.1007/s10557-019-06869-x

159. Ha FJ, Barra S, Brown AJ, et al. Continuous and minimally-interrupted direct oral anticoagulant are both safe compared with vitamin K antagonist for atrial fibrillation ablation: An updated meta-analysis. Int J Cardiol 2018;262:51-56. doi: https://dx.doi.org/10.1016/j.ijcard.2018.03.095

160. Ha J, Neuen BL, Cheng LP, et al. Benefits and risks of oral anticoagulant therapy in CKD: A systematic review and meta-analysis of randomized controlled trials. J Am Soc Nephrol 2018;29:70.

161. Ha JT, Neuen BL, Cheng LP, et al. Benefits and Harms of Oral Anticoagulant Therapy in Chronic Kidney Disease: A Systematic Review and Meta-analysis. Annals of internal medicine 2019;171:181-89. doi: https://dx.doi.org/10.7326/M19-0087

162. Hage A, Dolan DP, Nasr VG, et al. Safety of Direct Oral Anticoagulants Compared to Warfarin for Atrial Fibrillation after Cardiac Surgery: A Systematic Review and Meta-Analysis. Semin Thorac Cardiovasc Surg 2021;08:08. doi: https://dx.doi.org/10.1053/j.semtcvs.2021.05.011

163. Hamulyak EN, Daams JG, Leebeek FWG, et al. A systematic review of antithrombotic treatment of venous thromboembolism in patients with myeloproliferative neoplasms. Blood Advances 2021;5:113-21. doi: https://dx.doi.org/10.1182/bloodadvances.2020003628

164. Harskamp RE, Teichert M, Lucassen WAM, et al. Impact of Polypharmacy and P-Glycoprotein- and CYP3A4-Modulating Drugs on Safety and Efficacy of Oral Anticoagulation Therapy in Patients with Atrial Fibrillation. Cardiovascular Drugs & Therapy 2019;33:615-23. doi: https://dx.doi.org/10.1007/s10557-019-06907-8

165. He H, Ke B, Li Y, et al. Novel oral anticoagulants in the preoperative period: a meta-analysis. Journal of Thrombosis & Thrombolysis 2018;45:386-96. doi: https://dx.doi.org/10.1007/s11239-018-1612-7

166. He T, Han F, Wang J, et al. Efficacy and safety of anticoagulants for postoperative thrombophylaxis in total hip and knee arthroplasty: A PRISMA-compliant Bayesian network meta-analysis. PLoS One 2021;16:e0250096. doi: https://dx.doi.org/10.1371/journal.pone.0250096

167. Hellfritzsch M, Adelborg K, Damkier P, et al. Effectiveness and safety of direct oral anticoagulants in atrial fibrillation patients switched from vitamin K antagonists: A systematic review and meta-analysis. Basic & Clinical Pharmacology & Toxicology 2019;25:25. doi: https://dx.doi.org/10.1111/bcpt.13283

168. Hill NR, Sandler B, Bergrath E, et al. A Systematic Review of Network Meta-Analyses and Real-World Evidence Comparing Apixaban and Rivaroxaban in Nonvalvular Atrial Fibrillation. Clinical & Applied Thrombosis/Hemostasis 2020;26:1076029619898764. doi: https://dx.doi.org/10.1177/1076029619898764

169. Hirschl M, Kundi M. New oral anticoagulants in the treatment of acute venous thromboembolism - A systematic review with indirect comparisons Neue orale antikoagulantien fur die behandlung der akuten venosen th rombo embolie - Eine systematische ubersichtsarbeit mit indirekten vergleichen. Vasa - European Journal of Vascular Medicine 2014;43(5):353-64. doi: http://dx.doi.org/10.1024/0301-1526/a000373

170. Hirschl M, Kundi M. Safety and efficacy of direct acting oral anticoagulants and vitamin K antagonists in nonvalvular atrial fibrillation - a network meta-analysis of real-world data. Vasa 2019;48:134-47. doi: https://dx.doi.org/10.1024/0301-1526/a000746

171. Hua W, Huang Z, Huang Z. Bleeding Outcomes After Dental Extraction in Patients Under Direct-Acting Oral Anticoagulants vs. Vitamin K Antagonists: A Systematic Review and Meta-Analysis. Frontiers in Pharmacology 2021;12:702057. doi: https://dx.doi.org/10.3389/fphar.2021.702057

172. Hua Y, Sun JY, Su Y, et al. The Safety and Efficacy of Rivaroxaban Compared with Warfarin in Patients with Atrial Fibrillation and Diabetes: A Systematic Review and Meta-analysis. Am J Cardiol 2021;21:51-61. doi: https://dx.doi.org/10.1007/s40256-020-00407-z

173. Huang HK, Peng CC, Lin SM, et al. Fracture Risks in Patients Treated With Different Oral Anticoagulants: A Systematic Review and Meta-Analysis. Journal of the American Heart Association 2021;10:e019618. doi: https://dx.doi.org/10.1161/JAHA.120.019618

174. Huang WY, Singer DE, Wu YL, et al. Comparison of major bleeding endpoints between non-vitamin k antagonist oral anticoagulants and aspirin: a network meta-analysis. Stroke Conference: American Heart Association/American Stroke Association 2018;49

175. Huang ZC, Li CQ, Liu XY, et al. Efficacy and Safety of Direct Oral Anticoagulants in Patients with Atrial Fibrillation and Liver Disease: a Meta-Analysis and Systematic Review. Cardiovascular Drugs & Therapy 2021;35:1205-15. doi: https://dx.doi.org/10.1007/s10557-020-07065-y

176. Iriarte F, Cerda Serra P, Mora L, et al. Direct oral anticoagulants versus vitamin k antagonists in antiphospholipid syndrome: A meta-analysis. Research and Practice in Thrombosis and Haemostasis 2020;4(SUPPL 1):958-59. doi: http://dx.doi.org/10.1002/rth2.12393

177. Jackson C, Cristancho C, Seth A. Effect of aspirin use on the efficacy and safety of the direct oral anticoagulants in patients with nonvalvular atrial fibrillation: A systematic review and meta-analysis. Journal of Hospital Medicine Conference: Hospital Medicine, HM 2018;13

178. Jiang H, Jiang Y, Ma H, et al. Effects of rivaroxaban and warfarin on the risk of gastrointestinal bleeding and intracranial hemorrhage in patients with atrial fibrillation: Systematic review and meta-analysis. Clin Cardiol 2021;44:1208-15. doi: https://dx.doi.org/10.1002/clc.23690

179. Jiang R, Shi Y, Zhang R, et al. Comparative efficacy and safety of low-intensity warfarin therapy in preventing unprovoked recurrent venous thromboembolism: A systematic review and meta-analysis. The clinical respiratory journal 2018;12:2170-77. doi: https://dx.doi.org/10.1111/crj.12795

180. Jin H, Zhu K, Wang L, et al. Efficacy and Safety of Non-Vitamin K Anticoagulants for Atrial Fibrillation in Relation to Different Renal Function Levels: A Network Meta-Analysis. Cardiovascular therapeutics 2020;2020:2683740. doi: https://dx.doi.org/10.1155/2020/2683740

181. Jin H, Zhu K, Wang L, et al. A network meta-analysis of non-vitamin K antagonist oral anticoagulants versus warfarin in patients with atrial fibrillation and diabetes mellitus. Acta Cardiol 2021:1-10. doi: https://dx.doi.org/10.1080/00015385.2020.1869671

182. Jin H, Zhu K, Wang L, et al. Efficacy and safety of non-vitamin K anticoagulants and warfarin in patients with atrial fibrillation and heart failure: A network meta-analysis. Thromb Res 2020;196:109-19. doi: https://dx.doi.org/10.1016/j.thromres.2020.08.021

183. Kahale LA, Hakoum MB, Tsolakian IG, et al. Anticoagulation for the long-term treatment of venous thromboembolism in people with cancer. Cochrane Database of Systematic Reviews 2018;6:CD006650. doi: https://dx.doi.org/10.1002/14651858.CD006650.pub5

184. Kahale LA, Matar CF, Tsolakian I, et al. Oral anticoagulation in people with cancer who have no therapeutic or prophylactic indication for anticoagulation. Cochrane Database of Systematic Reviews 2021;10:CD006466. doi: https://dx.doi.org/10.1002/14651858.CD006466.pub7

185. Kajy M, Mathew A, Ramappa P. Treatment Failures of Direct Oral Anticoagulants. Am J Ther 2021;28:e87-e95. doi: https://dx.doi.org/10.1097/MJT.0000000000001083

186. Kajy M, Shokr M, Ramappa P. Use of Direct Oral Anticoagulants in the Treatment of Left Ventricular Thrombus: Systematic Review of Current Literature. Am J Ther 2020;27:e584-e90. doi: https://dx.doi.org/10.1097/MJT.0000000000000937

187. Kang F, Ma Y, Cai A, et al. Meta-Analysis Evaluating the Efficacy and Safety of Low-Intensity Warfarin for Patients >65 Years of Age With Non-Valvular Atrial Fibrillation. Am J Cardiol 2021;142:74-82. doi: https://dx.doi.org/10.1016/j.amjcard.2020.12.001

188. Karathanos C, Nana P, Spanos K, et al. Efficacy of rivaroxaban in prevention of post-thrombotic syndrome: A systematic review and meta-analysis. J Vasc Surg 2021;9:1568-76.e1. doi: https://dx.doi.org/10.1016/j.jvsv.2021.04.016

189. Katsanos AH, Schellinger PD, Kohrmann M, et al. Fatal oral anticoagulant-related intracranial hemorrhage: a systematic review and meta-analysis. Eur J Neurol 2018;25:1299-302. doi: https://dx.doi.org/10.1111/ene.13742

190. Khan F, Tritschler T, Kimpton M, et al. Long-Term Risk for Major Bleeding During Extended Oral Anticoagulant Therapy for First Unprovoked Venous Thromboembolism : A Systematic Review and Meta-analysis. Annals of internal medicine 2021;174:1420-29. doi: https://dx.doi.org/10.7326/M21-1094

191. Khan F, Tritschler T, Kimpton M, et al. Long-term risk of recurrent venous thromboembolism among patients receiving extended oral anticoagulant therapy for first unprovoked venous thromboembolism: A systematic review and meta-analysis. Journal of Thrombosis & Haemostasis 2021;19:2801-13. doi: https://dx.doi.org/10.1111/jth.15491

192. Khanra D, Mukherjee A, Deshpande S, et al. A Network Meta-Analysis Comparing Osteoporotic Fracture among Different Direct Oral Anticoagulants and Vitamin K Antagonists in Patients with Atrial Fibrillation. Journal of Bone Metabolism 2021;28:139-50. doi: https://dx.doi.org/10.11005/jbm.2021.28.2.139

193. Khanra D, Ray IB, Duggal B. Bleeding outcome of peri-procedural anticoagulation in patients of atrial fibrillation undergoing catheter ablation: A network meta-analysis. Journal of Arrhythmia 2019;35(Supplement 1):76-77. doi: http://dx.doi.org/10.1002/joa3.12267

194. Khodashahi M, Rezaieyazdi Z, Sahebari M. Comparison of the Therapeutic Effects of Rivaroxaban Versus Warfarin in Antiphospholipid Syndrome: A Systematic Review. Archives of Rheumatology 2020;35:107-16. doi: https://dx.doi.org/10.5606/ArchRheumatol.2020.7375

195. Kido K, Ghaffar YA, Lee JC, et al. Meta-analysis comparing direct oral anticoagulants versus vitamin K antagonists in patients with left ventricular thrombus. PLoS One 2021;16:e0252549. doi: https://dx.doi.org/10.1371/journal.pone.0252549

196. Kido K, Lee JC, Hellwig T, et al. Use of Direct Oral Anticoagulants in Morbidly Obese Patients. Pharmacotherapy:The Journal of Human Pharmacology & Drug Therapy 2020;40:72-83. doi: https://dx.doi.org/10.1002/phar.2353

197. Kido K, Shimizu M, Shiga T, et al. Meta-Analysis Comparing Direct Oral Anticoagulants Versus Warfarin in Morbidly Obese Patients With Atrial Fibrillation. Am J Cardiol 2020;126:23-28. doi: https://dx.doi.org/10.1016/j.amjcard.2020.03.048

198. Kido K, Shimizu M, Shiga T, et al. Network Meta-Analysis Comparing Apixaban Versus Rivaroxaban in Morbidly Obese Patients With Atrial Fibrillation. Am J Cardiol 2020;134:160-61. doi: http://dx.doi.org/10.1016/j.amjcard.2020.09.003

199. Kido K, Shimizu M, Shiga T, et al. Meta-analysis comparing inappropriately low dose versus standard dose of direct oral anticoagulants in patients with atrial fibrillation. J Am Pharm Assoc 2021;27:27. doi: https://dx.doi.org/10.1016/j.japh.2021.10.027

200. Kim IS, Kim HJ, Kim TH, et al. Appropriate doses of non-vitamin K antagonist oral anticoagulants in high-risk subgroups with atrial fibrillation: Systematic review and meta-analysis. J Cardiol 2018;72:284-91. doi: https://dx.doi.org/10.1016/j.jjcc.2018.03.009

201. Kim IS, Kim HJ, Kim TH, et al. Non-vitamin K antagonist oral anticoagulants have better efficacy and equivalent safety compared to warfarin in elderly patients with atrial fibrillation: A systematic review and meta-analysis. J Cardiol 2018;72:105-12. doi: https://dx.doi.org/10.1016/j.jjcc.2018.01.015

202. Kim IS, Kim HJ, Kim TH, et al. Non-vitamin k antagonist oral anticoagulants in high risk-subgroups with atrial fibrillation: Systematic review and meta-analysis. Europace 2018;20(Supplement 1):i69. doi: http://dx.doi.org/10.1093/europace/euy015

203. Kim IS, Kim HJ, Yu HT, et al. Non-vitamin K antagonist oral anticoagulants with amiodarone, P-glycoprotein inhibitors, or polypharmacy in patients with atrial fibrillation: Systematic review and meta-analysis. J Cardiol 2019;73:515-21. doi: https://dx.doi.org/10.1016/j.jjcc.2018.12.018

204. Kim SM, Jeon ET, Jung JM, et al. Real-world oral anticoagulants for Asian patients with non-valvular atrial fibrillation: A PRISMA-compliant article. Medicine 2021;100:e26883. doi: https://dx.doi.org/10.1097/MD.0000000000026883

205. Kirkilesis GI, Kakkos SK, Tsolakis IA. Editor's Choice - A Systematic Review and Meta-Analysis of the Efficacy and Safety of Anticoagulation in the Treatment of Venous Thromboembolism in Patients with Cancer. European Journal of Vascular & Endovascular Surgery 2019;57:685-701. doi: https://dx.doi.org/10.1016/j.ejvs.2018.11.004

206. Kirkilesis GI, Kakkos SK, Tsolakis IA. A Systematic Review and Meta-Analysis of the Efficacy and Safety of Anticoagulation in the Treatment of Venous Thromboembolism in Patients with Cancer. J Vasc Surg 2019;69(6):2007. doi: http://dx.doi.org/10.1016/j.jvs.2019.04.474

207. Kitano T, Nabeshima Y, Kataoka M, et al. Therapeutic efficacy of direct oral anticoagulants and vitamin K antagonists for left ventricular thrombus: Systematic review and meta-analysis. PLoS One 2021;16:e0255280. doi: https://dx.doi.org/10.1371/journal.pone.0255280

208. Klarenbach S, Lee K, Boucher M, et al. Direct Oral Anticoagulants for the Treatment of Venous Thromboembolic Events: Economic Evaluation. Canadian Agency for Drugs and Technologies in Health CADTH Health Technology Assessments 2016;03:03.

209. Knijnik L, Rivera M, Blumer V, et al. Prevention of Stroke in Atrial Fibrillation After Coronary Stenting. Stroke 2019;50:2125-32. doi: https://dx.doi.org/10.1161/STROKEAHA.119.026078

210. Koh JH, Liew ZH, Ng GK, et al. Efficacy and safety of direct oral anticoagulants versus vitamin K antagonist for portal vein thrombosis in cirrhosis: A systematic review and meta-analysis. Dig Liver Dis 2021;12:12. doi: https://dx.doi.org/10.1016/j.dld.2021.07.039

211. Komocsi A, Sharif S, Kehl D, et al. Mortality and stroke prevention in atrial fibrillation; network meta-analysis of real world data. Eur Heart J 2018;39(Supplement 1):695. doi: http://dx.doi.org/10.1093/eurheartj/ehy563.P3447

212. Kotecha D, Pollack CV, De Caterina R, et al. Direct Oral Anticoagulants Halve Thromboembolic Events After Cardioversion of AF Compared With Warfarin. J Am Coll Cardiol 2018;72(16):1984-86. doi: http://dx.doi.org/10.1016/j.jacc.2018.07.083

213. Koval N, Alves M, Placido R, et al. Direct oral anticoagulants versus vitamin K antagonists in patients with antiphospholipid syndrome: systematic review and meta-analysis. RMD Open 2021;7:07. doi: https://dx.doi.org/10.1136/rmdopen-2021-001678

214. Kumar D, Warsha FNU, Helmstetter N, et al. Efficacy and safety of direct oral anticoagulants for treatment of left ventricular thrombus; a systematic review. Acta Cardiol 2021;76:825-29. doi: https://dx.doi.org/10.1080/00015385.2021.1901024

215. Kuno T, Takagi H, Ando T, et al. Oral Anticoagulation for Patients With Atrial Fibrillation on Long-Term Hemodialysis. J Am Coll Cardiol 2020;75:273-85. doi: https://dx.doi.org/10.1016/j.jacc.2019.10.059

216. Kuno T, Ueyama H, Takagi H, et al. Meta-analysis of Antithrombotic Therapy in Patients With Atrial Fibrillation Undergoing Percutaneous Coronary Intervention. Am J Cardiol 2020;125:521-27. doi: https://dx.doi.org/10.1016/j.amjcard.2019.11.022

217. Kupo P, Szakacs Z, Solymar M, et al. Direct Anticoagulants and Risk of Myocardial Infarction, a Multiple Treatment Network Meta-Analysis. Angiology 2020;71:27-37. doi: https://dx.doi.org/10.1177/0003319719874255

218. Lapumnuaypol K, Chiasakul T. Safety of direct oral anticoagulants in patients with cirrhosis: A systematic review and meta-analysis. Blood Conference: 60th Annual Meeting of the American Society of Hematology, ASH 2018;132 doi: http://dx.doi.org/10.1182/blood-2018-99-117255

219. Lapumnuaypol K, DiMaria C, Chiasakul T. Safety of direct oral anticoagulants in patients with cirrhosis: a systematic review and meta-analysis. QJM 2019;112:605-10. doi: https://dx.doi.org/10.1093/qjmed/hcz127

220. Lee GKH, Chen VH, Tan CH, et al. Comparing the efficacy and safety of direct oral anticoagulants with vitamin K antagonist in cerebral venous thrombosis. Journal of Thrombosis & Thrombolysis 2020;50:724-31. doi: https://dx.doi.org/10.1007/s11239-020-02106-7

221. Lee JJ, Ha ACT, Dorian P, et al. Meta-Analysis of Safety and Efficacy of Direct Oral Anticoagulants Versus Warfarin According to Time in Therapeutic Range in Atrial Fibrillation. Am J Cardiol 2021;140:62-68. doi: https://dx.doi.org/10.1016/j.amjcard.2020.10.064

222. Lee KG, Chen VHE, Tan CH, et al. Comparison of the efficacyand safety of doac with vka in patients with cerebral venous thrombosis-a systematic review and meta-analysis. International Journal of Stroke 2020;15(1 SUPPL):653-54. doi: http://dx.doi.org/10.1177/1747493020963387

223. Lee M, Wang M, Liu J, et al. Do telehealth interventions improve oral anticoagulation management? A systematic review and meta-analysis. Journal of Thrombosis & Thrombolysis 2018;45:325-36. doi: https://dx.doi.org/10.1007/s11239-018-1609-2

224. Lee MC, Liao CT, Toh HS. Systematic review and network meta-analysis: The efficacy and safety of oral anticoagulants in patients with atrial fibrillation in asian. Eur Heart J 2018;39(Supplement 1):1408-09. doi: http://dx.doi.org/10.1093/eurheartj/ehy566.P6582

225. Lee ZX, Ang E, Lim XT, et al. Association of Risk of Dementia With Direct Oral Anticoagulants Versus Warfarin Use in Patients With Non-valvular Atrial Fibrillation: A Systematic Review and Meta-analysis. J Cardiovasc Pharmacol 2021;77:22-31. doi: https://dx.doi.org/10.1097/FJC.0000000000000925

226. Lee ZY, Suah BH, Teo YH, et al. Comparison of the Efficacy and Safety of Direct Oral Anticoagulants and Vitamin K Antagonists in Patients with Atrial Fibrillation and Concomitant Liver Cirrhosis: A Systematic Review and Meta-Analysis. Am J Cardiol 2021;19:19. doi: https://dx.doi.org/10.1007/s40256-021-00482-w

227. Leow AS, Sia CH, Tan BY, et al. A meta-summary of case reports of non-vitamin K antagonist oral anticoagulant use in patients with left ventricular thrombus. Journal of Thrombosis & Thrombolysis 2018;46:68-73. doi: https://dx.doi.org/10.1007/s11239-018-1656-8

228. Lewis S, Glen J, Dawoud D, et al. Venous Thromboembolism Prophylaxis Strategies for People Undergoing Elective Total Hip Replacement: A Systematic Review and Network Meta-Analysis. Value Health 2019;22:953-69. doi: https://dx.doi.org/10.1016/j.jval.2019.02.013

229. Lewis S, Glen J, Dawoud D, et al. Venous thromboembolism prophylaxis strategies for people undergoing elective total knee replacement: a systematic review and network meta-analysis. The Lancet Haematology 2019;6:e530-e39. doi: https://dx.doi.org/10.1016/S2352-3026(19)30155-3

230. Li G, Lip GYH, Holbrook A, et al. Direct comparative effectiveness and safety between non-vitamin K antagonist oral anticoagulants for stroke prevention in nonvalvular atrial fibrillation: a systematic review and meta-analysis of observational studies. Eur J Epidemiol 2019;34:173-90. doi: https://dx.doi.org/10.1007/s10654-018-0415-7

231. Li G, Zeng J, Zhang J, et al. Comparative Effects Between Direct Oral Anticoagulants for Acute Venous Thromboembolism: Indirect Comparison From Randomized Controlled Trials. Frontiers in Medicine 2020;7:280. doi: https://dx.doi.org/10.3389/fmed.2020.00280

232. Li H, Yao M, Liao S, et al. Comparison of Novel Oral Anticoagulants and Vitamin K Antagonists in Patients With Cerebral Venous Sinus Thrombosis on Efficacy and Safety: A Systematic Review. Frontiers in neurology [electronic resource] 2020;11:597623. doi: https://dx.doi.org/10.3389/fneur.2020.597623

233. Li R, Yuan M, Cheng J, et al. Risk of post-thrombotic syndrome after deep vein thrombosis treated with rivaroxaban versus vitamin-K antagonists: A systematic review and meta-analysis. Thromb Res 2020;196:340-48. doi: https://dx.doi.org/10.1016/j.thromres.2020.09.014

234. Li S, Deng Y, Tong Y, et al. Assessment of non-vitamin K antagonist oral anticoagulants for the management of left ventricular thrombus. Clin Cardiol 2021;44:754-60. doi: https://dx.doi.org/10.1002/clc.23553

235. Li S, Liu B, Xu D, et al. Bleeding risk and mortality of edoxaban: A pooled meta- analysis of randomized controlled trials. PLoS ONE 2014;9(4) (no pagination) doi: http://dx.doi.org/10.1371/journal.pone.0095354.g003

236. Li W, Kokkinidis D, Wang YC. Efficacy and Safety of Dabigatran, Rivaroxaban, and Apixaban Compared to Warfarin in Asian Patients with Non-valvular Atrial Fibrillation: A Systemic Review and Meta-analysis. Circulation Conference: American Heart Association Scientific Sessions, AHA 2020;142 doi: http://dx.doi.org/10.1161/circ.142.suppl_3.14825

237. Li WJ, Archontakis-Barakakis P, Palaiodimos L, et al. Dabigatran, rivaroxaban, and apixaban are superior to warfarin in Asian patients with non-valvular atrial fibrillation: An updated meta-analysis. World Journal of Cardiology 2021;13:82-94. doi: https://dx.doi.org/10.4330/wjc.v13.i4.82

238. Liang H, He Q, Zhang Q, et al. Efficacy and safety outcomes in novel oral anticoagulants versus vitamin-K antagonist on post-TAVI patients: a meta-analysis. BMC Cardiovasc Disord 2020;20:307. doi: https://dx.doi.org/10.1186/s12872-020-01582-2

239. Liang X, Xie W, Lin Z, et al. The efficacy and safety of edoxaban versus warfarin in preventing clinical events in atrial fibrillation: A systematic review and meta-analysis. Anatolian Journal of Cardiology 2021;25:77-88. doi: https://dx.doi.org/10.14744/AnatolJCardiol.2020.18049

240. Liao B, Wang TKM. Optimal antithrombotic strategy for patients with atrial fibrillation and acute coronary syndrome or percutaneous coronary intervention: updated meta-analysis of randomised controlled trials. Eur Heart J 2020;41(SUPPL 2):1725. doi: http://dx.doi.org/10.1093/ehjci/ehaa946.1725

241. Liao CT, Lee MC, Chen ZC, et al. Cost-Effectiveness Analysis of Oral Anticoagulants in Stroke Prevention among Patients with Atrial Fibrillation in Taiwan. Acta Cardiologica Sinica 2020;36:50-61. doi: https://dx.doi.org/10.6515/ACS.202001_36(1).20190511A

242. Liao XZ, Fu YH, Ma JY, et al. Non-Vitamin K Antagonist Oral Anticoagulants Versus Warfarin in Patients with Atrial Fibrillation and Peripheral Artery Disease: a Systematic Review and Meta-Analysis. Cardiovascular Drugs & Therapy 2020;34:391-99. doi: https://dx.doi.org/10.1007/s10557-020-06962-6

243. Liu F, Xu Z, Luo J, et al. Effectiveness and Safety of DOACs vs. VKAs in AF Patients With Cancer: Evidence From Randomized Clinical Trials and Observational Studies. Frontiers in Cardiovascular Medicine 2021;8:766377. doi: https://dx.doi.org/10.3389/fcvm.2021.766377

244. Liu F, Yang Y, Cheng W, et al. Reappraisal of Non-vitamin K Antagonist Oral Anticoagulants in Atrial Fibrillation Patients: A Systematic Review and Meta-Analysis. Frontiers in Cardiovascular Medicine 2021;8:757188. doi: https://dx.doi.org/10.3389/fcvm.2021.757188

245. Liu J, Wu Y, Li S, et al. The Efficacy of Non-Vitamin K Antagonist Oral Anticoagulants in the Prevention of Left Atrial Thrombus in Patients With Atrial Fibrillation Compared With Vitamin K Antagonists: A Meta-Analysis. Heart Surgery Forum 2020;23:E733-E39. doi: https://dx.doi.org/10.1532/hsf.3203

246. Liu M, Zheng Y, Li G. Safety of Recanalization Therapy in Patients with Acute Ischemic Stroke Under Anticoagulation: A Systematic Review and Meta-Analysis. Journal of Stroke & Cerebrovascular Diseases 2018;27:2296-305. doi: https://dx.doi.org/10.1016/j.jstrokecerebrovasdis.2018.04.012

247. Liu X, Huang M, Ye C, et al. Effect of non-recommended doses versus recommended doses of direct oral anticoagulants in atrial fibrillation patients: A meta-analysis. Clin Cardiol 2021;44:472-80. doi: https://dx.doi.org/10.1002/clc.23586

248. Liu X, Huang M, Ye C, et al. The role of non-vitamin K antagonist oral anticoagulants in Asian patients with atrial fibrillation: A PRISMA-compliant article. Medicine 2020;99:e21025. doi: https://dx.doi.org/10.1097/MD.0000000000021025

249. Liu X, Xu ZX, Yu P, et al. Non-Vitamin K Antagonist Oral Anticoagulants in Secondary Stroke Prevention in Atrial Fibrillation Patients: An Updated Analysis by Adding Observational Studies. Cardiovascular Drugs & Therapy 2020;34:569-78. doi: https://dx.doi.org/10.1007/s10557-020-06961-7

250. Liu XH, Gao XF, Chen B, et al. Uninterrupted peri-procedural anticoagulation with new oral anticoagulants in atrial fibrillation ablation: Insights from an updated meta-analysis. Journal of Arrhythmia 2019;35(Supplement 1):100. doi: http://dx.doi.org/10.1002/joa3.12267

251. Liu XH, Gao XF, Chen CF, et al. Thromboembolism and bleeding risk in atrial fibrillation ablation with uninterrupted anticoagulation between new oral anticoagulants and vitamin K antagonists: insights from an updated meta-analysis. Journal of Thrombosis & Thrombolysis 2020;50:201-10. doi: https://dx.doi.org/10.1007/s11239-019-01989-5

252. Liu Z, Ma L, Zhang H, et al. Comparison of non-vitamin K antagonist oral anticoagulants on bleeding and thrombosis. J Clin Pharm Ther 2021;46:1729-42. doi: https://dx.doi.org/10.1111/jcpt.13514

253. Lobraico-Fernandez J, Baksh S, Nemec E. Elderly Bleeding Risk of Direct Oral Anticoagulants in Nonvalvular Atrial Fibrillation: A Systematic Review and Meta-Analysis of Cohort Studies. Drugs R D 2019;19:235-45. doi: https://dx.doi.org/10.1007/s40268-019-0275-y

254. Lopes RD, Hong H, Harskamp RE, et al. Safety and Efficacy of Antithrombotic Strategies in Patients With Atrial Fibrillation Undergoing Percutaneous Coronary Intervention: A Network Meta-analysis of Randomized Controlled Trials. JAMA Cardiology 2019;4:747-55. doi: https://dx.doi.org/10.1001/jamacardio.2019.1880

255. Lorenzoni V, Pirri S, Turchetti G. Cost-Effectiveness of Direct Non-Vitamin K Oral Anticoagulants Versus Vitamin K Antagonists for the Management of Patients with Non-Valvular Atrial Fibrillation Based on Available "Real-World" Evidence: The Italian National Health System Perspective. Clin Drug Investig 2021;41:255-67. doi: https://dx.doi.org/10.1007/s40261-021-01002-z

256. Lowenstern A, Al-Khatib SM, Sharan L, et al. Interventions for Preventing Thromboembolic Events in Patients With Atrial Fibrillation: A Systematic Review. Annals of internal medicine 2018;169:774-87. doi: https://dx.doi.org/10.7326/M18-1523

257. Lozier MR, Sanchez A, Escolar E, et al. Comparison of Direct Oral Anticoagulants Versus Vitamin K Antagonists in Patients with Atrial Fibrillation in the Setting of Hypertrophic Cardiomyopathy: A Meta-Analysis. J Am Coll Cardiol 2020;75(11):480. doi: http://dx.doi.org/10.1016/S0735-1097%2820%2931107-4

258. Lozier MR, Sanchez AM, Lee JJ, et al. Thromboembolic Outcomes of Different Anticoagulation Strategies for Patients with Atrial Fibrillation in the Setting of Hypertrophic Cardiomyopathy: A Systematic Review. Journal of Atrial Fibrillation 2019;12:2207. doi: https://dx.doi.org/10.4022/jafib.2207

259. Lupercio F, Romero J, Peltzer B, et al. Efficacy and Safety Outcomes of Direct Oral Anticoagulants and Amiodarone in Patients with Atrial Fibrillation. Am J Med 2018;131:573.e1-73.e8. doi: https://dx.doi.org/10.1016/j.amjmed.2017.11.047

260. Lurie A, Wang J, Hinnegan KJ, et al. Prevalence of Left Atrial Thrombus in Anticoagulated Patients With Atrial Fibrillation. J Am Coll Cardiol 2021;77:2875-86. doi: https://dx.doi.org/10.1016/j.jacc.2021.04.036

261. Lv M, Wu T, Jiang S, et al. Risk of Intracranial Hemorrhage Caused by Direct Oral Anticoagulants for Stroke Prevention in Patients With Atrial Fibrillation (from a Network Meta-Analysis of Randomized Controlled Trials). Am J Cardiol 2021;28:28. doi: https://dx.doi.org/10.1016/j.amjcard.2021.09.011

262. Ma T, Liu C, Jiang T, et al. Comparative risk for intracranial hemorrhage related to new oral anticoagulants: A network meta-analysis. Medicine 2021;100:e24522. doi: https://dx.doi.org/10.1097/MD.0000000000024522

263. Mai V, Bertoletti L, Cucherat M, et al. Extended anticoagulation for the secondary prevention of venous thromboembolic events: An updated network meta-analysis. PLoS One 2019;14:e0214134. doi: https://dx.doi.org/10.1371/journal.pone.0214134

264. Mai V, Bertoletti L, Mainbourg S, et al. Extended anticoagulation for the secondary prevention of venous thromboembolic events: An updated network meta-analysis. Fundam Clin Pharmacol 2019;33(Supplement 1):13. doi: http://dx.doi.org/10.1111/fcp.12468

265. Mai V, Provencher S, Cucherat M, et al. Treatment effect of rivaroxaban in the extended treatment of venous thromboembolism: A comparative network meta-analysis. American Journal of Respiratory and Critical Care Medicine Conference: American Thoracic Society International Conference, ATS 2018;197

266. Mainbourg S, Cucherat M, Provencher S, et al. Twice- or Once-Daily Dosing of Direct Oral Anticoagulants, a systematic review and meta-analysis. Thromb Res 2021;197:24-32. doi: https://dx.doi.org/10.1016/j.thromres.2020.10.011

267. Makam RCP, Hoaglin DC, McManus DD, et al. Efficacy and safety of direct oral anticoagulants approved for cardiovascular indications: Systematic review and meta-analysis. PLoS One 2018;13:e0197583. doi: https://dx.doi.org/10.1371/journal.pone.0197583

268. Malhotra K, Ishfaq MF, Goyal N, et al. Oral anticoagulation in patients with chronic kidney disease: A systematic review and meta-analysis. Neurology 2019;92:e2421-e31. doi: https://dx.doi.org/10.1212/WNL.0000000000007534

269. Malik AH, Yandrapalli S, Aronow WS, et al. Meta-Analysis of Direct-Acting Oral Anticoagulants Compared With Warfarin in Patients >75 Years of Age. Am J Cardiol 2019;123:2051-57. doi: https://dx.doi.org/10.1016/j.amjcard.2019.02.060

270. Malik AH, Yandrapalli S, Shetty S, et al. Impact of weight on the efficacy and safety of direct-acting oral anticoagulants in patients with non-valvular atrial fibrillation: a meta-analysis. Europace 2020;22:361-67. doi: https://dx.doi.org/10.1093/europace/euz361

271. Manfredini M, Poli PP, Creminelli L, et al. Comparative risk of bleeding of anticoagulant therapy with vitamin k antagonists (Vkas) and with non-vitamin k antagonists in patients undergoing dental surgery. Journal of Clinical Medicine 2021;10(23) (no pagination) doi: http://dx.doi.org/10.3390/jcm10235526

272. Mao YJ, Wang H, Huang PF. Peri-procedural novel oral anticoagulants dosing strategy during atrial fibrillation ablation: A meta-analysis. Pacing & Clinical Electrophysiology 2020;43:1104-14. doi: https://dx.doi.org/10.1111/pace.14040

273. Mao YJ, Wang H, Huang PF. Meta-analysis of the safety and efficacy of using minimally interrupted novel oral anticoagulants in patients undergoing catheter ablation for atrial fibrillation. J Interv Card Electrophysiol 2021;60:407-17. doi: https://dx.doi.org/10.1007/s10840-020-00754-6

274. Marcucci M, Etxeandia I, Agarwal A, et al. Efficacy and safety of pharmacological thromboprophylaxis in patients undergoing noncardiac surgery: A network meta-analysis. J Urol 2020;203(Supplement 4):e175. doi: http://dx.doi.org/10.1097/JU.0000000000000836.03

275. Mariani MV, Magnocavallo M, Straito M, et al. Direct oral anticoagulants versus vitamin K antagonists in patients with atrial fibrillation and cancer a meta-analysis. Journal of Thrombosis & Thrombolysis 2021;51:419-29. doi: https://dx.doi.org/10.1007/s11239-020-02304-3

276. Marques A, Alves M, Pinto FJ, et al. The high-risk bleeding category of different scores in patients with venous thromboembolism: Systematic review and meta-analysis. European journal of internal medicine 2021;01:01. doi: https://dx.doi.org/10.1016/j.ejim.2021.10.014

277. Maughan BC, Frueh L, McDonagh MS, et al. Outpatient Treatment of Low-risk Pulmonary Embolism in the Era of Direct Oral Anticoagulants: A Systematic Review. Acad Emerg Med 2021;28:226-39. doi: https://dx.doi.org/10.1111/acem.14108

278. Meinel TR, Frey S, Arnold M, et al. Clinical presentation, diagnostic findings and management of cerebral ischemic events in patients on treatment with non-vitamin K antagonist oral anticoagulants - A systematic review. PLoS One 2019;14:e0213379. doi: https://dx.doi.org/10.1371/journal.pone.0213379

279. Meinel TR, Kniepert JU, Seiffge DJ, et al. Endovascular Stroke Treatment and Risk of Intracranial Hemorrhage in Anticoagulated Patients. Stroke 2020;51:892-98. doi: https://dx.doi.org/10.1161/STROKEAHA.119.026606

280. Mendoza PA, Narula S, McIntyre WF, et al. Continued versus interrupted direct oral anticoagulation for cardiac electronic device implantation: A systematic review. Pacing & Clinical Electrophysiology 2020;43:1373-81. doi: https://dx.doi.org/10.1111/pace.14091

281. Meng Q, Cen Z. New oral anticoagulants for nonvalvular atrial fibrillation with peripheral artery disease: a meta-analysis. Herz 2021;46:352-58. doi: https://dx.doi.org/10.1007/s00059-020-04970-8

282. Menichelli D, Del Sole F, Di Rocco A, et al. Real-world safety and efficacy of direct oral anticoagulants in atrial fibrillation: a systematic review and meta-analysis of 605 771 patients. European Heart Journal Cardiovascular Pharmacotherapy 2021;7:f11-f19. doi: https://dx.doi.org/10.1093/ehjcvp/pvab002

283. Meredith T, Schnegg B, Hayward C. The use of direct oral anticoagulants in patients with ventricular assist devices: Is there hope for Factor Xa inhibition? Artif Organs 2021;45:E123-E29. doi: https://dx.doi.org/10.1111/aor.13848

284. Mhanna M, Beran A, Al-Abdouh A, et al. Efficacy and safety of direct oral anticoagulants in morbidly obese patients with non-valvular atrial fibrillation: A systematic review and meta-analysis. Eur Heart J 2021;42(SUPPL 1):3424. doi: http://dx.doi.org/10.1093/eurheartj/ehab724.3424

285. Mhanna M, Beran A, Al-Abdouh A, et al. Direct Oral Anticoagulants Versus Warfarin in Morbidly Obese Patients With Nonvalvular Atrial Fibrillation: A Systematic Review and Meta-analysis. Am J Ther 2021;28:e531-e39. doi: https://dx.doi.org/10.1097/MJT.0000000000001403

286. Michael F, Natt N, Shurrab M. Direct Oral Anticoagulants vs Vitamin K Antagonists in Left Ventricular Thrombi: A Systematic Review and Meta-analysis. CJC Open 2021;3:1169-81. doi: https://dx.doi.org/10.1016/j.cjco.2021.04.007

287. Mincu RI, Mahabadi AA, Totzeck M, et al. Novel anticoagulants versus vitamin K antagonists for cardioversion of non- valvular atrial fibrillation - a meta-analysis of more than 17000 patients. Scientific Reports 2019;9:3011. doi: https://dx.doi.org/10.1038/s41598-019-39925-5

288. Minhas H, Welsher A, Turcotte M, et al. Incidence of intracranial bleeding in anticoagulated patients with minor head injury: a systematic review and meta-analysis of prospective studies. British Journal of Haematology 2018;183:119-26. doi: https://dx.doi.org/10.1111/bjh.15509

289. Mir T, Sattar Y, Attique HB, et al. Meta-analysis of direct oral anticoagulants compared with vitamin K antagonist for left ventricle thrombus. Cardiovascular Revascularization Medicine 2021;09:09. doi: https://dx.doi.org/10.1016/j.carrev.2021.03.001

290. Mitchell A, Watson MC, Welsh T, et al. Effectiveness and Safety of Direct Oral Anticoagulants versus Vitamin K Antagonists for People Aged 75 Years and over with Atrial Fibrillation: A Systematic Review and Meta-Analyses of Observational Studies. Journal of Clinical Medicine 2019;8:24. doi: https://dx.doi.org/10.3390/jcm8040554

291. Mitchell A, Watson MC, Welsh T, et al. A systematic review of observational studies comparing direct oral anticoagulants with Vitamin K antagonists for stroke prevention in older people with atrial fibrillation. J Pharm Pharmacol 2019;71(Supplement 1):6-7. doi: http://dx.doi.org/10.1111/jphp.13062

292. Mo Y, Yeh EJ, Yam FK. The safety of direct oral anticoagulants with P2Y12 inhibitors: A systematic review and meta-analysis of clinical trials. P and T 2019;44(10):619-27.

293. Mohan BP, Aravamudan VM, Khan SR, et al. Treatment response and bleeding events associated with anticoagulant therapy of portal vein thrombosis in cirrhotic patients: Systematic review and meta-analysis. Annals of Gastroenterology 2020;33:521-27. doi: https://dx.doi.org/10.20524/aog.2020.0503

294. Mongkhon P, Fanning L, Wong K, et al. Non-vitamin K oral anticoagulants and risk of fractures: a systematic review and meta-analysis. Europace 2021;23:39-48. doi: https://dx.doi.org/10.1093/europace/euaa242

295. Mongkhon P, Naser AY, Fanning L, et al. Oral anticoagulants and risk of dementia: A systematic review and meta-analysis of observational studies and randomized controlled trials. Neuroscience & Biobehavioral Reviews 2019;96:1-9. doi: https://dx.doi.org/10.1016/j.neubiorev.2018.10.025

296. Morrone D, Picardi G, Guarini G, et al. Intracranial haemorrhage in atrial fibrillation: A systematic review of current literature. European Heart Journal, Supplement 2020;22(SUPPL N):N10-N11. doi: http://dx.doi.org/10.1093/eurheartj/suaa190

297. Mu G, Zhang H, Liu Z, et al. Standard- vs. low-dose rivaroxaban in patients with atrial fibrillation: a systematic review and meta-analysis. Eur J Clin Pharmacol 2021;15:15. doi: https://dx.doi.org/10.1007/s00228-021-03226-6

298. Muehrcke M, Russell M, Arora P, et al. Impact of comparative effectiveness research (CER) on Part D coverage of direct oral anticoagulants (DOACs) by Centers for Medicare and Medicaid Services (CMS). Journal of Managed Care and Specialty Pharmacy 2020;26(10-A SUPPL.):S55.

299. Munawar DA, Mahajan R, Khokhar K, et al. Peri-ablation novel oral anticoagulant management: Systematic review and meta-analysis. Europace 2018;20(Supplement 1):i67-i68. doi: http://dx.doi.org/10.1093/europace/euy015

300. Murtaza G, Turagam MK, Atti V, et al. Warfarin vs non-vitamin K oral anticoagulants for left atrial appendage thrombus: A meta-analysis. J Cardiovasc Electrophysiol 2020;31:1822-27. doi: https://dx.doi.org/10.1111/jce.14502

301. Murtaza G, Turagam MK, Garg J, et al. Safety and Efficacy of Apixaban versus warfarin in patients with atrial fibrillation or Venous Thromboembolism and End-Stage renal disease on hemodialysis: A systematic review and meta-analysis. Indian Pacing & Electrophysiology Journal 2021;21:221-26. doi: https://dx.doi.org/10.1016/j.ipej.2021.04.002

302. Musat D, Garikipati N, Taub J, et al. Novel oral anticoagulants vs continuous warfarin peri-ablation of atrial fibrillation: A meta-analysis of embolic and bleeding complications. Eur Heart J 2014;1):390. doi: http://dx.doi.org/10.1093/eurheartj/ehu323

303. Nazha B, Pandya B, Cohen J, et al. Periprocedural Outcomes of Direct Oral Anticoagulants Versus Warfarin in Nonvalvular Atrial Fibrillation. Circulation 2018;138:1402-11. doi: https://dx.doi.org/10.1161/CIRCULATIONAHA.117.031457

304. Nazha B, Pandya B, Cohen J, et al. Perioperative outcomes of direct oral anticoagulants vs. Warfarin in atrial fibrillation: A Meta-analysis of Phase III Trials. Research and Practice in Thrombosis and Haemostasis 2017;1(Supplement 1):186-87. doi: http://dx.doi.org/10.1002/rth2.12012

305. Nederpelt CJ, van der Aalst SJM, Rosenthal MG, et al. Consequences of pre-injury utilization of direct oral anticoagulants in patients with traumatic brain injury: A systematic review and meta-analysis. The Journal of Trauma and Acute Care Surgery 2020;88:186-94. doi: https://dx.doi.org/10.1097/TA.0000000000002518

306. Nepal G, Kharel S, Bhagat R, et al. Safety and efficacy of Direct Oral Anticoagulants in cerebral venous thrombosis: A meta-analysis. Acta Neurol Scand 2021;21:21. doi: https://dx.doi.org/10.1111/ane.13506

307. Ng CH, Tan DJH, Nistala KRY, et al. A network meta-analysis of direct oral anticoagulants for portal vein thrombosis in cirrhosis. Hepatology International 2021;15:1196-206. doi: https://dx.doi.org/10.1007/s12072-021-10247-x

308. Ng SS, Lai NM, Nathisuwan S, et al. Comparative Efficacy And Safety of Anticoagulant Interventions In Patients With Atrial Fibrillation: A Systematic Review And Network Meta-Analysis. Value Health 2018;21(Supplement 2):S26-S27. doi: http://dx.doi.org/10.1016/j.jval.2018.07.201

309. Ng SS, Lai NM, Nathisuwan S, et al. Comparative efficacy and safety of warfarin care bundles and novel oral anticoagulants in patients with atrial fibrillation: a systematic review and network meta-analysis. Scientific Reports 2020;10:662. doi: https://dx.doi.org/10.1038/s41598-019-57370-2

310. Nilius H, Kaufmann J, Cuker A, et al. Comparative effectiveness and safety of anticoagulants for the treatment of heparin-induced thrombocytopenia. Am J Hematol 2021;96:805-15. doi: https://dx.doi.org/10.1002/ajh.26194

311. Noubiap JJ, Agbaedeng TA, Ndoadoumgue AL, et al. Atrial thrombus detection on transoesophageal echocardiography in patients with atrial fibrillation undergoing cardioversion or catheter ablation: A pooled analysis of rates and predictors. J Cardiovasc Electrophysiol 2021;32:2179-88. doi: https://dx.doi.org/10.1111/jce.15082

312. Noviyani R, Youngkong S, Nathisuwan S, et al. Economic evaluation of direct oral anticoagulants (DOACs) versus vitamin K antagonists (VKAs) for stroke prevention in patients with atrial fibrillation: a systematic review and meta-analysis. BMJ Evidence based Medicine 2021;11:11. doi: https://dx.doi.org/10.1136/bmjebm-2020-111634

313. Oh HJ, Ryu KH, Park BJ, et al. The risk of gastrointestinal hemorrhage with non-vitamin K antagonist oral anticoagulants: A network meta-analysis. Medicine 2021;100:e25216. doi: https://dx.doi.org/10.1097/MD.0000000000025216

314. Ottoffy M, Hegyi P, Habon T. Uninterrupted and minimally-interrupted direct oral anticoagulant therapy in patients undergoing catheter ablation for atrial fibrillation. An updated meta-analysis. Eur Heart J 2019;40(Supplement 1):2954. doi: http://dx.doi.org/10.1093/eurheartj/ehz745.1126

315. Ottoffy M, Matrai P, Farkas N, et al. Uninterrupted or Minimally Interrupted Direct Oral Anticoagulant Therapy is a Safe Alternative to Vitamin K Antagonists in Patients Undergoing Catheter Ablation for Atrial Fibrillation: An Updated Meta-Analysis. Journal of Clinical Medicine 2020;9:24. doi: https://dx.doi.org/10.3390/jcm9103073

316. Ottoffy M, Matrai P, Hegyi P, et al. Uninterrupted Direct Oral Anticoagulant therapy is an efficacious and safer alternative to Vitamin K Antagonists in patients undergoing catheter ablation for atrial fibrillation. A meta-analysis. Europace 2018;20(Supplement 1):i193. doi: http://dx.doi.org/10.1093/europace/euy015

317. Owen RK, Morris J, Le Reun C, et al. PCV94 Comparative Effectiveness of Direct ORAL Anticoagulants for Stroke Prevention in NON-Valvular Atrial Fibrillation. Value Health 2020;23(Supplement 2):S503. doi: http://dx.doi.org/10.1016/j.jval.2020.08.585

318. Park H, Kim JC, Cho J, et al. Systematic literature review and network meta-analysis of oral anticoagulants for the treatment of venous thromboembolism in patients with cancer. Ann Oncol 2018;29(Supplement 8):viii615. doi: http://dx.doi.org/10.1093/annonc/mdy300.039

319. Patil T, Lebrecht M. Comparison of direct oral anticoagulants versus warfarin in morbidly obese patients with venous thromboembolism: A systematic review and meta-analysis. JACCP Journal of the American College of Clinical Pharmacy 2020;3(8):1680-81. doi: http://dx.doi.org/10.1002/jac5.1351

320. Peltzer B, Lupercio F, Romero J, et al. Safety and efficacy of concomitantly used direct oral anticoagulant and amiodarone in patients with non-valvular atrial fibrillation: A meta-analysis of prospective randomized clinical trials. Journal of the American College of Cardiology Conference: 67th Annual Scientific Session of the American College of Cardiology and i2 Summit: Innovation in Intervention, ACC 2018;71 doi: http://dx.doi.org/10.1016/S0735-1097%2818%2930837-4

321. Phan K, Lloyd D, Wilson-Smith A, et al. Intraocular bleeding in patients managed with novel oral anticoagulation and traditional anticoagulation: a network meta-analysis and systematic review. Br J Ophthalmol 2018;20:20. doi: https://dx.doi.org/10.1136/bjophthalmol-2018-312198

322. Pirlog AM, Pirlog CD, Maghiar MA. DOACs vs Vitamin K Antagonists: a Comparison of Phase III Clinical Trials and a Prescriber Support Tool. Open Access Macedonian Journal of Medical Sciences 2019;7:1226-32. doi: https://dx.doi.org/10.3889/oamjms.2019.289

323. Priyanka P, Kupec JT, Krafft M, et al. Newer Oral Anticoagulants in the Treatment of Acute Portal Vein Thrombosis in Patients with and without Cirrhosis. International Journal of Hepatology 2018;2018:8432781. doi: https://dx.doi.org/10.1155/2018/8432781

324. Proietti M, Romanazzi I, Romiti GF, et al. Real-World Use of Apixaban for Stroke Prevention in Atrial Fibrillation: A Systematic Review and Meta-Analysis. Stroke 2018;49:98-106. doi: https://dx.doi.org/10.1161/STROKEAHA.117.018395

325. Puzio TJ, Murphy PB, Kregel HR, et al. Delayed Intracranial Hemorrhage after Blunt Head Trauma while on Direct Oral Anticoagulant: Systematic Review and Meta-Analysis. J Am Coll Surg 2021;232:1007-16.e5. doi: https://dx.doi.org/10.1016/j.jamcollsurg.2021.02.016

326. Qian J, Yan YD, Yang SY, et al. Benefits and Harms of Low-Dose Rivaroxaban in Asian Patients With Atrial Fibrillation: A Systematic Review and Meta-analysis of Real-World Studies. Frontiers in Pharmacology 2021;12:642907. doi: https://dx.doi.org/10.3389/fphar.2021.642907

327. Raccah B, Perlman A, Zwas DR, et al. Gender differences in efficacy and safety of direct oral anticoagulants in atrial fibrillationsystematic review and network meta-analysis. Pharmacoepidemiol Drug Saf 2018;27(Supplement 2):463. doi: http://dx.doi.org/10.1002/pds.4629

328. Raccah BH, Perlman A, Zwas DR, et al. Gender Differences in Efficacy and Safety of Direct Oral Anticoagulants in Atrial Fibrillation: Systematic Review and Network Meta-analysis. Ann Pharmacother 2018;52:1135-42. doi: https://dx.doi.org/10.1177/1060028018771264

329. Radadiya D, Devani K, Brahmbhatt B, et al. Major gastrointestinal bleeding risk with direct oral anticoagulants: Does type and dose matter? - A systematic review and network meta-analysis. Eur J Gastroenterol Hepatol 2021;09:09. doi: https://dx.doi.org/10.1097/MEG.0000000000002035

330. Rahman H, Khan SU, DePersis M, et al. Meta-analysis of safety and efficacy of oral anticoagulants in patients requiring catheter ablation for atrial fibrillation. Cardiovascular Revascularization Medicine 2019;20:147-52. doi: https://dx.doi.org/10.1016/j.carrev.2018.05.007

331. Reers S, Karanatsios G, Borowski M, et al. Frequency of atrial thrombus formation in patients with atrial fibrillation under treatment with non-vitamin K oral anticoagulants in comparison to vitamin K antagonists: a systematic review and meta-analysis. Eur J Med Res 2018;23:49. doi: https://dx.doi.org/10.1186/s40001-018-0350-9

332. Rezapour A, Souresrafil A, Arabloo J. Economic Evaluation of New Oral Anticoagulants in Prevention of Venous Thrombosis Following Joint Replacement Surgery: A Systematic Review. Clin Ther 2021;43:e139-e56. doi: https://dx.doi.org/10.1016/j.clinthera.2021.03.012

333. Romero J, Cerrud-Rodriguez RC, Alviz I, et al. Significant Benefit of Uninterrupted DOACs Versus VKA During Catheter Ablation of Atrial Fibrillation. JACC Clinical Electrophysiology 2019;5:1396-405. doi: https://dx.doi.org/10.1016/j.jacep.2019.08.010

334. Romero J, Cerrud-Rodriguez RC, Diaz JC, et al. Uninterrupted direct oral anticoagulants vs. uninterrupted vitamin K antagonists during catheter ablation of non-valvular atrial fibrillation: a systematic review and meta-analysis of randomized controlled trials. Europace 2018;20:1612-20. doi: https://dx.doi.org/10.1093/europace/euy133

335. Rossel A, Robert-Ebadi H, Combescure C, et al. Anticoagulant therapy for acute venous thrombo-embolism in cancer patients: A systematic review and network meta-analysis. PLoS One 2019;14:e0213940. doi: https://dx.doi.org/10.1371/journal.pone.0213940

336. Roule V, Ardouin P, Briet C, et al. Vitamin K antagonist vs direct oral anticoagulants with antiplatelet therapy in dual or triple therapy after percutaneous coronary intervention or acute coronary syndrome in atrial fibrillation: Meta-analysis of randomized controlled trials. Clin Cardiol 2019;09:09. doi: https://dx.doi.org/10.1002/clc.23224

337. Rujirachun P, Charoenngam N, Wattanachayakul P, et al. Efficacy and safety of direct oral anticoagulants (DOACs) versus vitamin K antagonist (VKA) among patients with atrial fibrillation and hypertrophic cardiomyopathy: A systematic review and meta-analysis. Journal of Arrhythmia 2019;35(Supplement 1):83. doi: http://dx.doi.org/10.1002/joa3.12267

338. Rujirachun P, Charoenngam N, Wattanachayakul P, et al. Efficacy and safety of direct oral anticoagulants (DOACs) versus vitamin K antagonist (VKA) among patients with atrial fibrillation and hypertrophic cardiomyopathy: a systematic review and meta-analysis. Acta Cardiol 2020;75:724-31. doi: https://dx.doi.org/10.1080/00015385.2019.1668113

339. Russo V, Bottino R, Rago A, et al. Atrial Fibrillation and Malignancy: The Clinical Performance of Non-Vitamin K Oral Anticoagulants-A Systematic Review. Semin Thromb Hemost 2019;45:205-14. doi: https://dx.doi.org/10.1055/s-0038-1661386

340. Saglietto A, D'Ascenzo F, Errigo D, et al. Antithrombotic strategies in patients needing oral anticoagulation undergoing percutaneous coronary intervention: A network meta-analysis. Catheterization & Cardiovascular Interventions 2021;97:581-88. doi: https://dx.doi.org/10.1002/ccd.29192

341. Salah HM, Goel A, Saluja P, et al. Direct Oral Anticoagulants Versus Warfarin in Left Ventricular Thrombus: A Systematic Review and Meta-Analysis. Am J Ther 2021;02:02. doi: https://dx.doi.org/10.1097/MJT.0000000000001432

342. Saleiro C, Lopes J, De Campos D, et al. Left Ventricular Thrombus Therapy With Direct Oral Anticoagulants Versus Vitamin K Antagonists: A Systematic Review and Meta-Analysis. Journal of Cardiovascular Pharmacology & Therapeutics 2021;26:233-43. doi: https://dx.doi.org/10.1177/1074248420977567

343. Samaranayake CB, Anderson J, McCabe C, et al. Direct oral anticoagulants for cancer associated venous thromboembolisms: a systematic review and network meta-analysis. Intern Med J 2020;08:08. doi: https://dx.doi.org/10.1111/imj.15049

344. Sanchez-Redondo J, Espinosa G, Varillas D, et al. Recurrent Thrombosis With Direct Oral Anticoagulants in Antiphospholipid Syndrome: A Systematic Literature Review and Meta-analysis. Clin Ther 2019;41:1839-62. doi: https://dx.doi.org/10.1016/j.clinthera.2019.06.015

345. Sanders GD, Lowenstern A, Borre E, et al. Stroke Prevention in Patients With Atrial Fibrillation: A Systematic Review Update. Agency for Healthcare Research and Quality 2018:10.

346. Santos J, Antonio N, Rocha M, et al. Impact of direct oral anticoagulant off-label doses on clinical outcomes of atrial fibrillation patients: A systematic review. British journal of clinical pharmacology 2020;86:533-47. doi: https://dx.doi.org/10.1111/bcp.14127

347. Sardar P, Chatterjee S, Herzog E, et al. New oral anticoagulants in patients with cancer: current state of evidence. Am J Ther 2015;22:460-8. doi: https://dx.doi.org/10.1097/MJT.0000000000000055

348. Sedhom R, Abdelmaseeh P, Megaly M, et al. Use of Direct Oral Anticoagulants in the Treatment of Left Ventricular Thrombi: A Systematic Review. Am J Med 2020;133:1266-73.e6. doi: https://dx.doi.org/10.1016/j.amjmed.2020.05.012

349. Sedhom R, Megaly M, Gupta E, et al. Use of direct oral anticoagulants in chronic thromboembolic pulmonary hypertension: a systematic review. Journal of Thrombosis & Thrombolysis 2021;16:16. doi: https://dx.doi.org/10.1007/s11239-021-02501-8

350. Sedhom R, Megaly M, Gupta E, et al. Use of Direct Oral Anticoagulants in Chronic Thromboembolic Pulmonary Hypertension: A Systematic Review and Meta-Analysis. J Am Coll Cardiol 2021;77(18 Supplement 1):1674. doi: http://dx.doi.org/10.1016/S0735-1097%2821%2903030-8

351. See LC, Lee HF, Chao TF, et al. Effectiveness and Safety of Direct Oral Anticoagulants in an Asian Population with Atrial Fibrillation Undergoing Dialysis: A Population-Based Cohort Study and Meta-Analysis. Cardiovascular Drugs & Therapy 2021;35:975-86. doi: https://dx.doi.org/10.1007/s10557-020-07108-4

352. Seiffge D, Goeldlin M, Tatlisumak T, et al. Meta-analysis of haematoma volume, haematoma expansion and mortality in intracerebral haemorrhage associated with oral anticoagulant use. European Stroke Journal 2019;4(Supplement 1):734. doi: http://dx.doi.org/10.1177/2396987319845581

353. Serenelli M, Vitali F, Pavasini R, et al. Novel oral anticoagulats for the treatment of left ventricle thrombosis: A systematic review and meta-analysis. Europace 2021;23(SUPPL 3):iii584. doi: http://dx.doi.org/10.1093/europace/euab116.532

354. Shah BR, Scholtus E, Rolland C, et al. A rapid evidence assessment of bleed-related healthcare resource utilization in publications reporting the use of direct oral anticoagulants for non-valvular atrial fibrillation. Curr Med Res Opin 2019;35:127-39. doi: https://dx.doi.org/10.1080/03007995.2018.1543184

355. Shah S, Shah K, Turagam MK, et al. Direct oral anticoagulants to treat left ventricular thrombus-A systematic review and meta-analysis: ELECTRAM investigators. J Cardiovasc Electrophysiol 2021;32:1764-71. doi: https://dx.doi.org/10.1111/jce.15016

356. Shahjouei S, Tsivgoulis G, Goyal N, et al. Safety of intravenous thrombolysis among patients taking direct oral anticoagulants: A systematic review and metaanalysis. Stroke Conference: American Heart Association/American Stroke Association 2018;49

357. Shahjouei S, Tsivgoulis G, Goyal N, et al. Safety of Intravenous Thrombolysis Among Patients Taking Direct Oral Anticoagulants: A Systematic Review and Meta-Analysis. Stroke 2020;51:533-41. doi: https://dx.doi.org/10.1161/STROKEAHA.119.026426

358. Shaikh F, Wynne R, Castelino RL, et al. Effectiveness of direct oral anticoagulants in obese adults with atrial fibrillation: An overview examining the evidence from international systematic reviews and meta-analyses. Eur Heart J 2021;42(SUPPL 1):2987. doi: http://dx.doi.org/10.1093/eurheartj/ehab724.2987

359. Shaw JR, Woodfine JD, Douketis J, et al. Perioperative interruption of direct oral anticoagulants in patients with atrial fibrillation: A systematic review and meta-analysis. Research And Practice In Thrombosis And Haemostasis 2018;2:282-90. doi: https://dx.doi.org/10.1002/rth2.12076

360. She J, Zhuo BZ. Meta-analysis comparing impact of age, sex and renal function on the efficacy and safety of new oral anticoagulants vs. vitamin K antagonists for the treatment of acute venous thromboembolisms. Eur Heart J 2020;41(SUPPL 2):2374. doi: http://dx.doi.org/10.1093/ehjci/ehaa946.2374

361. Shen NN, Wu Y, Wang N, et al. Direct Oral Anticoagulants vs. Vitamin-K Antagonists in the Elderly With Atrial Fibrillation: A Systematic Review Comparing Benefits and Harms Between Observational Studies and Randomized Controlled Trials. Frontiers in Cardiovascular Medicine 2020;7:132. doi: https://dx.doi.org/10.3389/fcvm.2020.00132

362. Shen NN, Zhang C, Hang Y, et al. Real-World Prevalence of Direct Oral Anticoagulant Off-Label Doses in Atrial Fibrillation: An Epidemiological Meta-Analysis. Frontiers in Pharmacology 2021;12:581293. doi: https://dx.doi.org/10.3389/fphar.2021.581293

363. Shen NN, Zhang C, Wang N, et al. Effectiveness and Safety of Under or Over-dosing of Direct Oral Anticoagulants in Atrial Fibrillation: A Systematic Review and Meta-analysis of 148909 Patients From 10 Real-World Studies. Frontiers in Pharmacology 2021;12:645479. doi: https://dx.doi.org/10.3389/fphar.2021.645479

364. Shen Y, Nie Q, Zhang Y, et al. Treatment Strategies for Cryptogenic Stroke Patients with Patent Foramen Ovale: What Do We Choose? Neuropsychiatric Disease & Treatment 2021;17:3205-14. doi: https://dx.doi.org/10.2147/NDT.S333930

365. Shurrab M, Danon A, Alnasser S, et al. Dual-Antithrombotic Therapy With DOACs After Acute Coronary Syndrome or Percutaneous Coronary Intervention in Atrial Fibrillation: A Meta-analysis of Randomized Controlled Trials. Can J Cardiol 2020;36:135-42. doi: https://dx.doi.org/10.1016/j.cjca.2019.11.005

366. Shurrab M, Danon A, Alnasser S, et al. Dual Antithrombotic Therapy with Direct-Acting Oral Anticoagulants after Acute Coronary Syndrome or Pci in Atrial Fibrillation: A Meta-Analysis of Randomized Controlled Trials. Can J Cardiol 2019;35(10 Supplement):S9-S10. doi: http://dx.doi.org/10.1016/j.cjca.2019.07.065

367. Sidahmed S, Abdalla A, Kheiri B, et al. Anticoagulants for the treatment of venous thromboembolism in patients with cancer: A comprehensive systematic review, pairwise and network meta-analysis. Critical Reviews in Oncology-Hematology 2020;152:103005. doi: https://dx.doi.org/10.1016/j.critrevonc.2020.103005

368. Siddiqui MU, Scalzitti D, Naeem Z. Apixaban in Comparison to Warfarin for Stroke Prevention in Nonvalvular Atrial Fibrillation: A Systematic Review and Meta-Analysis of Observational Studies. Cardiology Research & Practice 2019;2019:6419147. doi: https://dx.doi.org/10.1155/2019/6419147

369. Silverio A, Di Maio M, Prota C, et al. Safety and efficacy of non-vitamin K antagonist oral anticoagulants in elderly patients with atrial fibrillation: systematic review and meta-analysis of 22 studies and 440 281 patients. European Heart Journal Cardiovascular Pharmacotherapy 2021;7:f20-f29. doi: https://dx.doi.org/10.1093/ehjcvp/pvz073

370. Siontis KC, Checkole S, Yao X, et al. Do Observational Studies Agree With Randomized Trials?: Evaluation of Oral Anticoagulants in Atrial Fibrillation. J Am Coll Cardiol 2020;75(5):562-63. doi: http://dx.doi.org/10.1016/j.jacc.2019.12.007

371. Sitticharoenchai P, Takkavatakarn K, Boonyaratavej S, et al. Non-Vitamin K Antagonist Oral Anticoagulants Provide Less Adverse Renal Outcomes Than Warfarin In Non-Valvular Atrial Fibrillation: A Systematic Review and MetaAnalysis. Journal of the American Heart Association 2021;10:e019609. doi: https://dx.doi.org/10.1161/JAHA.120.019609

372. Sobieraj DM, Baker WL, Smith E, et al. Anticoagulation for the Treatment of Cancer-Associated Thrombosis: A Systematic Review and Network Meta-Analysis of Randomized Trials. Clinical & Applied Thrombosis/Hemostasis 2018;24:182S-87S. doi: https://dx.doi.org/10.1177/1076029618800792

373. Sobieraj DM, Baker WL, Smith E, et al. Anticoagulation for the treatment of cancer-associated thrombosis: A network meta-analysis of randomized trials. Circulation Conference 2018;138

374. Sohal S, Madan N, Kalra D, et al. Novel Anticoagulants or Vitamin K Antagonists for Left Ventricular Thrombus: A Meta-Analysis of Embolization Outcomes. J Am Coll Cardiol 2021;77(18 Supplement 1):771. doi: http://dx.doi.org/10.1016/S0735-1097%2821%2902130-6

375. Squizzato A, Lussana F, Cattaneo M. Post-operative arterial thrombosis with non-vitamin K antagonist oral anticoagulants after total hip or knee arthroplasty. Thromb Haemost 2015;114:237-44. doi: https://dx.doi.org/10.1160/TH15-01-0073

376. Srivastava K, Patel N, Tabbara M, et al. Thromboembolism, Bleeding, and Mortality Incidence of Direct Oral Anticoagulants Versus Warfarin Postbariatric Surgery. Am J Med 2021;134:1403-12.e2. doi: https://dx.doi.org/10.1016/j.amjmed.2021.06.021

377. Stalikas N, Doundoulakis I, Karagiannidis E, et al. Non-Vitamin K Oral Anticoagulants in Adults with Congenital Heart Disease: A Systematic Review. Journal of Clinical Medicine 2020;9:09. doi: https://dx.doi.org/10.3390/jcm9061794

378. Stalikas N, Doundoulakis I, Karagiannidis E, et al. Systematic review non-vitamin K oral anticoagulants in adults with congenital heart disease. Journal of Clinical Medicine 2020;9(6):1-14. doi: http://dx.doi.org/10.3390/jcm9061794

379. Stankovic P, Hoch S, Rudhart S, et al. Direct Oral Anticoagulants versus Vitamin K Antagonists in epistaxis patients: a systematic review and meta-analysis. Clin Otolaryngol 2021;23:23. doi: https://dx.doi.org/10.1111/coa.13898

380. Starr JA, Pinner NA, Mannis M, et al. A Review of Direct Oral Anticoagulants in Patients With Stage 5 or End-Stage Kidney Disease. Ann Pharmacother 2021:10600280211040093. doi: https://dx.doi.org/10.1177/10600280211040093

381. Su T, Fu Z, Nie Z, et al. Warfarin compared with non-vitamin K antagonist oral anticoagulants in subjects with liver disease and atrial fibrillation: A meta-analysis. Int J Clin Pract 2021;75:e14585. doi: https://dx.doi.org/10.1111/ijcp.14585

382. Su X, Yan B, Wang L, et al. Oral Anticoagulant Agents in Patients With Atrial Fibrillation and CKD: A Systematic Review and Pairwise Network Meta-analysis. Am J Kidney Dis 2021;78:678-89.e1. doi: https://dx.doi.org/10.1053/j.ajkd.2021.02.328

383. Su Z, Zhang H, He W, et al. Meta-analysis of the efficacy and safety of non-vitamin K antagonist oral anticoagulants with warfarin in Latin American patients with atrial fibrillation. Medicine 2020;99:e19542. doi: https://dx.doi.org/10.1097/MD.0000000000019542

384. Sun Y, Liu X, Xu Y. Meta-analysis of efficacy and safety of new oral anticoagulants compared with warfarin in Japanese patients undergoing catheter ablation for atrial fibrillation. J Interv Card Electrophysiol 2020;58:381-99. doi: https://dx.doi.org/10.1007/s10840-020-00784-0

385. Sun Z, Liu Y, Zhang Y, et al. Differences in safety and efficacy of oral anticoagulants in patients with non-valvular atrial fibrillation: A Bayesian analysis. Int J Clin Pract 2019;73:e13308. doi: https://dx.doi.org/10.1111/ijcp.13308

386. Sundararajan S, Poongkunran M, Poongkunran C, et al. Efficacy and bleeding risk of newer anticoagulants compared to conventional treatment in patients with chronic kidney disease-a meta-analysis of randomized controlled trials. Blood 2015;126(23):2325.

387. Tang J, Huang R, Chen Q, et al. Rivaroxaban decreases recurrent venous thromboembolisms in patients with deep vein thrombosis: A meta-analysis. International Journal of Clinical and Experimental Medicine 2018;11(10):10305-14.

388. Telles-Garcia N, Dahal K, Kocherla C, et al. Non-vitamin K antagonists oral anticoagulants are as safe and effective as warfarin for cardioversion of atrial fibrillation: A systematic review and meta-analysis. Int J Cardiol 2018;268:143-48. doi: https://dx.doi.org/10.1016/j.ijcard.2018.04.034

389. Terentes-Printzios D, Kotronias RA, De Maria GL, et al. Long-term outcomes in the management of left main disease: An updated meta-analysis of randomized controlled trials. Hellenic Journal of Cardiology 2021;62(1):87-88. doi: http://dx.doi.org/10.1016/j.hjc.2020.04.006

390. Thangjui S, Kewcharoen J, Yodsuwan R, et al. Efficacy and safety of direct oral anticoagulant in morbidly obese patients with atrial fibrillation: systematic review and meta-analysis. European Heart Journal Cardiovascular Pharmacotherapy 2021;17:17. doi: https://dx.doi.org/10.1093/ehjcvp/pvab026

391. Thomopoulos C, Ntalakouras J, Dimitris P, et al. Net clinical benefit of a reduced dose of DOACs in non-valvular atrial fibrillation: a meta-analysis of randomized trials. Pharmacol Res 2021:105902. doi: https://dx.doi.org/10.1016/j.phrs.2021.105902

392. Toso E, Peyracchia M, Matta M, et al. Incidence of thromboembolic events following atrial fibrillation catheter ablation and rate control strategies according to the kind of oral anticoagulation: A systematic review and meta-analysis. Int J Cardiol 2018;270:172-79. doi: https://dx.doi.org/10.1016/j.ijcard.2018.06.082

393. Trevisol AR, Coppi EFM, Pancotte J, et al. Use of oral anticoagulants for the prevention of thromboembolic events in the post-operative period of hip arthroplasty: a systematic review. Revista Brasileira de Ortopedia 2018;53:515-20. doi: https://dx.doi.org/10.1016/j.rboe.2018.07.005

394. Trongtorsak A, Thangjui S, Kewcharoen J, et al. Direct oral anticoagulants vs. vitamin K antagonists for left ventricular thrombus: a systematic review and meta-analysis. Acta Cardiol 2021:1-10. doi: https://dx.doi.org/10.1080/00015385.2020.1858538

395. Trujillo TC, Dobesh PP, Crossley GH, et al. Contemporary Management of Direct Oral Anticoagulants During Cardioversion and Ablation for Nonvalvular Atrial Fibrillation. Pharmacotherapy:The Journal of Human Pharmacology & Drug Therapy 2019;39:94-108. doi: https://dx.doi.org/10.1002/phar.2205

396. Tsivgoulis G, Wilson D, Katsanos AH, et al. Neuroimaging and clinical outcomes of oral anticoagulant-associated intracerebral hemorrhage. Ann Neurol 2018;84:694-704. doi: https://dx.doi.org/10.1002/ana.25342

397. Turagam MK, Osmancik P, Neuzil P, et al. Left Atrial Appendage Closure Versus Oral Anticoagulants in Atrial Fibrillation: A Meta-Analysis of Randomized Trials. J Am Coll Cardiol 2020;76(23):2795-97. doi: http://dx.doi.org/10.1016/j.jacc.2020.08.089

398. Turpin M, Gregory P. Direct Oral Anticoagulant Use and Risk of Diverticular Hemorrhage: A Systematic Review of the Literature. Canadian Journal of Gastroenterology & Hepatology 2019;2019:9851307. doi: https://dx.doi.org/10.1155/2019/9851307

399. Ueyama H, Kuno T, Ando T, et al. Meta-analysis Comparing Direct Oral Anticoagulants Versus Vitamin K Antagonists After Transcatheter Aortic Valve Implantation. Am J Cardiol 2020;125:1102-07. doi: https://dx.doi.org/10.1016/j.amjcard.2019.12.039

400. Ueyama H, Miyashita H, Takagi H, et al. Network meta-analysis of anticoagulation strategies for venous thromboembolism in patients with cancer. Journal of Thrombosis & Thrombolysis 2021;51:102-11. doi: https://dx.doi.org/10.1007/s11239-020-02151-2

401. Ueyama H, Takagi H, Briasoulis A, et al. Meta-Analysis of Antithrombotic Strategies in Patients With Heart Failure With Reduced Ejection Fraction and Sinus Rhythm. Am J Cardiol 2020;127:92-98. doi: https://dx.doi.org/10.1016/j.amjcard.2020.04.007

402. Valanejad SM, Davis KA. Direct Oral Anticoagulants in Select Patients With Hypercoagulable Disorders. Ann Pharmacother 2021;55:891-901. doi: https://dx.doi.org/10.1177/1060028020968551

403. Vasanthamohan L, Boonyawat K, Chai-Adisaksopha C, et al. Reduced-dose direct oral anticoagulants in the extended treatment of venous thromboembolism: a systematic review and meta-analysis. Journal of Thrombosis & Haemostasis 2018;16:1288-95. doi: https://dx.doi.org/10.1111/jth.14156

404. Vedovati MC, Germini F, Agnelli G, et al. Novel oral anticoagulants for patients with venous thromboembolism and active cancer: A systematic review and meta-analysis. Thromb Res 2014;2):S222.

405. Vedovati MC, Giustozzi M, Bonitta G, et al. Efficacy and safety of anticoagulant agents in patients with venous thromboembolism and cancer: A network meta-analysis. Thromb Res 2018;170:175-80. doi: https://dx.doi.org/10.1016/j.thromres.2018.08.023

406. Violi F, Vestri A, Menichelli D, et al. Direct Oral Anticoagulants in Patients With Atrial Fibrillation and Advanced Liver Disease: An Exploratory Meta-Analysis. Hepatology Communications 2020;4:1034-40. doi: https://dx.doi.org/10.1002/hep4.1513

407. Wang C, Wu Q. Efficacy and safety of non-vitamin K antagonist oral anticoagulants versus warfarin in patients with atrial fibrillation and cancer: A meta-analysis of randomized controlled trials. Journal of Arrhythmia 2019;35(Supplement 1):132-33. doi: http://dx.doi.org/10.1002/joa3.12267

408. Wang CX, Wu D, Yang PP, et al. [Efficacy and safety of non-vitamin K antagonist versus vitamin K antagonist oral anticoagulants in the prevention and treatment of thrombotic disease in active cancer patients: a systematic review and meta-analysis of randomized controlled trials]. Chung-Hua Hsin Hsueh Kuan Ping Tsa Chih [Chinese Journal of Cardiology] 2020;48:689-96. doi: https://dx.doi.org/10.3760/cma.j.cn112148-20200630-00529

409. Wang EHZ, Ye J, Turgeon R. Safety and Efficacy of Non-Vitamin K Oral Anticoagulant Use Early After Cardiac Surgery: A Systematic Review. Ann Pharmacother 2021;55:1525-35. doi: https://dx.doi.org/10.1177/10600280211006830

410. Wang KL, Chiu CC, Giugliano RP, et al. Drug Class, Renal Elimination, and Outcomes of Direct Oral Anticoagulants in Asian Patients: A Meta-Analysis. Journal of Stroke & Cerebrovascular Diseases 2018;27:857-64. doi: https://dx.doi.org/10.1016/j.jstrokecerebrovasdis.2017.10.027

411. Wang KL, Lopes RD, Patel MR, et al. Efficacy and safety of reduced-dose non-vitamin K antagonist oral anticoagulants in patients with atrial fibrillation: a meta-analysis of randomized controlled trials. Eur Heart J 2019;40:1492-500. doi: https://dx.doi.org/10.1093/eurheartj/ehy802

412. Wang KL, van Es N, Cameron C, et al. Extended treatment of venous thromboembolism: a systematic review and network meta-analysis. Heart 2019;105:545-52. doi: https://dx.doi.org/10.1136/heartjnl-2018-313617

413. Wang S, Liu Y, Wang L, et al. Optimisation of oral anticoagulants for patients with atrial fibrillation within 12months after percutaneous coronary intervention: A meta-analysis and systematic review. International Journal of Cardiology Heart & Vasculature 2021;36:100850. doi: https://dx.doi.org/10.1016/j.ijcha.2021.100850

414. Wang TF, Carrier M, Fournier K, et al. Oral anticoagulant use in patients with morbid obesity: A systemic review and meta-analysis. Research and Practice in Thrombosis and Haemostasis Conference 2021;5 doi: http://dx.doi.org/10.1002/rth2.12589

415. Wang TF, Carrier M, Fournier K, et al. Oral Anticoagulant Use in Patients with Morbid Obesity: A Systematic Review and Meta-Analysis. Thromb Haemost 2021;16:16. doi: https://dx.doi.org/10.1055/a-1588-9155

416. Wang X, Fang L, Liu B, et al. Real-world comparisons of reduced-dose non-vitamin K antagonist oral anticoagulants versus warfarin in atrial fibrillation: a systematic review and meta-analysis. Heart Failure Reviews 2020;25:973-83. doi: https://dx.doi.org/10.1007/s10741-019-09887-x

417. Wang Y, Lv H, Li D, et al. Efficacy and Safety of Direct Oral Anticoagulants for Secondary Prevention of Cancer-Associated Thrombosis: A Systematic Review and Meta-Analysis of Randomized Controlled Trials and Prospective Cohort Studies. Frontiers in Pharmacology 2019;10:773. doi: https://dx.doi.org/10.3389/fphar.2019.00773

418. Wang YP, Kehar R, Iansavitchene A, et al. Bleeding Risk in Non-Valvular Atrial Fibrillation Patients Receiving Direct Oral Anticoagulants and Warfarin: A Systematic Review and Meta-Analysis of Observational Studies. Blood 2019;134(Supplement 1):3672. doi: http://dx.doi.org/10.1182/blood-2019-123870

419. Wang YP, Kehar R, Iansavitchene A, et al. Bleeding Risk in Nonvalvular Atrial Fibrillation Patients Receiving Direct Oral Anticoagulants and Warfarin: A Systematic Review and Meta-Analysis of Observational Studies. TH Open:Companion Journal to Thrombosis and Haemostasis 2020;4:e145-e52. doi: https://dx.doi.org/10.1055/s-0040-1714918

420. Wang Z, Xiang Q, Hu K, et al. Comparison of the Safety and Efficacy of Direct Oral Anticoagulants and Warfarin in Atrial Fibrillation or Venous Thromboembolism in Patients with Renal Impairment: Systematic Review, Meta-Analysis and Network Meta-Analysis. Am J Cardiol 2021;21:643-57. doi: https://dx.doi.org/10.1007/s40256-021-00469-7

421. Waranugraha Y, Rizal A, Syaban MFR, et al. Direct comparison of non-vitamin K antagonist oral anticoagulant versus warfarin for stroke prevention in non-valvular atrial fibrillation: a systematic review and meta-analysis of real-world evidences. The Egyptian Heart Journal 2021;73:70. doi: https://dx.doi.org/10.1186/s43044-021-00194-1

422. Wartanian A, Lewinter C, Edfors R. DOAC versus warfarin in patients with atrial fibrillation and stage IV-V chronic kideny disease including patients on dialysis. Eur Heart J 2021;42(SUPPL 1):551. doi: http://dx.doi.org/10.1093/eurheartj/ehab724.0551

423. Wei AH, Gu ZC, Zhang C, et al. Increased risk of myocardial infarction with dabigatran etexilate: fact or fiction? A critical meta-analysis of over 580,000 patients from integrating randomized controlled trials and real-world studies. Int J Cardiol 2018;267:1-7. doi: https://dx.doi.org/10.1016/j.ijcard.2018.05.048

424. Wolfe Z, Khan SU, Nasir F, et al. A systematic review and Bayesian network meta-analysis of risk of intracranial hemorrhage with direct oral anticoagulants. Journal of Thrombosis & Haemostasis 2018;16:1296-306. doi: https://dx.doi.org/10.1111/jth.14131

425. Wolfe Z, Nasir F, Subramanian C, et al. Risk of intracranial hemorrhage with direct oral anticoagulants: A traditional and network meta-analysis. Blood Conference: 59th Annual Meeting of the American Society of Hematology, ASH 2017;130

426. Wu T, Lv C, Wu L, et al. Risk of intracranial hemorrhage with direct oral anticoagulants: a systematic review and meta-analysis of randomized controlled trials. J Neurol 2021;17:17. doi: https://dx.doi.org/10.1007/s00415-021-10448-2

427. Wu X, Hu L, Liu J, et al. Association of Direct Oral Anticoagulants vs. Vitamin K Antagonists With Fractures in Atrial Fibrillation Patients: A Systematic Review and Meta-Analysis. Frontiers in Cardiovascular Medicine 2021;8:713187. doi: https://dx.doi.org/10.3389/fcvm.2021.713187

428. Xin Z, Liu F, Du Y, et al. Primary prophylaxis for venous thromboembolism in ambulatory cancer patients: a systematic review and network meta-analysis. Annals of Palliative Medicine 2020;9:2970-81. doi: https://dx.doi.org/10.21037/apm-20-47

429. Xiong Q, Wang C, Liu H, et al. Efficacy and Safety of Non-Vitamin K Antagonist Oral Anticoagulants in Asians With Nonvalvular Atrial Fibrillation: A Network Meta-Analysis. Clinical & Applied Thrombosis/Hemostasis 2019;25:1076029619885188. doi: https://dx.doi.org/10.1177/1076029619885188

430. Xu M, Xue Q, Pu Z, et al. Efficacy and safety of novel oral anticoagulants versus vitamin K antagonists in the treatment of venous thromboembolism. Bangladesh Journal of Pharmacology 2018;13(3):273-79. doi: http://dx.doi.org/10.3329/bjp.v13i3.37124

431. Xu R, Wu F, Lan J, et al. Real-world comparison of direct-acting oral anticoagulants and vitamin K antagonists in chronic kidney disease: a systematic review and meta-analysis. Expert Review of Hematology 2021;14:493-502. doi: https://dx.doi.org/10.1080/17474086.2021.1920012

432. Xu W, Lv M, Wu S, et al. Severe Bleeding Risk of Direct Oral Anticoagulants Versus Vitamin K Antagonists for Stroke Prevention and Treatment in Patients with Atrial Fibrillation: A Systematic Review and Network Meta-Analysis. Cardiovascular Drugs & Therapy 2021;26:26. doi: https://dx.doi.org/10.1007/s10557-021-07232-9

433. Xu Y, You D, Ponich B, et al. Effect of oral anticoagulant use on surgical delay and outcomes in hip fracture patients: A systematic review and meta-analysis. Research and Practice in Thrombosis and Haemostasis 2020;4(SUPPL 1):564-65. doi: http://dx.doi.org/10.1002/rth2.12393

434. Xuan H, Chen YM, Dai YL, et al. Safety and Efficacy of Vitamin K Antagonists vs. Novel Oral Anticoagulants in Patients With Left Ventricular Thrombus: A Meta-Analysis. Frontiers in Cardiovascular Medicine 2021;8:636491. doi: https://dx.doi.org/10.3389/fcvm.2021.636491

435. Xue Z, Zhang H. Non-Vitamin K Antagonist Oral Anticoagulants Versus Warfarin in Asians With Atrial Fibrillation: Meta-Analysis of Randomized Trials and Real-World Studies. Stroke 2019;50:2819-28. doi: https://dx.doi.org/10.1161/STROKEAHA.119.026054

436. Xue Z, Zhou Y, Wu C, et al. Non-vitamin K antagonist oral anticoagulants in Asian patients with atrial fibrillation: evidences from the real-world data. Heart Failure Reviews 2020;25:957-64. doi: https://dx.doi.org/10.1007/s10741-019-09878-y

437. Yamashita Y, Morimoto T, Toyota T, et al. Asian patients versus non-Asian patients in the efficacy and safety of direct oral anticoagulants relative to vitamin K antagonist for venous thromboembolism: A systemic review and meta-analysis. Thromb Res 2018;166:37-42. doi: https://dx.doi.org/10.1016/j.thromres.2018.04.008

438. Yamashita Y, Morimoto T, Toyota T, et al. Asian patients versus non-Asian patients in the efficacy and safety of direct oral anticoagulants relative to vitamin K antagonist for venous thromboembolism: A systemic review and meta-analysis. Eur Heart J 2018;39(Supplement 1):867. doi: http://dx.doi.org/10.1093/eurheartj/ehy563.4319

439. Yan YD, Ding Z, Pan MM, et al. Net Clinical Benefit of Direct Oral Anticoagulants in Patients With Cancer and Venous Thromboembolism: A Systematic Review and Trade-Off Analysis. Frontiers in Cardiovascular Medicine 2020;7:586020. doi: https://dx.doi.org/10.3389/fcvm.2020.586020

440. Yan YD, Zhang C, Shen L, et al. Net Clinical Benefit of Non-vitamin K Antagonist Oral Anticoagulants for Venous Thromboembolism Prophylaxis in Patients With Cancer: A Systematic Review and Trade-Off Analysis From 9 Randomized Controlled Trials. Frontiers in Pharmacology 2018;9:575. doi: https://dx.doi.org/10.3389/fphar.2018.00575

441. Yang J, Zhang X, Wang XY, et al. Comparison of transesophageal echocardiography findings after different anticoagulation strategies in patients with atrial fibrillation: a systematic review and meta-analysis. BMC Cardiovasc Disord 2019;19:261. doi: https://dx.doi.org/10.1186/s12872-019-1209-x

442. Yang KT, Sun WC, Tsai TJ, et al. The Risk of Gastrointestinal Bleeding between Non-Vitamin K Antagonist Oral Anticoagulants and Vitamin K Antagonists in the Asian Atrial Fibrillation Patients: A Meta-Analysis. International Journal of Environmental Research & Public Health [Electronic Resource] 2020;18:27. doi: https://dx.doi.org/10.3390/ijerph18010137

443. Yang P, Wang C, Ye Y, et al. Interrupted or Uninterrupted Oral Anticoagulants in Patients Undergoing Atrial Fibrillation Ablation. Cardiovascular Drugs & Therapy 2020;34:371-81. doi: https://dx.doi.org/10.1007/s10557-020-06967-1

444. Yang P, Zhu D, Xu X, et al. Efficacy and safety of oral anticoagulants in atrial fibrillation patients with cancer-a network meta-analysis. Heart Failure Reviews 2020;25:823-31. doi: https://dx.doi.org/10.1007/s10741-019-09844-8

445. Yang Q, Chen X, Zhai J, et al. Comparison of major bleeding events of uninterrupted non-vitamin K antagonist oral anticoagulants versus uninterrupted vitamin K antagonist during catheter ablation of atrial fibrillation: a meta-analysis of randomised controlled trials. Acta Cardiol 2021:1-7. doi: https://dx.doi.org/10.1080/00015385.2021.1915603

446. Yu YB, Liu J, Fu GH, et al. Comparison of dabigatran and warfarin used in patients with non-valvular atrial fibrillation: Meta-analysis of random control trial. Medicine 2018;97:e12841. doi: https://dx.doi.org/10.1097/MD.0000000000012841

447. Yu Z, Shan P, Yang X, et al. Comparison of efficiency and safety of rivaroxaban, apixaban and enoxaparin for thromboprophylaxis after arthroplastic surgery: a meta-analysis. Biosci Rep 2018;38:21. doi: https://dx.doi.org/10.1042/BSR20180423

448. Zakko J, Ganapathi AM, Whitson BA, et al. Safety of direct oral anticoagulants in solid organ transplant recipients: A meta-analysis. Clin Transplant 2021:e14513. doi: https://dx.doi.org/10.1111/ctr.14513

449. Zeng J, Zhang X, Lip GYH, et al. Efficacy and Safety of Direct Oral Anticoagulants for Risk of Cancer-Associated Venous Thromboembolism. Clinical & Applied Thrombosis/Hemostasis 2019;25:1076029619853629. doi: https://dx.doi.org/10.1177/1076029619853629

450. Zhang C, Gu ZC, Ding Z, et al. Decreased risk of renal impairment in atrial fibrillation patients receiving non-vitamin K antagonist oral anticoagulants: A pooled analysis of randomized controlled trials and real-world studies. Thromb Res 2019;174:16-23. doi: https://dx.doi.org/10.1016/j.thromres.2018.12.010

451. Zhang C, Gu ZC, Shen L, et al. Non-vitamin K Antagonist Oral Anticoagulants and Cognitive Impairment in Atrial Fibrillation: Insights From the Meta-Analysis of Over 90,000 Patients of Randomized Controlled Trials and Real-World Studies. Frontiers in aging neuroscience 2018;10:258. doi: https://dx.doi.org/10.3389/fnagi.2018.00258

452. Zhang C, Wang N, Shen NN, et al. Net clinical benefit of antithrombotic therapy in patients with heart failure and sinus rhythm: A network meta-analysis from 5 clinical trials. Thromb Res 2020;190:122-28. doi: https://dx.doi.org/10.1016/j.thromres.2020.04.017

453. Zhang H, Xue Z, Yi D, et al. Non-Vitamin K Antagonist Oral Anticoagulants Versus Warfarin in Patients with Atrial Fibrillation with Coronary or Peripheral Artery Disease. International Heart Journal 2020;61:231-38. doi: https://dx.doi.org/10.1536/ihj.19-202

454. Zhang J, Tang J, Cui X, et al. Indirect comparison of novel Oral anticoagulants among Asians with non-Valvular atrial fibrillation in the real world setting: a network meta-analysis. BMC Cardiovasc Disord 2019;19:182. doi: https://dx.doi.org/10.1186/s12872-019-1165-5

455. Zhang J, Wang X, Liu X, et al. Comparative effectiveness and safety of direct acting oral anticoagulants in nonvalvular atrial fibrillation for stroke prevention: a systematic review and meta-analysis. Eur J Epidemiol 2021;36:793-812. doi: https://dx.doi.org/10.1007/s10654-021-00751-7

456. Zhao X, Huang Y, Li J, et al. Management of anticoagulants in delayed bleeding after endoscopic resection: A systematic review and meta-analysis. Endoscopy International Open 2021;9:E1128-E35. doi: https://dx.doi.org/10.1055/a-1467-6068

457. Zhao Y, Fang Z, Liu L, et al. The optimal anticoagulation during catheter ablation of atrial fibrillation: A systematic review and network meta-analysis. British Journal of Haematology 2020;189(Supplement 1):157-58. doi: http://dx.doi.org/10.1111/bjh.16638

458. Zhao Y, Lu Y, Qin Y. A meta-analysis of randomized controlled trials of uninterrupted periprocedural anticoagulation strategy in patients undergoing atrial fibrillation catheter ablation. Int J Cardiol 2018;270:167-71. doi: https://dx.doi.org/10.1016/j.ijcard.2018.06.024

459. Zheng Y, Liu Y, Bi J, et al. Novel Oral Anticoagulants for the Prevention of Stroke in Patients with Atrial Fibrillation and Hypertension: A Meta-Analysis. Am J Cardiol 2019;19:477-85. doi: https://dx.doi.org/10.1007/s40256-019-00342-8

460. Zhou B, Wu H, Wang C, et al. Impact of Age, Sex, and Renal Function on the Efficacy and Safety of Direct Oral Anticoagulants vs. Vitamin K Antagonists for the Treatment of Acute Venous Thromboembolism: A Meta-Analysis of 22,040 Patients. Frontiers in Cardiovascular Medicine 2021;8:700740. doi: https://dx.doi.org/10.3389/fcvm.2021.700740

461. Zhou K, Zhang X, Xiao Y, et al. Effectiveness and safety of direct-acting oral anticoagulants compared to vitamin K antagonists in patients with left ventricular thrombus: A meta-analysis. Thromb Res 2021;197:185-91. doi: https://dx.doi.org/10.1016/j.thromres.2020.11.018

462. Zhou LY, Yang SF, Zhang Z, et al. A Renal Function Based Trade-Off Analysis of Non-vitamin K Antagonist Oral Anticoagulants in Nonvalvular Atrial Fibrillation. Frontiers in Physiology 2018;9:1644. doi: https://dx.doi.org/10.3389/fphys.2018.01644

463. Zhou Y, He W, Zhou Y, et al. Non-vitamin K antagonist oral anticoagulants in patients with hypertrophic cardiomyopathy and atrial fibrillation: a systematic review and meta-analysis. Journal of Thrombosis & Thrombolysis 2020;50:311-17. doi: https://dx.doi.org/10.1007/s11239-019-02008-3

464. Zhou Y, Ma J, Zhu W. Efficacy and Safety of Direct Oral Anticoagulants Versus Warfarin in Patients with Atrial Fibrillation Across BMI Categories: A Systematic Review and Meta-Analysis. Am J Cardiol 2020;20:51-60. doi: https://dx.doi.org/10.1007/s40256-019-00362-4

465. Zhou Y, Yao Z, Zhu L, et al. Safety of Dabigatran as an Anticoagulant: A Systematic Review and Meta-Analysis. Frontiers in Pharmacology 2021;12:626063. doi: https://dx.doi.org/10.3389/fphar.2021.626063

466. Zhu H, Li F, Tao K, et al. Bleeding after endoscopic resection between direct oral anticoagulants or warfarin: Systematic review and meta-analysis. J Gastroenterol Hepatol 2021;36:2363-74. doi: https://dx.doi.org/10.1111/jgh.15527

467. Zhu W, Ye Z, Chen S, et al. Comparative Effectiveness and Safety of Non-Vitamin K Antagonist Oral Anticoagulants in Atrial Fibrillation Patients. Stroke 2021;52:1225-33. doi: https://dx.doi.org/10.1161/STROKEAHA.120.031007

468. Zhuang Y, Dai LF, Chen MQ. Efficacy and safety of non-vitamin K antagonist oral anticoagulants for venous thromboembolism: a meta-analysis. JRSM Open 2021;12:20542704211010686. doi: https://dx.doi.org/10.1177/20542704211010686
