# Supplementary material for: Oral anticoagulants: a systematic overview of reviews on efficacy and safety, genotyping, self-monitoring, and stakeholder experiences
Source: Syst Rev. 2022 Oct 28;11:232. doi: 10.1186/s13643-022-02098-w (PMC9615370; doi:10.1186/s13643-022-02098-w)
Supplement: Supplementary file 6 — Additional file 6. Articles excluded at full-text assessment. [file 13643_2022_2098_MOESM6_ESM.docx]

Additional file 6: Articles excluded at full-text assessment

***Efficacy and safety***:

***Original search***: 425 papers were identified as potentially relevant based on title and abstract; one review was included (Sterne et al., 2017) and the other 424 were not assessed (see Additional file on the EPPI-Centre website).

***Update:*** 468 papers were identified as potentially relevant (see Additional file 5).

***Genotyping***:

***Original search****:* 22 reviews were assessed on the full text, and 12 were excluded. The reasons for exclusion were:

*Conference Abstracts (two):*

Belley-Cote E P, Hanif H, D'Aragon F, Eikelboom J, Anderson J L, Borgman M, Jonas D E, Kimmel S, Maitland-Van Der Zee A H, Pirmohamed M, and Whitlock R. (2014). Genotype-guided vitamin K antagonist dosing algorithms improve time in therapeutic range: A systematic review and meta-analysis. Circulation. Conference: American Heart Association's, 130, pp.

Smith S A. (2015). Systematic review of recent pharmacoeconomic evaluations related to genotype-guided therapy in patients at high risk for thrombotic event. Value in Health, 18(3), pp.A142.

*Not focussed on AF or VTE (seven):*

Belley-Cote E P, Hanif H, D'Aragon F, Eikelboom J W, Anderson J L, Borgman M, Jonas D E, Kimmel S E, Manolopoulos V G, Baranova E, Maitland-van der Zee, A H, Pirmohamed M, and Whitlock R P. (2015). Genotype-guided versus standard vitamin K antagonist dosing algorithms in patients initiating anticoagulation. A systematic review and meta-analysis. Thrombosis & Haemostasis, 114, pp.768-77.

Liu H Q, Zhang C P, Zhang C Z, Liu X C, and Liu Z J. (2015). Influence of two common polymorphisms in the EPHX1 gene on warfarin maintenance dosage: a meta-analysis. BioMed Research International, 2015, pp.564149.

Plumpton C O, Roberts D, Pirmohamed M, and Hughes D A. (2016). A systematic review of economic evaluations of pharmacogenetic testing for prevention of adverse drug reactions. PharmacoEconomics, 34, pp.771-793.

Stergiopoulos K, and Brown D L. (2014). Genotype-guided vs clinical dosing of warfarin and its analogues: meta-analysis of randomized clinical trials. JAMA Internal Medicine, 174, pp.1330-8.

Sun Y, Wu Z, Li S, Qin X, Li T, Xie L, Deng Y, and Chen J. (2015). Impact of gamma-glutamyl carboxylase gene polymorphisms on warfarin dose requirement: a systematic review and meta-analysis. Thrombosis Research, 135, pp.739-47.

Tang H L, Shi W L, Li X G, Zhang T, Zhai S D, and Xie H G. (2015). Limited clinical utility of genotype-guided warfarin initiation dosing algorithms versus standard therapy: a meta-analysis and trial sequential analysis of 11 randomized controlled trials. Pharmacogenomics Journal, 15, pp.496-504.

Tang W, Shi Q P, Ding F, Yu M L, Hua J, and Wang Y X. (2017). Impact of VKORC1 gene polymorphisms on warfarin maintenance dosage: A novel systematic review and meta-analysis of 53 studies. International Journal of Clinical Pharmacology & Therapeutics, 55, pp.304-321

*Not a systematic review (two):*

Dahal K, Sharma S, and Lee J. (2014). A meta-analysis of randomized trials of genotype-guided versus standard dosing of warfarin. European Heart Journal, 35, pp.381-382.

Liao Z, Feng S, Ling P, and Zhang G. (2015). Meta-analysis of randomized controlled trials reveals an improved clinical outcome of using genotype plus clinical algorithm for warfarin dosing. Journal of Thrombosis & Thrombolysis, 39, pp.228-34.

*No health or cost outcomes (one):*

Martin A, Downing J, Maden M, Fleeman N, Alfirevic A, Haycox A, and Pirmohamed M. (2017). An assessment of the impact of pharmacogenomics on health disparities: a systematic literature review. Pharmacogenomics, 18, pp.1541-1550.

***Self-monitoring:***

***Original search***: 20 reviews were assessed on the full text, two were included for stakeholder experiences only, not self-monitoring,^1 2^ and 12 were excluded. The reasons for exclusion were:

*Not focussed on self-monitoring (four):*

Abusanad OZ, Floyd MS, Johnson EU, McHugh J, McCabe JE. Haematological considerations in urology: A systematic review. Journal of Clinical Urology. 2015;8:321-8.

Caldeira D, Goncalves N, Ferreira JJ, Pinto FJ, Costa J. Tolerability and acceptability of non-vitamin K antagonist oral anticoagulants in atrial fibrillation: systematic review and meta-analysis. American Journal of Cardiovascular Drugs. 2015;15:259-65.

Darnell SW, Davis SC, Whitcomb JJ, Manfredi JA, McLaurin BT. Bleeding risk factors affecting warfarin therapy in the elderly with atrial fibrillation. DCCN - Dimensions of Critical Care Nursing. 2014;33:57-63.

Ganji R, Ala S, Aarabi M, Baghery B, Salehifar E. Comparison of dabigatran vs. warfarin in acute venous thromboemboly: systematic review. Iranian Journal of Pharmaceutical Research. 2016;15:611-7.

*Not a systematic review (seven):*

Bhagavathula AS, Shehab A, Abegaz TM, Befekadu T. Patient adherence to novel oral anticoagulants (NOACs) for the treatment of atrial fibrillation and occurrence of associated bleeding events: A systematic review and meta-analysis. Value in Health. 2017;20 (5):A264.

Bussey HI, Nutescu E. Assessment of a new method of warfarin management vs the new oral anticoagulants and conventional warfarin management in atrial fibrillation. Stroke Conference: American Heart Association/American Stroke Association. 2015;46.

Cheng WH, Manzoor BS, Cavallari L, Sharp L, Gerber B, Fitzgibbon ML, et al. Quality of pharmacist-managed anticoagulation therapy in long-term ambulatory settings: A systematic review. Value in Health. 2014;17 (3):A126-A7.

Komocsi A. Discontinuation of anticoagulant treatment: From clinical trials to medication persistence. Current Medical Research and Opinion. 2015;31:1841-4.

Locatelli I, Marjanovic I, Janzic A. Systematic Review and Meta-Analysis of Self-Monitoring and Self-Management of Anticoagulation Therapy with Vitamin K Antagonists. Value in Health. 2015;18:A377.

McHorney CA, Peterson E, Ashton V, Laliberte F, Crivera C, Sheikh N, et al. Adherence to QD vs. BID medications in NVAF patients is associated with reduced risk of ischemic stroke: A modeling study using RCT and claims data. Circulation Conference: American Heart Association's. 2016;134.

Solomon Jenna M, Hume Anne L. Direct Oral Anticoagulants: A Patient-Centered Review. The Journal for Nurse Practitioners. 2016;12:523-9.

*Not in English (one):*

Simonetti Sérgio H, Mancussi e F, Ana C, Ferraz B, Estela R. Adherence to therapy with oral anticoagulants: an integrative review. Journal of Nursing UFPE / Revista de Enfermagem UFPE. 2014;8:2854-63.

***Stakeholder experiences:***

***Original search***: 11 articles were assessed on full text, and five were excluded. Three articles included for self-monitoring were also included in stakeholder experiences.^3-5^ The reasons for exclusion were:

*Not focussed on experiences (three):*

*Caldeira D, Goncalves N, Ferreira JJ, Pinto FJ, Costa J. Tolerability and Acceptability of Non-Vitamin K Antagonist Oral Anticoagulants in Atrial Fibrillation: Systematic Review and Meta-Analysis. American Journal of Cardiovascular Drugs. 2015;15:259-65.

*Ganji R, Ala S, Aarabi M, Baghery B, Salehifar E. Comparison of Dabigatran vs. Warfarin in Acute Venous Thromboemboly: Systematic Review. Iranian Journal of Pharmaceutical Research. 2016;15:611-7.

Metaxas C, Wentzky V, Habegger S, Hersberger KE, Arnet I. Development of an instrument to assess patient knowledge about therapy with new oral anticoagulants in view of providing targeted education. International Journal of Clinical Pharmacy. 2016;38 (4):1008.

*Not a systematic review (two):*

Loewen P, Ji A, Kapanen A. Patient values and preferences for antithrombotic therapy for stroke prevention in atrial fibrillation: A systematic review. Canadian Journal of Cardiology. 2016;32 (10 Supplement 1):S257-S8.

Oqab Z, Pournazari P, Sheldon R. Frailty assessment and atrial fibrillation: A systematic review and meta-analysis of prevalence and effect on prescription of anticoagulation. Canadian Journal of Cardiology. 2016;32 (10 Supplement 1):S209-S10.

*Caldeira and Ganji were also excluded from self-monitoring

***General:***

*Original search:* two articles were not assigned to a topic and were excluded.

*Not a systematic review (two):*

Abegaz TM, Tamrat B, Bahagavathula A, et al. Novel oral anticoagulants (NOACS) adherence and bleeding events in atrial fibrillation patients: A systematic review and meta-analysis. *Value Health* 2017;20 (5):A272-A73.

Buddam A, Kanmanthareddy A, Dacha S, et al. Gastrointestinal bleeding with warfarin, novel oral anticoagulants and left atrial appendage exclusion device in patients with atrial fibrillation: Network meta-analysis of randomized controlled trials. *Gastrointest Endosc* 2017;85 (5 Supplement 1):AB297.

***Update articles excluded on full-text assessment:*** *43* articles were assessed on full text, and 16 articles were excluded. The reasons for exclusion were:

*Not a systematic review (seven):*

Gebreyohannes Eyob A, Salter S, Chalmers L, et al. Non-adherence to Thromboprophylaxis Guidelines in Atrial Fibrillation: A Narrative Review of the Extent of and Factors in Guideline Non-adherence. *Am J Cardiol* 2021;21:419-33. doi: 10.1007/s40256-020-00457-3

Hori M, Tanahashi N, Akiyama S, et al. Cost-effectiveness of rivaroxaban versus warfarin for stroke prevention in non-valvular atrial fibrillation in the Japanese healthcare setting. *J Med Econ* 2020;23:252-61. doi: https://dx.doi.org/10.1080/13696998.2019.1688821

Kefale AT, Peterson GM, Bezabhe WM, et al. Switching of oral anticoagulants in patients with nonvalvular atrial fibrillation: A narrative review. *British journal of clinical pharmacology* 2021;05:05. doi: https://dx.doi.org/10.1111/bcp.15021

Lee T, Davis E, Kielly J. Clinical impact of a pharmacist-led inpatient anticoagulation service: a review of the literature. *Integrated Pharmacy Research & Practice* 2016;5:53-63. doi: https://dx.doi.org/10.2147/IPRP.S93312

Mohan A, Wanat MA, Abughosh SM. Medication taking behaviors in patients taking warfarin versus direct oral anticoagulants: A systematic review. *Expert Review of Cardiovascular Therapy* 2019;17:427-34. doi: https://dx.doi.org/10.1080/14779072.2019.1620600

Raymond J, Imbert L, Cousin T, et al. Pharmacogenetics of Direct Oral Anticoagulants: A Systematic Review. *Journal of Personalized Medicine* 2021;11:11. doi: https://dx.doi.org/10.3390/jpm11010037

Schelde Astrid B, Eliasen A, Olesen Jonas B, et al. Real-world effectiveness and safety of pharmacological thromboprophylaxis in patients undergoing primary total hip and knee arthroplasty: A narrative review. *Journal of Orthopaedics* 2020;19:166-73. doi: 10.1016/j.jor.2019.11.012

*Not focussed on AF or VTE (five):*

Cabellos-Garcia AC, Martinez-Sabater A, Castro-Sanchez E, et al. Relation between health literacy, self-care and adherence to treatment with oral anticoagulants in adults: a narrative systematic review. BMC Public Health 2018;18:1157. doi: https://dx.doi.org/10.1186/s12889-018-6070-9

Dai H, Zheng C, Lin C, et al. Technology-Based Interventions in Oral Anticoagulation Management: Meta-Analysis of Randomized Controlled Trials. Journal of Medical Internet Research 2020;22:e18386. doi: https://dx.doi.org/10.2196/18386

Danese E, Raimondi S, Montagnana M, et al. Effect of CYP4F2, VKORC1, and CYP2C9 in Influencing Coumarin Dose: A Single-Patient Data Meta-Analysis in More Than 15,000 Individuals. Clin Pharmacol Ther 2019;105(6):1477-91. doi: http://dx.doi.org/10.1002/cpt.1323

Lee M, Wang M, Liu J, et al. Do telehealth interventions improve oral anticoagulation management? A systematic review and meta-analysis. Journal of Thrombosis & Thrombolysis 2018;45:325-36. doi: https://dx.doi.org/10.1007/s11239-018-1609-2

Yiu A, Bajorek B. Patient-focused interventions to support vulnerable people using oral anticoagulants: a narrative review. Therapeutic Advances in Drug Safety 2019;10:2042098619847423. doi: <https://dx.doi.org/10.1177/2042098619847423>

*Not focussed on adherence (self-monitoring), genotyping, or experiences (three):*

Pandya E, Bajorek BV. Assessment of Web-based education resources informing patients about stroke prevention in atrial fibrillation. *J Clin Pharm Ther* 2016;41:667-76. doi: 10.1111/jcpt.12446

Sennesael AL, Krug B, Sneyers B, et al. Do computerized clinical decision support systems improve the prescribing of oral anticoagulants? A systematic review. *Thromb Res* 2020;187:79-87. doi: https://dx.doi.org/10.1016/j.thromres.2019.12.023

Trocio J, Rosen VM, Gupta A, et al. Systematic literature review of treatment patterns for venous thromboembolism patients during transitions from inpatient to post-discharge settings. *Clinicoeconomics & Outcomes Research* 2019;11:23-49. doi: https://dx.doi.org/10.2147/CEOR.S179080

*No health or cost outcomes (one):*

Gebreyohannes EA, Mill D, Salter S, et al. Strategies for improving guideline adherence of anticoagulants for patients with atrial fibrillation in primary healthcare: A systematic review. Thromb Res 2021;205:128-36. doi: https://dx.doi.org/10.1016/j.thromres.2021.07.014

**References**

1. Alamneh EA, Chalmers L, Bereznicki LR. Suboptimal use of oral anticoagulants in atrial fibrillation: has the introduction of direct oral anticoagulants improved prescribing practices? *Am J Cardiovasc Drugs* 2016;16(3):183-200. doi: 10.1007/s40256-016-0161-8 [published Online First: 2016/02/11]

2. Willett KC, Morrill AM. Use of direct oral anticoagulants for the prevention and treatment of thromboembolic disease in patients with reduced renal function: a short review of the clinical evidence. *Ther Clin Risk Manag* 2017;13:447-54. doi: 10.2147/tcrm.S88911 [published Online First: 2017/04/25]

3. Clarkesmith DE, Pattison HM, Khaing PH, et al. Educational and behavioural interventions for anticoagulant therapy in patients with atrial fibrillation. *Cochrane Database of Systematic Reviews* 2017;4:Cd008600. doi: 10.1002/14651858.CD008600.pub3 [published Online First: 2017/04/06]

4. Entezari-Maleki T, Dousti S, Hamishehkar H, et al. A systematic review on comparing 2 common models for management of warfarin therapy; pharmacist-led service versus usual medical care. *J Clin Pharmacol* 2016;56(1):24-38. doi: 10.1002/jcph.576 [published Online First: 2015/06/24]

5. Zhou S, Sheng XY, Xiang Q, et al. Comparing the effectiveness of pharmacist-managed warfarin anticoagulation with other models: a systematic review and meta-analysis. *J Clin Pharm Ther* 2016;41(6):602-11. doi: 10.1111/jcpt.12438 [published Online First: 2016/10/28]
