# Supplementary material for: Oral anticoagulants: a systematic overview of reviews on efficacy and safety, genotyping, self-monitoring, and stakeholder experiences
Source: Syst Rev. 2022 Oct 28;11:232. doi: 10.1186/s13643-022-02098-w (PMC9615370; doi:10.1186/s13643-022-02098-w)
Supplement: Supplementary file 7 — Additional file 7. Quality assessment of the included reviews for efficacy and safety, genotyping, and self-monitoring. [file 13643_2022_2098_MOESM7_ESM.docx]

Additional file 7: Quality assessment of the included reviews for efficacy and safety, genotyping, and self-monitoring

|  | **1** | **2** | **3** | **4** | **5** | **6** | **7** | **8** | **9a** | **9b** | **10** | **11a** | **11b** | **12** | **13** | **14** | **15** | **16** |
| --- | --- | --- | --- | --- | --- | --- | --- | --- | --- | --- | --- | --- | --- | --- | --- | --- | --- | --- |
| First author (year) | Include PICO? | Protocol? | Inclusion criteria | Comprehensive search strategy? | Duplicate screening? | Duplicate DE? | Exclusions? | Included described in detail? | RCTs - RoB assessment? | NRSI - RoB assessment? | Funding stated? | RCTs: Appropriate meta-analysis methods? | NRSI: Appropriate meta-analysis methods? | RoB impact on meta-analysis? | RoB in interpretation of results? | Heterogeneity explanation/discussion? | Publication bias? | Conflict of interest stated? |
| **Efficacy and safety reviews** | | | | | | | | | | | | | | | | | | |
| Sterne (2017)  Review 1 | + | PY^[[1]](#footnote-1)^ | + | PY^[[2]](#footnote-2)^ | + | + | - | + | + | NA | + | + | NA | + | PY^[[3]](#footnote-3)c^ | + | - | + |
| Sterne (2017)  Review 2 | + | PY^1^ | + | PY^2^ | + | + | - | + | + | NA | + | + | NA | + | PY^3^ | + | - | + |
| Sterne (2017)  Review 3 | + | PY^1^ | + | PY^2^ | + | + | - | + | + | NA | + | + | NA | + | PY^3^ | + | - | + |
| Sterne (2017)  Review 4 | + | PY1 | + | PY^2^ | + | + | - | + | + | NA | + | + | NA | + | PY^3^ | + | - | + |
| **Genotyping reviews** | | | | | | | | | | | | | | | | | | |
| Asiimwe (2020) | + | + | - | + | + | - | - | + | PY | PY | - | + | + | - | + | + | + | - |
| Chen (2016) | + | - | - | - | + | - | - | PY | NA | + | - | NA | + | + | + | + | - | + |
| Dahal (2015) | + | PY | - | + | - | - | - | PY | PY | NA | - | + | NA | + | + | + | + | + |
| Franchini (2014) | + | PY | - | + | + | + | - | PY | + | NA | - | + | NA | - | - | - | - | + |
| Goulding (2015) | + | - | - | PY | + | + | - | + | + | NA | - | + | NA | - | - | - | - | + |
| Jin (2014) | - | - | - | - | - | + | - | PY | NA | - | - | NA | - | - | - | - | + | + |
| Kheiri (2018) | - | - | + | PY | + | + | - | + | - | NA | - | + | NA | - | - | + | + | + |
| Ng (2020) | + | + | - | PY | - | + | - | + | + | NA | + | + | NA | + | + | + | + | + |
| Shi (2015) | + | + | + | PY | + | + | - | + | + | NA | + | + | NA | + | + | + | + | + |
| Sridharan (2021) | + | - | - | PY | + | + | - | PY | + | NA | - | - | NA | - | + | - | + | + |
| Sun (2016) | + | - | + | PY | - | + | - | + | PY | PY | - | + | + | - | - | + | + | - |
| Tang (2015) | + | - | + | PY | - | + | - | + | + | NA | - | + | NA | + | + | + | - | + |
| Tian (2021) | + | - | - | PY | - | + | - | - | NA | - | - | NA | + | + | + | + | + | + |
| Tse (2018) | + | - | + | PY | + | - | - | PY | + | NA | - | + | NA | - | - | + | + | + |
| Xu (2014) | - | - | + | - | - | + | - | + | + | NA | - | + | NA | - | - | + | + | + |
| Yang (2019) | + | - | + | PY | + | + | - | PY | + | NA | - | - | NA | + | - | + | + | + |
| Yu (2016) | + | - | + | PY | - | + | - | + | PY | PY | - | + | + | + | + | + | + | + |
| **Self-monitoring reviews** | | | | | | | | | | | | | | | | | | |
| Afzal (2019) | + | - | - | PY | + | + | - | + | + | PY | - | NA | NA | NA | - | - | NA | + |
| Buck (2021) | + | - | - | - | + | - | - | + | NA | + | - | NA | NA | NA | - | + | NA | - |
| Clarkesmith (2017) | + | + | - | + | + | + | + | + | + | NA | + | + | NA | + | + | + | + | + |
| Deitelzweig (2021) | + | + | + | PY | + | + | - | + | NA | + | + | NA | + | - | + | + | - | - |
| Dhippayom (2020) | + | + | + | PY | + | + | - | + | + | + | - | + | + | - | + | + | + | + |
| Dhippayom (2021) | + | + | - | PY | + | + | - | + | + | NA | - | + | NA | - | + | + | + | + |
| Entezari-Maleki (2016) | + | - | - | PY^[[4]](#footnote-4)^ | + | + | + | + | + | + | - | NA^[[5]](#footnote-5)^ | NA | NA | - | + | - | + |
| Heneghan (2016) | + | + | - | PY^[[6]](#footnote-6)^ | + | + | + | + | + | NA | + | NA | NA | NA | - | - | NA | + |
| Hou (2017) | + | - | + | PY | + | + | - | + | + | PY | - | + | + | + | + | + | + | - |
| Jang (2021) | + | - | - | PY | + | + | - | + | + | + | - | NA | NA | NA | + | + | NA | + |
| Manzoor (2017) | + | PY^[[7]](#footnote-7)^ | - | PY | + | + | - | + | + | + | - | NA | NA | NA | - | - | NA | + |
| Ng (2020) | + | + | - | PY | - | + | - | + | + | NA | + | + | NA | + | + | + | + | + |
| Ozaki (2020) | + | + | + | PY | + | + | - | + | NA | PY | -^[[8]](#footnote-8)^ | NA | + | + | + | + | + | - |
| Prentice (2020) | + | - | + | PY | + | + | - | + | NA | + | - | NA | + | - | - | - | - | - |
| Romoli (2021) | + | + | - | PY | - | - | + | PY | NA | PY | - | NA | - | - | - | + | - | + |
| Salmasi (2020) | + | - | - | PY | + | - | - | + | NA | + | + | NA | + | + | - | + | + | + |
| Sharma (2015) | + | PY | + | PY | + | + | + | + | + | NA | + | + | NA | -^[[9]](#footnote-9)^ | + | + | - | + |
| Shehab (2019) | + | - | + | - | - | + | + | PY | NA | - | - | NA | + | -^[[10]](#footnote-10)^ | + | - | + | + |
| Song (2021) | + | - | + | PY | - | - | - | PY | + | NA | - | - | NA | - | + | + | - | + |
| Torres (2021) | + | PY | - | PY | + | + | - | PY | + | PY | - | NA | NA | NA | + | - | NA | + |
| Tran (2021) | + | - | - | - | + | + | - | PY | - | - | - | + | + | + | + | + | + | + |
| Xia (2018) | + | - | - | - | - | + | - | PY | - | PY | - | - | - | - | + | + | + | + |
| Zhou (2016) | + | - | + | -^[[11]](#footnote-11)^ | + | + | - | + | + | NA | - | + | NA | + | + | + | - | - |

+ = yes, - = no, PY = partial yes, NA = not applicable, PICO = population, intervention, comparator, outcome, DE = data extraction, RoB = risk of bias, RCT = randomised controlled trial, NRSI = non-randomised studies of interventions

**Risk of bias summary**

For efficacy and safety, the reviewers did not provide references for the reasons for exclusion at full text and did not report an assessment of publication bias. The review was assessed as at a low risk of bias.

The most common flaws in the 17 genotyping reviews were in reporting the reasons for exclusion for each reference assessed at full text (none did this), reporting the sources of funding in the included studies (two reviews reported this), and reporting a protocol and any changes (five reviews reported this). Ten reviews did not use the risk of bias results in either the analysis or the discussion of results, or both. Nine reviews did not provide sufficient detail on their inclusion criteria, and eight did not report duplicate screening methods. Only three reviews reported a comprehensive search strategy, but only three did not report an adequate search strategy. Four or fewer reviews did not fully meet the remaining criteria. Shi (2015) met 14 and partly met one of their 16 relevant criteria, while Ng (2020) met 12 and partly met one of the 16 criteria. Jin (2014) only met three and partly met one of the 16 relevant criteria. Goulding (2015) met just under half of the relevant criteria. The remaining reviews met most of the criteria. Excluding Jin (2014), generally, the risk of bias in the reviews was moderate.

In the 23 self-monitoring or adherence reviews, the most common flaws were not fully reporting exclusions (six reviews did provide references by reason for exclusion) and funding sources for the included studies (six reviews did report these). Fifteen reviews did not use their risk of bias assessments in either the analysis or discussion of the results, or both. Fourteen reviews did not fully describe their inclusion criteria. Twelve reviews did not report having a protocol and any amendments. Seven or fewer reviews did not fully meet the remaining criteria. Clarkesmith (2017) met 15 of 16 relevant criteria, Sharma (2015) met 12 and partly met two of 16 criteria, and Dhippayom (2020) met 14 and partly met one of 18 relevant criteria (13 of 16 domains). Buck (2021), Prentice (2020), Romoli (2021), Song (2021), and Xia (2018) all met just under half of their relevant criteria. Afzal (2019), Manzoor (2017), Shehab (2019), Torres Roldan (2021), and Tran (2021) met half or just over half of the relevant criteria, and the remaining reviews met most of their relevant criteria. Generally, the risk of bias in the reviews was moderate to low.

1. There was no statement to say whether and to what extent deviations from the protocol were made. [↑](#footnote-ref-1)
2. The search was last updated two and a half years before publication. [↑](#footnote-ref-2)
3. It was unclear whether the risk of bias was integrated into the data synthesis; the terms relating to evidence (e.g., little evidence) were not clearly defined. [↑](#footnote-ref-3)
4. There was no search of the grey literature. [↑](#footnote-ref-4)
5. The means were pooled, but no meta-analysis weighted by sample size was conducted. [↑](#footnote-ref-5)
6. Searches were updated in July 2015, less than 24 months before publication. [↑](#footnote-ref-6)
7. The protocol was given, but the authors did not state whether there were any deviations from the protocol. [↑](#footnote-ref-7)
8. The authors extracted the information, but only reported a summary, not individual study information. [↑](#footnote-ref-8)
9. Risk of bias was considered for other analyses, but not for time in therapeutic range (TTR). [↑](#footnote-ref-9)
10. The impact was assessed, but no results were reported. [↑](#footnote-ref-10)
11. Did not justify the restriction of the searches to English-language journal articles. [↑](#footnote-ref-11)
