# Supplementary material for: Oral anticoagulants: a systematic overview of reviews on efficacy and safety, genotyping, self-monitoring, and stakeholder experiences
Source: Syst Rev. 2022 Oct 28;11:232. doi: 10.1186/s13643-022-02098-w (PMC9615370; doi:10.1186/s13643-022-02098-w)
Supplement: Supplementary file 8 — Additional file 8. Overlap in studies included in reviews for each section. [file 13643_2022_2098_MOESM8_ESM.docx]

# Overlap summary

The full references for the primary studies within the reviews are listed within each systematic review publication. The calculation for the corrected covered area (CCA) (Pieper et al., 2014) is (n-r)/(rc-r) x 100 (for percentage), where r is the number of rows (primary studies), c is the number of columns (reviews) and n is the total number of included studies (with double counting). Percentages of 0 to 5 are considered slight, 6 to 10 moderate, 11 to 15 high, and over 15 very high.

| **Review section** | **Corrected Covered Area (CCA)** | **Overlap assessment** | **Publication years of reviews** | **Number of reviews (unique studies)** | **Summary** |
| --- | --- | --- | --- | --- | --- |
| Efficacy and safety | 0% | None | 2017 | 1 (23 AF, 43 VTE PP, 9 VTE Tt, 10 VTE SP) | We included one systematic review with four sections. There was no overlap between the four sections, as they covered different medical conditions, treatments or prevention. |
| Genotyping | 4.5% | Slight | 2014 to 2021 | 17 (155) | Overlap was slight; seven of the 155 studies were included in nine to 11 reviews; one was in seven; one was in six; nine were in three to five; and 10 were in two reviews. One review included 77 studies; the other 16 reviews included 26 or fewer studies. |
| Self-monitoring – education or decision aids | 4.9% | Slight | 2017 to 2021 | 4 (27) | All reviews overlapped, but overlap was slight. One of the 27 studies was in three reviews, and two in two reviews. Three of the four reviews were published in 2021. |
| Self-monitoring – management or testing | 13.7% | High | 2015 to 2021 | 8 (45) | Overlap was high, with three of the 45 studies in four or five reviews; 11 in three reviews, and 11 in two reviews. |
| Self-monitoring – pharmacist management | 21.2% | Very high | 2016 to 2017 | 4 (33) | The four reviews were published within two years, with very high overlap. Two of the 33 studies were in all four reviews, four in three reviews, and seven in two reviews. |
| Self-monitoring – adherence measures | 7.0% | Moderate | 2019 to 2021 | 8 (131) | The eight reviews were published within three years, with moderate overlap; Afzal, 2019 had no overlap with the other reviews. Two of the 131 studies were in four reviews, four in three, and 29 in two reviews. |
| Stakeholder experiences | 1.4% | Slight | 2016 to 2021 | 15 (237) | There was slight overlap, with one of 237 studies in four reviews, three in three reviews, and 38 in two reviews. |

AF = atrial fibrillation, VTE = venous thromboembolism, PP = primary prevention, Tt = treatment, SP = secondary prevention

## Efficacy and safety review: no overlap as each section was on a different condition

|  | **Included primary studies relevant to this review** |
| --- | --- |
| Sterne et al. (review 1)  Prevention of stroke due to AF | The ACTIVE Writing Group 2006; Petersen et al. 1989; Gulløv et al. 1998; Liu et al. 2014; Yamaguchi 2010; Chung et al. 2011; Garcia et al. 2010; Weitz et al. 2010; Chen et al. 2012; Lopes et al. 2010; Granger et al. 2011; Hohnloser et al. 2012; Easton et al. 2012; Flaker et al. 2013; Al-Khatib et al. 2013; Bahit et al. 2013; Garcia et al. 2013; McMurray et al. 2013; Alexander et al. 2014; Hylek et al. 2014; Ogawa et al. 2011; Eikelboom et al. 2010; Hohnloser et al. 2011; Connolly et al. 2011; Diener et al. 2012; Mant et al. 2007; Hu et al. 2006; Ruff et al. 2010; Giugliano et al. 2013; Connolly et al. 2013; Hori et al. 2012; Hellemons et al. 1999; Ezekowitz et al. 2007; Connolly et al. 2009; Connolly et al. 2010; ROCKET AF Study Investigators 2010; Patel et al. 2011; Hankey et al. 2012; Mahaffey et al. 2013; Stroke Prevention in Atrial Fibrillation Investigators 1994; Rash et al. 2007 |
| Sterne et al. (review 2)  Prevention of VTE (primary) | Goldhaber et al. 2011; Lassen et al. 2009; Lassen et al. 2010b; Lassen et al. 2010a; Lassen et al. 2007; Heit et al. 1997; Eriksson et al. 2005; Turpie et al. 2009a; Kakkar et al. 2011; Cohen et al. 2010; Cohen et al. 2013; Eriksson et al. 2007a; Eriksson et al. 2006a; Turpie et al. 2005; Eriksson et al. 2006b; Agnelli et al. 2009; Eriksson et al. 2008; Kakkar et al. 2008; Lassen et al. 2008; Turpie et al. 2009b; Ginsberg et al. 2009; Eriksson et al. 2007c; Eriksson et al. 2007b; Eriksson et al. 2010; Eriksson et al. 2011; Fuji et al. 2010d; Fuji et al. 2009a; Fuji et al. 2010b; Fuji et al. 2009b; Fuji et al. 2014, Fujita et al. 2010; Fuji et al. 2010c; Haas et al. 2012; Levine et al. 2012; Iliopoulos et al. 2011; Fuji et al. 2010a; Raskob et al. 2010; Goel et al. 2009; Yokote et al. 2011; Kanan et al. 2008; Zhang et al. 2013; Leclerc et al. 1996; Francis et al. 1997; Colwell et al. 1999; Hull et al. 2000; Fitzgerald et al. 2001 |
| Sterne et al. (review 3)  Treatment of VTE | Hokusai et al. 2013; Agnelli et al. 2007; Büller et al. 2008; Schulman et al. 2009; Bauersachs et al. 2010**;** Büller et al. 2012; Raskob et al. 2013; Hokusai et al. 2013; Agnelli et al. 2013; Schulman et al 2014 |
| Sterne et al. (review 4)  Secondary prevention of VTE | Agnelli et al. 2012; Brighton et al. 2012; Buller 2009; The EINSTEIN Investigators 2010; Romualdi et al. 2011; Kearon et al. 1999; Ridker et al. 2003; Schulman et al. 2013; Becattini et al. 2012; Agnelli et al. 2001; Agnelli et al. 2003 |

AF = atrial fibrillation, VTE = venous thromboembolism

# Overlap of primary studies within reviews for genotyping

|  | **Review** | Sridharan (2021) | Tian (2021) | Asiimwe (2020) | Ng (2020)* | Yang (2019) | Kheiri (2018) | Tse (2018) | Chen (2016) | Sun (2016) | Yu (2016) | Dahal (2015) | Goulding (2015) | Shi (2015) | Tang (2015) | Franchini (2014) | Jin (2014) | Xu (2014) | **Overlap** |
| --- | --- | --- | --- | --- | --- | --- | --- | --- | --- | --- | --- | --- | --- | --- | --- | --- | --- | --- | --- |
|  | **Included study** |  |  |  |  |  |  |  |  |  |  |  |  |  |  |  |  |  |  |
| 1 | Anderson et al. (2007) | ✔ |  |  |  | ✔ | ✔ | ✔ |  |  |  | ✔ | ✔ | ✔ | ✔ | ✔ |  | ✔ | **10** |
| 2 | Aquilante et al. (2006) |  |  | ✔ |  |  |  |  |  |  |  |  |  |  |  |  | ✔ |  | **2** |
| 3 | Arwood et al. (2017) |  |  | ✔ |  |  |  |  |  |  |  |  |  |  |  |  |  |  |  |
| 4 | Bazan et al. (2013) |  |  |  |  |  |  |  |  |  |  |  |  |  |  |  | ✔ |  |  |
| 5 | Bejarano-Achache et al. (2012) |  |  |  |  |  |  |  | ✔ |  |  |  |  |  |  |  |  |  |  |
| 6 | Biffi et al. (2011) |  |  | ✔ |  |  |  |  |  |  |  |  |  |  |  |  |  |  |  |
| 7 | Borgman et al. (2012) | ✔ |  |  |  | ✔ | ✔ | ✔ |  |  |  | ✔ | ✔ | ✔ |  | ✔ |  | ✔ | **9** |
| 8 | Bress et al. (2012) |  |  | ✔ |  |  |  |  |  |  |  |  |  |  |  |  |  |  |  |
| 9 | Burmester et al. (2011) | ✔ |  |  |  | ✔ | ✔ | ✔ |  |  |  | ✔ | ✔ | ✔ | ✔ | ✔ |  | ✔ | **10** |
| 10 | Caraco et al. (2008) | ✔ |  |  |  |  | ✔ | ✔ |  |  |  | ✔ | ✔ | ✔ |  | ✔ |  |  | **7** |
| 11 | Cavallari et al. (2009) |  |  | ✔ |  |  |  |  |  |  |  |  |  |  |  |  |  |  |  |
| 12 | Cavallari et al. (2010) |  |  | ✔ |  |  |  |  |  |  | ✔ |  |  |  |  |  |  |  | **2** |
| 13 | Cavallari et al. (2011) |  |  | ✔ |  |  |  |  |  |  |  |  |  |  |  |  |  |  |  |
| 14 | Cavallari et al. (2012) |  |  | ✔ |  |  |  |  |  |  |  |  |  |  |  |  |  |  |  |
| 15 | Cavallari et al. (2013) |  |  | ✔ |  |  |  |  |  |  |  |  |  |  |  |  |  |  |  |
| 16 | Cerezo-Manchado et al. (2014) |  |  |  |  |  |  |  | ✔ |  |  |  |  |  |  |  |  |  |  |
| 17 | Cerezo-Manchado et al. (2016) |  |  |  |  |  | ✔ |  |  |  |  |  |  |  |  |  |  |  |  |
| 18 | Chung et al. (2015) |  | ✔ |  |  |  |  |  |  |  |  |  |  |  |  |  |  |  |  |
| 19 | Cosgun et al. (2011) |  |  | ✔ |  |  |  |  |  |  |  |  |  |  |  |  |  |  |  |
| 20 | Daneshjou et al. (2014) |  |  | ✔ |  |  |  |  |  |  |  |  |  |  |  |  |  |  |  |
| 21 | De et al. (2018) |  |  | ✔ |  |  |  |  |  |  |  |  |  |  |  |  |  |  |  |
| 22 | Drozda et al. (2015) |  |  | ✔ |  |  |  |  |  |  |  |  |  |  |  |  |  |  |  |
| 23 | Duan et al. (2016) | ✔ |  |  |  |  |  | ✔ |  |  |  |  |  |  |  |  |  |  | **2** |
| 24 | Fang et al. (2014) |  |  |  |  |  |  |  |  | ✔ |  |  |  |  |  |  |  |  |  |
| 25 | Ferder et al. (2010) |  |  | ✔ |  |  |  |  |  |  |  |  |  |  |  |  |  |  |  |
| 26 | Finkelman et al. (2015) |  |  | ✔ |  |  |  |  |  |  |  |  |  |  |  |  |  |  |  |
| 27 | Freeman et al. (2000) |  |  | ✔ |  |  |  |  |  |  |  |  |  |  |  |  |  |  |  |
| 28 | Fuchshuber-Moraes et al. (2009) |  |  | ✔ |  |  |  |  |  |  |  |  |  |  |  |  |  |  |  |
| 29 | Gage et al. (2004) |  |  | ✔ |  |  |  |  |  |  |  |  |  |  |  |  |  |  |  |
| 30 | Gage et al. (2008) |  |  | ✔ |  |  |  |  |  |  |  |  |  |  |  |  |  |  |  |
| 31 | Gage et al. (2017) | ✔ |  | ✔ |  | ✔ | ✔ | ✔ |  |  |  |  |  |  |  |  |  |  | **5** |
| 32 | Gan et al. (2011) |  |  |  |  |  |  |  |  |  |  |  |  |  |  |  | ✔ |  |  |
| 33 | Guo et al. (2020) | ✔ |  |  |  |  |  |  |  |  |  |  |  |  |  |  |  |  |  |
| 34 | Hernandez et al. (2014) |  |  | ✔ |  |  |  |  |  |  |  |  |  |  |  |  |  |  |  |
| 35 | Hernandez et al. (2015) |  |  | ✔ |  |  |  |  |  |  |  |  |  |  |  |  |  |  |  |
| 36 | Hernandez et al. (2017) |  |  | ✔ |  |  |  |  |  |  |  |  |  |  |  |  |  |  |  |
| 37 | Hillman et al. (2005) | ✔ |  |  |  | ✔ | ✔ | ✔ |  |  |  | ✔ | ✔ | ✔ | ✔ | ✔ |  | ✔ | **10** |
| 38 | Hirai et al. (2015) |  |  |  |  |  |  |  |  | ✔ |  |  |  |  |  |  |  |  |  |
| 39 | Huang et al. (2009) | ✔ |  |  |  | ✔ | ✔ | ✔ |  |  |  |  | ✔ |  |  |  |  |  | **5** |
| 40 | Iwuchukwu et al. (2016) |  |  | ✔ |  |  |  |  |  |  |  |  |  |  |  |  |  |  |  |
| 41 | Jiang et al. (2016) |  |  |  |  | ✔ |  |  |  |  |  |  |  |  |  |  |  |  |  |
| 42 | Jimenez-Varo et al. (2014) |  |  |  |  |  |  |  | ✔ |  |  |  |  |  |  |  |  |  |  |
| 43 | Jin et al. (2017) | ✔ |  |  |  | ✔ | ✔ | ✔ |  |  |  |  |  |  |  |  |  |  | **4** |
| 44 | Joffe et al. (2004) |  |  | ✔ |  |  |  |  |  |  |  |  |  |  |  |  |  |  |  |
| 45 | John et al. (2010) |  |  |  |  |  |  |  |  |  | ✔ |  |  |  |  |  |  |  |  |
| 46 | Jonas et al. (2013) | ✔ |  | ✔ |  | ✔ | ✔ | ✔ |  |  |  | ✔ |  | ✔ | ✔ | ✔ |  | ✔ | **10** |
| 47 | Kawai et al. (2014) |  |  | ✔ |  |  |  |  | ✔ |  |  |  |  |  |  |  |  |  | **2** |
| 48 | Kealey et al. (2007) |  |  | ✔ |  |  |  |  |  |  |  |  |  |  |  |  |  |  |  |
| 49 | Kim et al. (2013) |  |  | ✔ |  |  |  |  |  |  |  |  |  |  |  |  |  |  |  |
| 50 | Kimmel et al. (2007) |  |  |  |  |  |  |  |  |  | ✔ |  |  |  |  |  |  |  |  |
| 51 | Kimmel et al. (2008) |  |  | ✔ |  |  |  |  |  |  |  |  |  |  |  |  |  |  |  |
| 52 | Kimmel at al. (2013) | ✔ |  | ✔ |  | ✔ | ✔ | ✔ |  |  |  | ✔ | ✔ | ✔ | ✔ | ✔ |  | ✔ | **11** |
| 53 | King et al. (2010) |  |  | ✔ |  |  |  |  |  |  |  |  |  |  |  |  |  |  |  |
| 54 | Kohnke et al. (2005) |  |  |  |  |  |  |  |  |  | ✔ |  |  |  |  |  |  |  |  |
| 55 | Krishna et al. (2014) |  |  |  |  |  |  |  |  | ✔ |  |  |  |  |  |  |  |  |  |
| 56 | Kwon et al. (2011) |  |  |  |  |  |  |  |  |  |  |  |  |  |  |  | ✔ |  |  |
| 57 | Kudzi et al. (2016) |  |  | ✔ |  |  |  |  |  |  |  |  |  |  |  |  |  |  |  |
| 58 | Lal et al. (2007) |  |  |  |  |  |  |  |  |  | ✔ |  |  |  |  |  |  |  |  |
| 59 | Langley et al. (2009) |  |  | ✔ |  |  |  |  |  |  |  |  |  |  |  |  |  |  |  |
| 60 | Lee et al. (2009) |  |  |  |  |  |  |  |  | ✔ |  |  |  |  |  |  | ✔ |  | **2** |
| 61 | Lee et al. (2020) | ✔ |  |  |  |  |  |  |  |  |  |  |  |  |  |  |  |  |  |
| 62 | Lenzini et al. (2008) |  |  | ✔ |  |  |  |  |  |  |  |  |  |  |  |  |  |  |  |
| 63 | Li et al. (2009) |  |  | ✔ |  |  |  |  |  |  |  |  |  |  |  |  |  |  |  |
| 64 | Li et al. (2013) | ✔ |  |  |  | ✔ | ✔ | ✔ |  |  |  |  |  |  |  |  |  |  | **4** |
| 65 | Li et al. (2017) |  |  |  |  |  | ✔ |  |  |  |  |  |  |  |  |  |  |  |  |
| 66 | Li et al. (2018) |  | ✔ |  |  |  |  |  |  |  |  |  |  |  |  |  |  |  |  |
| 67 | Li et al. (2020) |  | ✔ |  |  |  |  |  |  |  |  |  |  |  |  |  |  |  |  |
| 68 | Liang et al. (2012) |  |  |  |  |  |  |  |  | ✔ |  |  |  |  |  |  | ✔ |  | **2** |
| 69 | Limdi et al. (2006) |  |  | ✔ |  |  |  |  |  |  |  |  |  |  |  |  |  |  |  |
| 70 | Limdi et al. (2007) |  |  | ✔ |  |  |  |  |  |  |  |  |  |  |  |  |  |  |  |
| 71 | Limdi et al. (2008a) |  |  | ✔ |  |  |  |  |  |  |  |  |  |  |  |  |  |  |  |
| 72 | Limdi et al. (2008b) |  |  | ✔ |  |  |  |  |  |  |  |  |  |  |  |  |  |  |  |
| 73 | Limdi et al. (2008c) |  |  | ✔ |  |  |  |  |  |  |  |  |  |  |  |  |  |  |  |
| 74 | Limdi et al. (2009) |  |  | ✔ |  |  |  |  |  |  |  |  |  |  |  |  |  |  |  |
| 75 | Limdi et al. (2015) |  |  | ✔ |  |  |  |  |  |  |  |  |  |  |  |  |  |  |  |
| 76 | Limdi et al. (2017) |  |  | ✔ |  |  |  |  |  |  |  |  |  |  |  |  |  |  |  |
| 77 | Liu et al. (2017) |  |  | ✔ |  |  |  |  |  |  |  |  |  |  |  |  |  |  |  |
| 78 | Lou et al. (2014) |  |  |  |  |  |  |  |  | ✔ |  |  |  |  |  |  |  |  |  |
| 79 | Lubitz et al. (2010) |  |  | ✔ |  |  |  |  |  |  |  |  |  |  |  |  |  |  |  |
| 80 | Ma et al. (2012) |  |  |  |  |  |  |  | ✔ |  |  |  |  |  |  |  |  |  |  |
| 81 | Makar-Ausperger et al. (2018) | ✔ |  |  |  |  |  |  |  |  |  |  |  |  |  |  |  |  |  |
| 82 | Mallal et al. (2008) |  |  |  |  |  |  |  |  |  |  |  | ✔ |  |  |  |  |  |  |
| 83 | McMillin et al. (2010) | ✔ |  |  |  |  |  |  |  |  |  |  |  |  |  |  |  |  |  |
| 84 | Meynard et al. (2002) |  |  |  |  |  |  |  |  |  |  |  | ✔ |  |  |  |  |  |  |
| 85 | Miao et al. (2007) |  |  |  |  |  |  |  |  |  |  |  |  |  |  |  | ✔ |  |  |
| 86 | Michaud et al. (2008) |  |  |  |  |  |  |  |  |  |  |  |  |  |  |  | ✔ |  |  |
| 87 | Millican et al. (2007) |  |  | ✔ |  |  |  |  |  |  |  |  |  |  |  |  |  |  |  |
| 88 | Mitchell et al. (2011) |  |  | ✔ |  |  |  |  |  |  |  |  |  |  |  |  |  |  |  |
| 89 | Momary et al. (2007) |  | ✔ | ✔ |  |  |  |  |  |  |  |  |  |  |  |  | ✔ |  | **3** |
| 90 | Namazi et al. (2010) |  |  |  |  |  |  |  |  |  |  |  |  |  |  |  | ✔ |  |  |
| 91 | Ndadza et al. (2019) |  |  | ✔ |  |  |  |  |  |  |  |  |  |  |  |  |  |  |  |
| 92 | Newman et al. (2011) |  |  |  |  |  |  |  |  |  |  |  | ✔ |  |  |  |  |  |  |
| 93 | Nguyen et al. (2013) |  |  | ✔ |  |  |  |  |  |  |  |  |  |  |  |  |  |  |  |
| 94 | Obayashi et al. (2006) |  |  |  |  |  |  |  |  |  |  |  |  |  |  |  | ✔ |  |  |
| 95 | Ohno et al. (2009) |  |  |  |  |  |  |  |  |  |  |  |  |  |  |  | ✔ |  |  |
| 96 | Oliveria Almeida et al. (2014) |  |  |  |  |  |  |  |  |  | ✔ |  |  |  |  |  |  |  |  |
| 97 | Oner Ozgon et al. (2008) |  |  |  |  |  |  |  |  |  |  |  |  |  |  |  | ✔ |  |  |
| 98 | Orsi et al. (2010) |  |  | ✔ |  |  |  |  |  |  |  |  |  |  |  |  |  |  |  |
| 99 | Ozer et al. (2010) |  |  |  |  |  |  |  |  |  |  |  |  |  |  |  | ✔ |  |  |
| 100 | Özer et al. (2013) |  |  |  |  |  |  |  |  | ✔ |  |  |  |  |  |  | ✔ |  | **2** |
| 101 | Panchenko et al. (2020) | ✔ |  |  |  |  |  |  |  |  |  |  |  |  |  |  |  |  |  |
| 102 | Parra et al. (2015) |  |  | ✔ |  |  |  |  |  |  |  |  |  |  |  |  |  |  |  |
| 103 | Pautas et al. (2010) |  |  |  |  |  |  |  |  |  |  |  |  |  |  |  | ✔ |  |  |
| 104 | Pengo et al. (2015) | ✔ |  |  |  | ✔ | ✔ | ✔ |  |  |  |  |  |  |  |  |  |  | **4** |
| 105 | Perera et al. (2011) |  |  | ✔ |  |  |  |  |  |  |  |  |  |  |  |  |  |  |  |
| 106 | Perera et al. (2013) |  |  | ✔ |  |  |  |  |  |  |  |  |  |  |  |  |  |  |  |
| 107 | Perini et al. (2008) |  |  | ✔ |  |  |  |  |  |  |  |  |  |  |  |  |  |  |  |
| 108 | Perini et al. (2010) |  |  | ✔ |  |  |  |  |  |  |  |  |  |  |  |  |  |  |  |
| 109 | Pirmohamed et al. (2013) | ✔ |  |  | ✔ | ✔ | ✔ | ✔ |  |  |  | ✔ | ✔ | ✔ | ✔ | ✔ |  | ✔ | **11** |
| 110 | Radhakrishnan et al. (2012) | ✔ |  |  |  |  | ✔ | ✔ |  |  |  |  |  |  |  |  |  |  | **3** |
| 111 | Ramirez et al. (2012) |  |  | ✔ |  |  |  |  |  |  |  |  |  |  |  |  |  |  |  |
| 112 | Ramos et al. (2012) |  |  | ✔ |  |  |  |  |  |  |  |  |  |  |  |  |  |  |  |
| 113 | Roberts et al. (2012) |  |  |  |  |  |  |  |  |  |  |  | ✔ |  |  |  |  |  |  |
| 114 | Roth et al (2014) |  |  |  |  |  |  |  | ✔ |  |  |  |  |  |  |  |  |  |  |
| 115 | Sagreiya et al. (2010) |  |  | ✔ |  |  |  |  |  |  |  |  |  |  |  |  |  |  |  |
| 116 | Santos et al. (2013) |  |  |  |  |  |  |  |  |  |  |  |  |  |  |  | ✔ |  |  |
| 117 | Santos et al. (2015) |  |  | ✔ |  |  |  |  |  |  |  |  |  |  |  |  |  |  |  |
| 118 | Schelleman et al. (2007) |  |  | ✔ |  |  |  |  |  |  |  |  |  |  |  |  |  |  |  |
| 119 | Schelleman et al. (2008) |  |  | ✔ |  |  |  |  |  |  |  |  |  |  |  |  |  |  |  |
| 120 | Schelleman et al. (2010) |  |  | ✔ |  |  |  |  |  |  |  |  |  |  |  |  |  |  |  |
| 121 | Schwarz et al. (2008) |  |  | ✔ |  |  |  |  |  |  |  |  |  |  |  |  |  |  |  |
| 122 | Sconce et al. (2005) |  |  |  |  |  |  |  |  |  |  |  |  |  |  |  | ✔ |  |  |
| 123 | Sconce et al. (2006) |  |  |  |  |  |  |  |  |  | ✔ |  |  |  |  |  |  |  |  |
| 124 | Shahin et al. (2011) |  |  |  |  |  |  |  |  |  | ✔ |  |  |  |  |  | ✔ |  | **2** |
| 125 | Shendre et al. (2014) |  |  | ✔ |  |  |  |  |  |  |  |  |  |  |  |  |  |  |  |
| 126 | Shendre et al. (2016) |  |  | ✔ |  |  |  |  |  |  |  |  |  |  |  |  |  |  |  |
| 127 | Shendre et al. (2018) |  |  | ✔ |  |  |  |  |  |  |  |  |  |  |  |  |  |  |  |
| 128 | Sheng Wen et al. (2011) |  |  |  |  |  |  |  |  |  | ✔ |  |  |  |  |  |  |  |  |
| 129 | Shrif et al. (2011) |  |  | ✔ |  |  |  |  |  |  |  |  |  |  |  |  | ✔ |  | **2** |
| 130 | Singh et al. (2011) |  |  |  |  |  |  |  |  | ✔ |  |  |  |  |  |  |  |  |  |
| 131 | Smires et al. (2012) |  |  |  |  |  |  |  |  |  |  |  |  |  |  |  | ✔ |  |  |
| 132 | Stack et al. (2016) |  |  | ✔ |  |  |  |  |  |  |  |  |  |  |  |  |  |  |  |
| 133 | Supe et al. (2015) | ✔ |  |  |  |  |  | ✔ |  |  |  |  |  |  |  |  |  |  | **2** |
| 134 | Syn et al. (2018) | ✔ |  |  |  |  |  |  |  |  |  |  |  |  |  |  |  |  |  |
| 135 | Tabrizi et al. (2002) |  |  | ✔ |  |  |  |  |  |  |  |  |  |  |  |  |  |  |  |
| 136 | Teh et al. (2012) |  |  |  |  |  |  |  |  |  |  |  |  |  |  |  | ✔ |  |  |
| 137 | Thervet et al. (2010) |  |  |  |  |  |  |  |  |  |  |  | ✔ |  |  |  |  |  |  |
| 138 | Vear et al. (2014) |  |  | ✔ |  |  |  |  |  |  |  |  |  |  |  |  |  |  |  |
| 139 | Verhoef et al. (2013) | ✔ |  |  |  |  | ✔ |  |  |  |  |  | ✔ |  | ✔ |  |  |  | **4** |
| 140 | Voora et al. (2005) |  |  | ✔ |  |  |  |  |  |  |  |  |  |  |  |  |  |  |  |
| 141 | Voora et al. (2010) |  |  | ✔ |  |  |  |  |  |  |  |  |  |  |  |  |  |  |  |
| 142 | Wang et al. (2008) |  |  | ✔ |  |  |  |  |  |  |  |  |  |  |  |  |  |  |  |
| 143 | Wang et al. (2011) |  |  |  |  |  |  |  |  | ✔ |  |  |  |  |  |  |  |  |  |
| 144 | Wang et al. (2012) | ✔ |  |  |  | ✔ | ✔ | ✔ |  |  |  | ✔ | ✔ |  |  |  |  |  | **6** |
| 145 | Wen et al. (2017) | ✔ |  |  |  | ✔ | ✔ | ✔ |  |  |  |  |  |  |  |  |  |  | **4** |
| 146 | Wiley et al. (2017) |  |  | ✔ |  |  |  |  |  |  |  |  |  |  |  |  |  |  |  |
| 147 | Wu et al. (2008) |  |  | ✔ |  |  |  |  |  |  |  |  |  |  |  |  |  |  |  |
| 148 | Xu et al. (2018) | ✔ |  |  |  |  |  |  |  |  |  |  |  |  |  |  |  |  |  |
| 149 | Yoshizawa et al. (2009) |  |  |  |  |  |  |  |  |  |  |  |  |  |  |  | ✔ |  |  |
| 150 | Yuan et al. (2005) |  |  |  |  |  |  |  |  |  |  |  |  |  |  |  | ✔ |  |  |
| 151 | Zhang et al. (2009) |  |  |  |  |  |  |  | ✔ |  |  |  |  |  |  |  |  |  |  |
| 152 | Zhang et al. (2013) |  |  |  |  |  |  |  |  | ✔ |  |  |  |  |  |  |  |  |  |
| 153 | Zhe et al. (2016) |  |  |  |  |  | ✔ |  |  |  |  |  |  |  |  |  |  |  |  |
| 154 | Zhu et al. (2007) |  |  |  |  |  |  |  |  |  |  |  |  |  |  |  | ✔ |  |  |
| 155 | Zhu et al. (2012) |  |  |  |  |  |  |  |  | ✔ |  |  |  |  |  |  |  |  |  |
|  | **Total (n=266)** | 26 | 4 | 77 | 1 | 15 | 20 | 18 | 7 | 11 | 9 | 9 | 15 | 7 | 7 | 8 | 25 | 7 |  |

* Ng only included one study on genotype-guided dosing (see their results section page 3 paragraph 3); they included 36 studies on various comparators

a,b,c = Asiimwe (2020) included three studies by Limdi et al. 2008, all referenced in the review paper.

CCA = (266 - 155)/(155x17 - 155) = 4.5% = slight

## Overlap of primary studies within reviews for self-monitoring

### Education and decision aids

| **Review** | | Jang (2021) | Song (2021) | Torres Roldan (2021)* | Clarkesmith (2017) | **Overlap** |
| --- | --- | --- | --- | --- | --- | --- |
|  | **Included Studies** |  |  |  |  |  |
| 1 | Arts et al. (2017) |  | ✔ |  |  |  |
| 2 | Bajorek et al. (2016) |  | ✔ |  |  |  |
| 3 | Clarkesmith et al. (2013) |  |  |  | ✔ |  |
| 4 | Eckman et al. (2016) |  | ✔ |  |  |  |
| 5 | Eckman et al. (2018) |  |  | ✔ |  |  |
| 6 | Fraenkel et al. (2012) |  | ✔ |  |  |  |
| 7 | Guhl et al. (2020) | ✔ |  |  |  |  |
| 8 | Guo et al. (2017) | ✔ | ✔ | ✔ |  | **3** |
| 9 | Guo et al. (2020a) | ✔ |  |  |  |  |
| 10 | Guo et al. (2020b) | ✔ |  |  |  |  |
| 11 | Hong et al. (2013) |  |  | ✔ |  |  |
| 12 | Karlsson et al. (2018) |  | ✔ |  |  |  |
| 13 | Kunneman et al. (2020) |  |  | ✔ |  |  |
| 14 | Labovitz et al. (2017) | ✔ |  |  |  |  |
| 15 | Lee et al. (2016) | ✔ |  |  |  |  |
| 16 | Lin et al. (2014) | ✔ |  |  |  |  |
| 17 | Loewen et al. (2019) |  |  | ✔ |  |  |
| 18 | Man-Son-Hing et al. (1999) |  | ✔ |  |  |  |
| 19 | McAlister et al. (2005) |  | ✔ |  | ✔ | **2** |
| 20 | Prochaska et al. (2015) | ✔ |  |  |  |  |
| 21 | Prochaska et al. (2017) | ✔ |  |  |  |  |
| 22 | Shilbayeh et al. (2019) | ✔ |  |  |  |  |
| 23 | Stephan et al. (2018) | ✔ |  | ✔ |  | **2** |
| 24 | Talboom-Kamp et al. (2017) | ✔ |  |  |  |  |
| 25 | Thomson et al. (2007) |  | ✔ |  |  |  |
| 26 | Van Doorn et al. (2018) |  | ✔ |  |  |  |
| 27 | Vormfelde et al. (2014) |  |  |  | ✔ |  |
|  | **Total (n=31)** | 12 | 10 | 6 | 3 |  |

* evaluation studies only, not included tools

a and b = Jang (2021) included two studies by Guo et al (2020) both referenced in the review paper

CCA = (n-r)/(rc-r) = (31 - 27)/(27x4 - 27) = 4.9% = slight

### Self-testing and self-management

| **Review** | | Dhippayom (2021) | Tran (2021) | Dhippayom (2020) | Ng (2020)* | Xia (2018) | Clarkesmith (2017) | Heneghan (2016)** | Sharma (2015)** | **Overlap** |
| --- | --- | --- | --- | --- | --- | --- | --- | --- | --- | --- |
|  | **Included Studies** |  |  |  |  |  |  |  |  |  |
| 1 | Azarnoush et al. (2011) |  |  |  |  |  |  | ✔ | ✔ | **2** |
| 2 | Beyth et al. (2000) |  |  |  |  |  |  | ✔ |  |  |
| 3 | Blissit et al. (2015) |  | ✔ | ✔ |  | ✔ |  |  |  | **3** |
| 4 | Brasen et al. (2019) | ✔ |  |  |  |  |  |  |  |  |
| 5 | Cao et al. (2018) |  | ✔ | ✔ |  |  |  |  |  | **2** |
| 6 | Christensen et al. (2006) |  |  |  |  |  |  | ✔ |  |  |
| 7 | Christensen et al. (2007) |  |  |  |  |  | ✔ |  |  |  |
| 8 | Christensen et al. (2011) | ✔ |  |  |  |  |  | ✔ | ✔ | **3** |
| 9 | Cromheecke et al. (2000) |  |  |  |  |  |  |  | ✔ |  |
| 10 | Cryder et al. (2017) |  | ✔ | ✔ |  | ✔ |  |  |  | **3** |
| 11 | Dignan et al. (2013) | ✔ |  |  |  |  |  |  |  |  |
| 12 | Eitz et al. (2008) | ✔ |  |  |  |  |  |  |  |  |
| 13 | Fitzmaurice et al. (2002) | ✔ |  |  |  |  |  | ✔ | ✔ | **3** |
| 14 | Fitzmaurice et al. (2005) | ✔ |  |  |  |  |  | ✔ | ✔ | **3** |
| 15 | Gadisseur et al. (2003) |  |  |  |  |  | ✔ |  | ✔ | **2** |
| 16 | Gardiner et al. (2006) | ✔ |  |  |  |  |  | ✔ |  | **2** |
| 17 | Grunau et al. (2011) |  |  |  |  |  |  | ✔ |  |  |
| 18 | Hall et al. (2011) |  | ✔ | ✔ |  |  |  |  |  | **2** |
| 19 | Harper and Pollock (2011) |  |  | ✔ |  |  |  |  |  |  |
| 20 | Hawes et al. (2018) |  | ✔ |  |  |  |  |  |  |  |
| 21 | Kaatz et al. (unpublished) |  |  |  |  |  |  | ✔ |  |  |
| 22 | Khan et al. (2004) | ✔ |  |  | ✔ |  |  | ✔ | ✔ | **4** |
| 23 | Lee et al. (2017) |  |  |  |  | ✔ |  |  |  |  |
| 24 | Lee et al. (2018) |  | ✔ | ✔ |  |  |  |  |  | **2** |
| 25 | Lin et al. (2014) |  |  |  |  | ✔ |  |  |  |  |
| 26 | Matchar et al. (2010) | ✔ |  | ✔ | ✔ |  |  | ✔ | ✔ | **5** |
| 27 | Menendez-Jandula et al. (2005) |  |  |  | ✔ |  |  | ✔ | ✔ | **3** |
| 28 | Prochaska et al. (2015) |  |  |  |  | ✔ |  |  |  |  |
| 29 | Rasmussen et al. (2012) | ✔ |  |  |  |  |  | ✔ |  | **2** |
| 30 | Ryan et al. (2009) | ✔ |  | ✔ |  |  |  |  |  | **2** |
| 31 | Salvador et al. (2008) |  |  |  |  | ✔ |  |  |  |  |
| 32 | Sawicki (1999) |  |  |  |  |  |  |  | ✔ |  |
| 33 | Sidhu et al. (2001) | ✔ |  |  |  |  |  | ✔ |  | **2** |
| 34 | Siebenhofer et al. (2007) |  |  |  |  |  |  | ✔ |  |  |
| 35 | Singh et al. (2015) |  | ✔ |  |  |  |  |  |  |  |
| 36 | Staresinic et al. (2006) |  | ✔ | ✔ |  | ✔ |  |  |  | **3** |
| 37 | Stoudenmire et al. (2014) |  | ✔ |  |  | ✔ |  |  |  | **2** |
| 38 | Stoudenmire et al. (2016) |  |  | ✔ |  |  |  |  |  |  |
| 39 | Sunderji et al. (2004) | ✔ |  |  |  |  |  | ✔ | ✔ | **3** |
| 40 | Thompson et al. (2013) | ✔ |  |  |  |  |  | ✔ |  | **2** |
| 41 | Verret et al. (2012) | ✔ |  |  | ✔ |  |  | ✔ | ✔ | **4** |
| 42 | Voller et al. (2005) |  |  |  | ✔ |  | ✔ |  | ✔ | **3** |
| 43 | Witt et al. (2005) |  | ✔ | ✔ |  | ✔ |  |  |  | **3** |
| 44 | Wittkowsky et al. (2006) |  | ✔ | ✔ |  | ✔ |  |  |  | **3** |
| 45 | Yildirim et al. (2020) | ✔ |  |  |  |  |  |  |  |  |
|  | **Total (n=88)** | 16 | 11 | 12 | 5 | 10 | 3 | 18 | 13 |  |

* For Ng (2020) this only includes the five studies that reported self-testing or self-management (see their results section page 3 paragraph 3)

** Trials included for TTR or values in range only

CCA = (n-r)/(rc-r) = (88 - 45)/(45x8 - 45) = 13.7% = high

### Pharmacist-managed warfarin therapy

|  | **Review** | Hou (2017) | Manzoor (2017) | Entezari-Maleki (2016) | Zhou (2016) | Overlap |
| --- | --- | --- | --- | --- | --- | --- |
|  | **Included Studies** |  |  |  |  |  |
| 1 | Ariee et al. (2009) | ✔ |  |  |  |  |
| 2 | Bungard et al. (2009) |  | ✔ | ✔ |  | 2 |
| 3 | Bungard et al. (2012) | ✔ | ✔ | ✔ | ✔ | 4 |
| 4 | Chamberlain et al. (2001) |  | ✔ |  |  |  |
| 5 | Chan et al. (2006) | ✔ | ✔ |  |  | 2 |
| 6 | Chiquette et al. (1998) | ✔ | ✔ |  |  | 2 |
| 7 | Cohen et al. (1985) |  | ✔ |  |  |  |
| 8 | Duran-Parrondo et al. (2011) |  | ✔ |  |  |  |
| 9 | Elewa et al. (2016) | ✔ | ✔ |  |  | 2 |
| 10 | Garabedian-Ruffalo et al. (1985)^[[1]](#footnote-1)^ |  | ✔ |  |  |  |
| 11 | Garwood et al. (2008) |  | ✔ |  |  |  |
| 12 | Gray et al. (1985) |  | ✔ |  |  |  |
| 13 | Gupta et al. (2015) |  | ✔ |  |  |  |
| 14 | Hall et al. (2011) | ✔ | ✔ | ✔ |  | 3 |
| 15 | Harrison et al. (2015) |  | ✔ |  |  |  |
| 16 | Hasan et al. (2011) |  | ✔ |  |  |  |
| 17 | Holden and Holden (2000) |  | ✔ |  |  |  |
| 18 | Jackson et al. (2004) | ✔ |  |  |  |  |
| 19 | Lakshim et al. (2013) | ✔ |  |  |  |  |
| 20 | Lalonde et al. (2008) | ✔ |  | ✔ | ✔ | 3 |
| 21 | Martin et al. (2012) | ✔ |  |  |  |  |
| 22 | Motycka et al. (2012) |  | ✔ |  |  |  |
| 23 | Patel-Naik et al. (2010) |  | ✔ |  |  |  |
| 24 | Poon et al. (2007) |  | ✔ |  |  |  |
| 25 | Rudd and Dier (2010) |  | ✔ | ✔ |  | 2 |
| 26 | Saokaew et al. (2012) |  | ✔ |  |  |  |
| 27 | Sargent et al. (2016) | ✔ |  |  |  |  |
| 28 | Schillg et al. (2011) | ✔ |  |  |  |  |
| 29 | Thanimalai et al. (2013) | ✔ | ✔ |  |  | 2 |
| 30 | Verret et al. (2012) | ✔ |  |  | ✔ | 2 |
| 31 | Wilson et al. (2003) | ✔ | ✔ | ✔ | ✔ | 4 |
| 32 | Witt et al. (2005) | ✔ | ✔ | ✔ |  | 3 |
| 33 | Young et al. (2011) | ✔ | ✔ | ✔ |  | 3 |
|  | **Total (n=54)** | 17 | 25 | 8 | 4 |  |

CCA = (54 - 33)/(33x4 - 33) = 21.2% = very high

### Adherence/persistence/discontinuation/switching studies

|  | **Review** | Buck (2021) | Deitelzweig (2021) | Romoli (2021) | Ozaki (2020) | Prentice (2020) | Salmasi (2020) | Afzal (2019)* | Shebab (2019) | Overlap |
| --- | --- | --- | --- | --- | --- | --- | --- | --- | --- | --- |
|  | **Included Studies** |  |  |  |  |  |  |  |  |  |
| 1 | Alberts et al. (2016) |  |  |  | ✔ |  | ✔ |  |  | 2 |
| 2 | Al-Khalili et al. (2016) |  |  |  | ✔ |  |  |  |  |  |
| 3 | Annavarapu et al. (2018) |  | ✔ |  | ✔ |  |  |  |  | 2 |
| 4 | Baker et al. (2018) |  | ✔ |  |  |  |  |  |  |  |
| 5 | Baker et al. (2019a) |  |  | ✔ |  |  |  |  |  |  |
| 6 | Baker et al. (2019b) |  |  | ✔ |  |  |  |  |  |  |
| 7 | Bancroft et al. (2016) |  | ✔ |  | ✔ |  |  |  |  | 2 |
| 8 | Benzimura et al. (2018) |  |  |  |  |  |  | ✔ |  |  |
| 9 | Bertozzo et al. (2016) | ✔ |  |  |  |  |  |  |  |  |
| 10 | Beshir et al. (2016) |  |  |  | ✔ |  |  |  |  |  |
| 11 | Beyer-Westendorf et al. (2015) |  |  |  |  |  |  |  | ✔ |  |
| 12 | Beyer-Westendorf et al. (2016) |  |  |  | ✔ |  | ✔ |  |  | 2 |
| 13 | Borg et al. (2016) | ✔ |  |  |  |  |  |  |  |  |
| 14 | Borne et al 2017 |  |  |  | ✔ | ✔ | ✔ |  |  | 3 |
| 15 | Brown et al. (2016) |  |  |  |  |  | ✔ |  |  |  |
| 16 | Brown et al. (2017) |  |  |  | ✔ |  |  |  |  |  |
| 17 | Carruthers et al. (2014) |  |  |  |  |  |  | ✔ |  |  |
| 18 | Casciano et al. (2013) |  |  |  |  |  | ✔ |  |  |  |
| 19 | Castellucci et al. (2015) |  |  |  |  |  |  | ✔ |  |  |
| 20 | Cataldo et al. (2018) |  | ✔ |  | ✔ |  |  |  |  | 2 |
| 21 | Coleman et al. (2016a) |  |  |  | ✔ | ✔ | ✔ |  |  | 3 |
| 22 | Coleman et al. (2016b) |  | ✔ |  | ✔ |  |  |  |  | 2 |
| 23 | Coleman et al. (2017) |  |  |  |  |  | ✔ |  |  |  |
| 24 | Collings et al. (2017) |  | ✔ |  | ✔ |  |  |  |  | 2 |
| 25 | Collings et al. (2018) |  | ✔ |  | ✔ |  |  |  |  | 2 |
| 26 | Crivera et al. (2015) |  |  |  |  |  | ✔ |  |  |  |
| 27 | Cutler et al. (2014) |  |  |  | ✔ |  |  |  |  |  |
| 28 | Deshpande et al. (2018a) |  |  |  | ✔ |  | ✔ |  |  | 2 |
| 29 | Deshpande et al. (2018b) |  |  |  | ✔ |  | ✔ |  |  | 2 |
| 30 | Douros et al. (2017) |  |  |  | ✔ |  |  |  |  |  |
| 31 | Eapen et al. (2014) |  |  |  |  |  | ✔ |  |  |  |
| 32 | Forslund et al. (2016) |  |  |  | ✔ |  | ✔ |  |  | 2 |
| 33 | Graham et al. (2018) |  | ✔ |  |  |  |  |  |  |  |
| 34 | Gomez-Lumbreras et al. (2018) |  |  |  | ✔ |  | ✔ |  |  | 2 |
| 35 | Gopalakrishnan et al. (2019) |  |  | ✔ |  |  |  |  |  |  |
| 36 | Gorst-Rasmussen et al. (2015) |  |  |  | ✔ |  | ✔ |  | ✔ | 3 |
| 37 | Gumbinger et al. (2015) | ✔ |  |  |  |  |  |  |  |  |
| 38 | Haastrup et al. (2018) |  |  |  | ✔ |  |  |  |  |  |
| 39 | Hanemaaijer et al. (2015) |  |  |  |  |  |  |  | ✔ |  |
| 40 | Harper et al. (2018) |  |  |  | ✔ |  | ✔ |  |  | 2 |
| 41 | Hellfritzsch et al. (2017) |  |  |  | ✔ |  |  |  |  |  |
| 42 | Hernandez et al. (2017) |  |  |  | ✔ |  |  |  |  |  |
| 43 | Ho et al. (2014) | ✔ |  |  | ✔ |  |  |  |  | 2 |
| 44 | Jackevicius et al. (2017) |  |  |  | ✔ |  |  |  |  |  |
| 45 | Jackson et al. (2018) | ✔ |  |  |  |  |  |  |  |  |
| 46 | Jacobs et al. (2018) |  |  |  | ✔ |  | ✔ |  |  | 2 |
| 47 | Johnson et al. (2016) |  | ✔ |  | ✔ |  |  |  |  | 2 |
| 48 | Kachroo et al. (2016) |  |  |  | ✔ |  |  |  |  |  |
| 49 | Keita et al. (2017) |  |  |  |  |  |  | ✔ |  |  |
| 50 | Laliberte et al. (2014) |  | ✔ |  | ✔ |  |  |  |  | 2 |
| 51 | Lamberts et al. (2017) |  | ✔ |  | ✔ |  |  |  |  | 2 |
| 52 | Larochelle et al. (2018) |  |  |  |  |  |  | ✔ |  |  |
| 53 | Lau et al. (2015) |  | ✔ |  |  |  |  |  |  |  |
| 54 | Lip et al. (2018) |  | ✔ |  | ✔ |  |  |  |  | 2 |
| 55 | Luger et al. (2015) |  |  |  |  |  |  |  | ✔ |  |
| 56 | Manzoor et al. (2017a) |  |  | ✔ |  |  |  |  |  |  |
| 57 | Manzoor et al. (2017b) |  |  |  | ✔ |  | ✔ |  |  | 2 |
| 58 | Marquez-Contrera et al. (2016) |  |  |  |  |  | ✔ |  |  |  |
| 59 | Martinez et al. (2016) |  |  |  | ✔ |  |  |  |  |  |
| 60 | Maura et al. (2017) |  |  |  | ✔ |  | ✔ |  |  | 2 |
| 61 | Maura et al. (2018) |  | ✔ |  | ✔ |  |  |  |  | 2 |
| 62 | McAlister et al. (2018) |  |  |  |  |  | ✔ |  |  |  |
| 63 | McCormick et al. (2001) |  |  |  |  |  | ✔ |  |  |  |
| 64 | McHorney et al. (2015) |  |  |  |  | ✔ |  |  |  |  |
| 65 | McHorney et al. (2016) |  |  |  | ✔ |  |  |  |  |  |
| 66 | McHorney et al. (2017) |  | ✔ |  | ✔ | ✔ | ✔ |  |  | 4 |
| 67 | McHorney et al. (2018) |  |  |  |  |  | ✔ |  |  |  |
| 68 | Mueller et al. (2017) |  |  |  | ✔ |  | ✔ |  |  | 2 |
| 69 | Naganuma et al. (2017) | ✔ |  |  |  |  |  |  |  |  |
| 70 | Nelson et al. (2014) |  |  |  | ✔ |  |  |  |  |  |
| 71 | O’Brien et al. (2014) | ✔ |  |  |  |  |  |  |  |  |
| 72 | Obamiro et al. (2018) |  |  |  |  |  |  | ✔ |  |  |
| 73 | Paquette et al. (2017) | ✔ |  |  | ✔ |  |  |  |  | 2 |
| 74 | Paquette et al. (2018) | ✔ |  |  |  |  |  |  |  |  |
| 75 | Park et al. (2019) | ✔ |  |  |  |  |  |  |  |  |
| 76 | Pham and Brown (2019) |  |  | ✔ |  |  | ✔ |  |  | 2 |
| 77 | Renner et al. (2019) | ✔ |  |  |  |  |  |  |  |  |
| 78 | Shiga et al. (2015) | ✔ |  |  |  |  |  |  | ✔ | 2 |
| 79 | Shore et al. (2014) |  |  |  | ✔ |  | ✔ |  |  | 2 |
| 80 | Simons et al. (2016) |  | ✔ |  | ✔ |  |  |  |  | 2 |
| 81 | Song et al. (2017) |  |  |  | ✔ |  |  |  |  |  |
| 82 | Song et al. (2019) |  | ✔ |  |  |  |  |  |  |  |
| 83 | Sorensen et al. (2017) |  | ✔ |  | ✔ |  | ✔ |  |  | 3 |
| 84 | Stephenson et al. (2018) |  |  |  |  |  |  | ✔ |  |  |
| 85 | Tsai et al. (2013) |  |  |  |  |  | ✔ |  |  |  |
| 86 | Yao et al. (2016) |  |  |  | ✔ | ✔ | ✔ |  | ✔ | 4 |
| 87 | Zalesak et al. (2013) |  |  |  | ✔ |  |  |  |  |  |
| 88 | Zhou et al. (2015) |  |  |  | ✔ |  | ✔ |  |  | 2 |
|  | **Total (n=131)** | 12 | 18 | 5 | 48 | 5 | 30 | 7 | 6 |  |

a and b = two different studies with the same first author and year: Romoli (2021) included two studies by Baker et al. (2019); Coleman et al. (2016a) in Int J Cardiol, Coleman et al. (2016b) in PLoS One; Ozaki (2020) and Salmasi (2020) both included the same two studies by Deshpande et al. (2018); and Manzoor et al. (2017a) in J Thromb Thrombolysis, and Manzoor et al. (2017b) in Pharmacotherapy.

* Afzal (2019) only includes the seven of the 21 included studies that were relevant to adherence, persistence, discontinuation or switching

CCA = (131 - 88)/(88x8 - 88) = 7.0% = moderate

## Overlap of primary studies within reviews for stakeholder experiences

|  | **Review** | Buck (2021) | Jang (2021) | Katerenchuk (2021) | Afzal (2019) | Salmasi (2019) | Generalova (2018) | Clarkesmith (2017) | Loewen (2017) | Mas Dalmau (2017) | Pandya & Bajorek (2017) | Wilke (2017) | Willett & Morrill (2017) | Alamneh (2016) | Entezari-Maleki (2016) | Zhou (2016) | **Overlap** |
| --- | --- | --- | --- | --- | --- | --- | --- | --- | --- | --- | --- | --- | --- | --- | --- | --- | --- |
|  | **Included Studies** |  |  |  |  |  |  |  |  |  |  |  |  |  |  |  |  |
| 1 | Akao et al. (2014) |  |  |  |  |  |  |  |  |  |  |  |  | ✔ |  |  |  |
| 2 | Alegret et al. (2014) |  |  |  | ✔ |  |  |  |  |  |  |  |  |  |  |  |  |
| 3 | Alonso-Coello et al. (2014) |  |  |  |  |  |  |  | ✔ |  |  | ✔ |  |  |  |  | **2** |
| 4 | Altiok et al. (2015) |  |  |  |  | ✔ |  |  |  |  |  |  |  |  |  |  |  |
| 5 | Amara et al. (2016) |  |  |  |  | ✔ |  |  |  |  |  |  |  |  |  |  |  |
| 6 | Anderson et al. (2006) |  |  |  |  |  |  |  |  | ✔ |  |  |  |  |  |  |  |
| 7 | Andrade et al. (2016) |  |  |  |  |  | ✔ |  | ✔ |  |  | ✔ |  |  |  |  | **3** |
| 8 | Ansell et al. (2010) |  |  |  |  |  |  |  |  |  | ✔ |  |  |  |  |  |  |
| 9 | Arnsten et al. (1997) |  |  |  |  |  |  |  |  |  | ✔ |  |  |  |  |  |  |
| 10 | Attaya et al. (2012) |  |  |  |  |  |  |  | ✔ |  | ✔ | ✔ | ✔ |  |  |  | **4** |
| 11 | Bajorek et al. (2007) |  |  |  |  |  |  |  |  | ✔ | ✔ |  |  |  |  |  | **2** |
| 12 | Bajorek et al. (2009) |  |  |  |  |  |  |  |  |  | ✔ |  |  |  |  |  |  |
| 13 | Baker et al. (2009) |  |  |  |  |  |  |  |  |  |  |  |  | ✔ |  |  |  |
| 14 | Baker et al. (2011) |  |  |  |  | ✔ |  |  |  |  |  |  |  |  |  |  |  |
| 15 | Bamber et al. (2013) |  |  | ✔ | ✔ |  |  |  |  |  |  |  |  |  |  |  | **2** |
| 16 | Bannerjee et al. (2012) |  |  |  |  |  |  |  |  |  |  |  |  | ✔ |  |  |  |
| 17 | Barcellona et al. (2000) |  |  |  |  |  |  |  |  |  | ✔ |  |  |  |  |  |  |
| 18 | Barcellona et al. (2015) |  |  |  |  |  |  |  |  |  |  | ✔ |  |  |  |  |  |
| 19 | Barnes et al. (2014) |  |  |  |  |  |  |  |  |  |  |  |  | ✔ |  |  |  |
| 20 | Benzimra et al. (2018) |  |  | ✔ | ✔ |  |  |  |  |  |  |  |  |  |  |  | **2** |
| 21 | Bertozzo et al. (2016) | ✔ |  |  |  |  |  |  |  |  |  |  |  |  |  |  |  |
| 22 | Beyth et al. (2000) |  |  |  |  |  |  | ✔ |  |  |  |  |  |  |  |  |  |
| 23 | Biskupiak et al. (2014) |  |  |  |  |  |  |  |  |  |  |  |  | ✔ |  |  |  |
| 24 | Boom et al. (2015) |  |  |  |  |  |  |  |  |  |  | ✔ |  |  |  |  |  |
| 25 | Borg Xuereb et al. (2016) | ✔ |  |  |  |  |  |  |  |  |  |  |  |  |  |  |  |
| 26 | Bottger et al. (2015) |  |  |  |  |  |  |  | ✔ |  |  | ✔ |  |  |  |  | **2** |
| 27 | Brandes et al. (2013) |  |  |  |  |  |  |  |  |  |  |  |  | ✔ |  |  |  |
| 28 | Bungard et al. (2009) |  |  |  |  |  |  |  |  |  |  |  |  |  | ✔ |  |  |
| 29 | Bungard et al. (2012) |  |  |  |  |  |  |  |  |  |  |  |  |  | ✔ |  |  |
| 30 | Cabbar et al. (2019) |  |  | ✔ |  |  |  |  |  |  |  |  |  |  |  |  |  |
| 31 | Camm et al. (2010) |  |  |  |  |  |  |  |  |  |  |  |  | ✔ |  |  |  |
| 32 | Camm et al. (2016) |  |  |  |  |  |  |  |  |  |  |  | ✔ |  |  |  |  |
| 33 | Cano et al. (2018) |  |  | ✔ |  |  |  |  |  |  |  |  |  |  |  |  |  |
| 34 | Carrothers et al. (2014) |  |  |  | ✔ |  |  |  |  |  |  |  |  |  |  |  |  |
| 35 | Casais et al. (2005) |  |  |  |  |  |  |  |  |  | ✔ | ✔ |  |  |  |  | **2** |
| 36 | Castellucci et al. (2015) |  |  |  | ✔ |  |  |  |  |  |  |  |  |  |  |  |  |
| 37 | Chamberlain et al. (2001) |  |  |  |  |  |  |  |  |  |  |  |  |  | ✔ |  |  |
| 38 | Chan et al. (2006) |  |  |  |  |  |  |  |  |  |  |  |  |  |  | ✔ |  |
| 39 | Chan et al. (2011) |  |  |  |  |  |  |  |  |  |  |  |  | ✔ |  |  |  |
| 40 | Chenot et al. (2014) |  |  |  |  | ✔ |  |  |  |  |  |  |  |  |  |  |  |
| 41 | Choi et al. (2014) |  |  |  |  |  |  |  | ✔ |  | ✔ |  |  |  |  |  | **2** |
| 42 | Christensen et al. (2007) |  |  |  |  |  |  | ✔ |  |  |  |  |  |  |  |  |  |
| 43 | Clarkesmith et al. (2013) |  |  |  |  |  |  | ✔ |  |  |  |  |  |  |  |  |  |
| 44 | Coelho-Dantas et al. (2004) |  |  |  |  |  |  |  |  | ✔ |  |  |  |  |  |  |  |
| 45 | Coleman et al. (2004) |  |  |  |  |  |  |  |  |  |  |  |  |  | ✔ |  |  |
| 46 | Coleman et al. (2013) |  |  |  |  |  |  |  |  |  |  |  | ✔ |  |  |  |  |
| 47 | Coleman et al. (2016) |  |  | ✔ | ✔ |  |  |  |  |  |  |  |  |  |  |  | **2** |
| 48 | Contreras Muruaga et al. (2017) |  |  |  | ✔ |  |  |  |  |  |  |  |  |  |  |  |  |
| 49 | Cook-Campbell et al. (2010) |  |  |  |  | ✔ |  |  |  |  |  |  |  |  |  |  |  |
| 50 | Cottrell et al. (2009) |  |  |  |  |  |  |  |  |  |  | ✔ |  |  |  |  |  |
| 51 | Cutler and Everett (2010) |  |  |  |  |  |  |  |  |  |  |  |  | ✔ |  |  |  |
| 52 | Dantas et al. (2004) |  |  |  |  | ✔ |  |  |  |  | ✔ |  |  |  |  |  | **2** |
| 53 | Davis et al. (2005) |  |  |  |  |  |  |  |  |  | ✔ |  |  |  |  |  |  |
| 54 | De Caterina et al. (2013) |  |  |  |  |  |  |  |  |  |  |  |  | ✔ |  |  |  |
| 55 | De Caterina et al. (2018) |  |  | ✔ | ✔ |  |  |  |  |  |  |  |  |  |  |  | **2** |
| 56 | De Schryver et al. (2005) |  |  |  |  |  |  |  |  |  |  |  |  | ✔ |  |  |  |
| 57 | Decker et al. (2012) |  |  |  |  |  |  |  |  | ✔ |  |  |  |  |  |  |  |
| 58 | Deitelzweig et al. (2012) |  |  |  |  |  |  |  |  |  |  |  |  | ✔ |  |  |  |
| 59 | Deitelzweig et al. (2013) |  |  |  |  |  |  |  |  |  |  |  |  | ✔ |  |  |  |
| 60 | Deitelzweig et al. (2014) |  |  |  |  |  |  |  |  |  |  |  |  | ✔ |  |  |  |
| 61 | Desai et al. (2013) |  |  |  |  |  |  |  |  |  |  |  |  | ✔ |  |  |  |
| 62 | Desai et al. (2014) |  |  |  |  |  |  |  |  |  |  |  |  | ✔ |  |  |  |
| 63 | Desteghe et al. (2016) |  |  |  |  | ✔ |  |  |  |  |  |  |  |  |  |  |  |
| 64 | Devereaux et al. (2001) |  |  |  |  |  |  |  | ✔ |  |  | ✔ |  |  |  |  | **2** |
| 65 | DeWilde et al. (2006) |  |  |  |  |  |  |  |  |  |  |  |  | ✔ |  |  |  |
| 66 | Duran et al. (2012) |  |  |  |  |  |  |  |  |  |  |  |  | ✔ |  |  |  |
| 67 | Duran-Parrondo et al. (2011) |  |  |  |  |  |  |  |  |  |  |  |  |  | ✔ |  |  |
| 68 | Eikelboom et al. (2013) |  |  |  |  |  |  |  |  |  |  |  |  | ✔ |  |  |  |
| 69 | Elewa et al. (2014) |  |  |  |  |  |  |  | ✔ |  | ✔ |  | ✔ |  |  |  | **3** |
| 70 | Ernst et al. (2003) |  |  |  |  |  |  |  |  |  |  |  |  |  | ✔ |  |  |
| 71 | Fang et al. (2010) |  |  |  |  |  |  |  |  |  |  |  |  | ✔ |  |  |  |
| 72 | Faraoni et al. (2014) |  |  |  |  |  | ✔ |  |  |  |  |  |  |  |  |  |  |
| 73 | Fareau et al. (2015) |  |  | ✔ |  |  |  |  |  |  |  |  |  |  |  |  |  |
| 74 | Fatima et al. (2016) |  |  |  |  |  |  |  | ✔ |  |  |  |  |  |  |  |  |
| 75 | Fernandez et al. (2018) |  |  |  | ✔ |  |  |  |  |  |  |  |  |  |  |  |  |
| 76 | Frankel et al. (2015) |  |  |  |  | ✔ |  |  |  |  |  |  |  |  |  |  |  |
| 77 | Freeman et al. (2011) |  |  |  |  |  |  |  |  |  |  |  |  | ✔ |  |  |  |
| 78 | Fuller et al. (2004) |  |  |  |  |  |  |  |  | ✔ | ✔ |  |  |  |  |  | **2** |
| 79 | Gadisseur et al. (2003) |  |  |  |  |  |  | ✔ |  |  |  |  |  |  |  |  |  |
| 80 | Gage et al. (1996) |  |  |  |  |  |  |  | ✔ |  |  |  |  |  |  |  |  |
| 81 | Garton et al. (2011) |  |  |  |  |  |  |  |  |  |  |  |  |  | ✔ |  |  |
| 82 | Garwood et al. (2008) |  |  |  |  |  |  |  |  |  |  |  |  |  | ✔ |  |  |
| 83 | Gebler-Hughes et al. (2012) |  |  |  |  |  |  |  |  |  | ✔ |  |  |  |  |  |  |
| 84 | Gebler-Hughes et al. (2014) |  |  |  |  |  |  |  |  |  |  | ✔ |  |  |  |  |  |
| 85 | Geng et al. (2018) |  |  | ✔ |  |  |  |  |  |  |  |  |  |  |  |  |  |
| 86 | Ghijben et al. (2014) |  |  |  |  |  |  |  | ✔ |  |  | ✔ | ✔ |  |  |  | **3** |
| 87 | Gibbs et al. (2013) |  |  |  |  |  |  |  |  |  |  |  |  | ✔ |  |  |  |
| 88 | Goette et al. (2018) |  |  | ✔ |  |  |  |  |  |  |  |  |  |  |  |  |  |
| 89 | Gonzales-Rojas et al. (2012) |  |  |  |  |  |  |  |  |  |  | ✔ |  |  |  |  |  |
| 90 | Greinacher et al. (2015) |  |  |  |  |  |  |  |  |  |  |  |  | ✔ |  |  |  |
| 91 | Guhl et al. (2020) |  | ✔ |  |  |  |  |  |  |  |  |  |  |  |  |  |  |
| 92 | Gumbinger et al. (2015) | ✔ |  |  |  |  |  |  |  |  |  |  |  |  |  |  |  |
| 93 | Guo et al. (2017) |  | ✔ |  |  |  |  |  |  |  |  |  |  |  |  |  |  |
| 94 | Guo et al. (2020a) |  | ✔ |  |  |  |  |  |  |  |  |  |  |  |  |  |  |
| 95 | Guo et al. (2020b) |  | ✔ |  |  |  |  |  |  |  |  |  |  |  |  |  |  |
| 96 | Gupta et al. (2013) |  |  |  |  |  |  |  |  |  |  |  |  |  | ✔ |  |  |
| 97 | Haim et al. (2015) |  |  |  |  |  |  |  |  |  |  |  |  | ✔ |  |  |  |
| 98 | Hall et al. (2011) |  |  |  |  |  |  |  |  |  |  |  |  |  | ✔ |  |  |
| 99 | Hamilton et al. (2012) |  |  |  |  |  |  |  |  |  |  |  |  | ✔ |  |  |  |
| 100 | Hanon et al. (2016) |  |  | ✔ | ✔ |  |  |  |  |  |  |  |  |  |  |  | **2** |
| 101 | Hendriks et al. (2013) |  |  |  |  |  |  | ✔ |  |  |  |  |  |  |  |  |  |
| 102 | Ho et al. (2014) | ✔ |  |  |  |  |  |  |  |  |  |  |  |  |  |  |  |
| 103 | Hodden et al. (2000) |  |  |  |  |  |  |  |  |  |  |  |  |  | ✔ |  |  |
| 104 | Hohnloser et al. (2016) |  |  | ✔ | ✔ |  |  |  |  |  |  |  |  |  |  |  | **2** |
| 105 | Holbrook et al. (2007) |  |  |  |  |  |  |  | ✔ |  |  |  |  |  |  |  |  |
| 106 | Holbrook et al. (2013) |  |  |  |  |  |  |  | ✔ |  |  |  |  |  |  |  |  |
| 107 | Hong et al. (2013) |  |  |  |  |  |  |  | ✔ |  |  |  |  |  |  |  |  |
| 108 | Howitt and Armstrong (1999) |  |  |  |  |  |  |  | ✔ | ✔ |  |  |  |  |  |  | **2** |
| 109 | Howitt et al. (2000) |  |  |  |  |  |  |  |  |  | ✔ |  |  |  |  |  |  |
| 110 | Huang et al. (2013) |  |  |  |  |  | ✔ |  |  |  |  |  |  |  |  |  |  |
| 111 | Huisman et al. (2015) |  |  |  |  |  |  |  |  |  |  |  |  | ✔ |  |  |  |
| 112 | Jackson et al. (2004) |  |  |  |  |  |  |  |  |  |  |  |  |  | ✔ |  |  |
| 113 | Jackson et al. (2018) | ✔ |  |  |  |  |  |  |  |  |  |  |  |  |  |  |  |
| 114 | Kaariainen et al. (2013) |  |  |  |  |  |  |  |  |  | ✔ |  |  |  |  |  |  |
| 115 | Kakkar et al. (2011) |  |  |  |  |  |  |  |  |  |  |  |  | ✔ |  |  |  |
| 116 | Keita et al. (2017) |  |  | ✔ | ✔ |  |  |  |  |  |  |  |  |  |  |  | **2** |
| 117 | Kim et al. (2011) |  |  |  |  |  |  |  |  |  | ✔ |  |  |  |  |  |  |
| 118 | Kimmel et al. (2007) |  |  |  |  |  |  |  |  |  | ✔ |  |  | ✔ |  |  | **2** |
| 119 | Kirley et al. (2012) |  |  |  |  |  |  |  |  |  |  |  |  | ✔ |  |  |  |
| 120 | Kirley et al. (2016) |  |  |  |  |  | ✔ |  |  |  |  |  |  |  |  |  |  |
| 121 | Koponen et al. (2008) |  |  |  |  | ✔ |  |  |  |  |  |  |  |  |  |  |  |
| 122 | Koretsune et al. (2017) |  |  | ✔ | ✔ |  |  |  |  |  |  |  |  |  |  |  | **2** |
| 123 | Koretsune et al. (2018) |  |  | ✔ | ✔ |  |  |  |  |  |  |  |  |  |  |  | **2** |
| 124 | Labovitz et al. (2017) |  | ✔ |  |  |  |  |  |  |  |  |  |  |  |  |  |  |
| 125 | LaHay et al. (2014) |  |  |  |  |  |  |  | ✔ |  |  | ✔ |  |  |  |  | **2** |
| 126 | Lalonde et al. (2008) |  |  |  |  |  |  |  |  |  |  |  |  |  | ✔ | ✔ | **2** |
| 127 | Lane et al. (2006) |  |  |  |  | ✔ |  |  |  |  |  |  |  |  |  |  |  |
| 128 | Larochelle et al. (2018) |  |  | ✔ | ✔ |  |  |  |  |  |  |  |  |  |  |  | **2** |
| 129 | Larsen et al. (2015) |  |  |  |  |  | ✔ |  |  |  |  |  |  |  |  |  |  |
| 130 | Lancaster et al. (1991) |  |  |  |  |  |  |  |  | ✔ |  |  |  |  |  |  |  |
| 131 | Lee et al. (2013) |  |  |  |  | ✔ |  |  |  |  |  |  |  |  |  |  |  |
| 132 | Lee et al. (2016) |  | ✔ |  |  |  |  |  |  |  |  |  |  |  |  |  |  |
| 133 | Levitan et al. (2013) |  |  |  |  |  |  |  |  |  |  | ✔ |  |  |  |  |  |
| 134 | Lin et al. (2014) |  | ✔ |  |  |  |  |  |  |  |  |  |  |  |  |  |  |
| 135 | Lip et al. (2002) |  |  |  |  | ✔ |  |  |  |  | ✔ |  |  |  |  |  | **2** |
| 136 | Lip et al. (2006) |  |  |  |  |  |  |  |  |  | ✔ |  |  |  |  |  |  |
| 137 | Lip et al. (2007) |  |  |  |  | ✔ |  |  |  |  |  |  |  |  |  |  |  |
| 138 | Lip et al. (2013) |  |  |  |  |  | ✔ |  |  |  |  |  |  |  |  |  |  |
| 139 | Lip et al. (2014) |  |  |  |  |  |  |  |  |  |  |  |  | ✔ |  |  |  |
| 140 | Lip et al. (2015) |  |  |  |  |  |  |  |  |  |  |  |  | ✔ |  |  |  |
| 141 | Lipman et al. (2004) |  |  |  |  |  |  |  |  | ✔ |  |  |  |  |  |  |  |
| 142 | Man-Son-Hing et al. (1996) |  |  |  |  |  |  |  | ✔ |  |  | ✔ |  |  |  |  | **2** |
| 143 | Man-Son-Hing et al. (1999) |  |  |  |  |  |  | ✔ | ✔ |  |  |  |  |  |  |  | **2** |
| 144 | Man-Son-Hing et al. (2002) |  |  |  |  |  |  |  | ✔ |  |  |  |  |  |  |  |  |
| 145 | Marquez-Contreras et al. (2017) |  |  |  | ✔ |  |  |  |  |  |  |  |  |  |  |  |  |
| 146 | McAlister et al. (2005) |  |  |  |  |  |  | ✔ |  |  |  |  |  |  |  |  |  |
| 147 | McCabe et al. (2008) |  |  |  |  | ✔ |  |  |  |  |  |  |  |  |  |  |  |
| 148 | McCabe et al. (2011) |  |  |  |  | ✔ |  |  |  |  |  |  |  |  |  |  |  |
| 149 | McCabe et al. (2015) |  |  |  |  | ✔ |  |  |  |  |  |  |  |  |  |  |  |
| 150 | McCormick et al. (2001) |  |  |  |  |  |  |  |  |  |  |  |  | ✔ |  |  |  |
| 151 | Mega et al. (2012) |  |  |  |  |  |  |  |  |  |  |  |  | ✔ |  |  |  |
| 152 | Michel et al. (2013) |  |  |  |  |  |  |  |  |  | ✔ |  |  |  |  |  |  |
| 153 | Mohammed et al. (2013) |  |  |  |  |  |  |  |  |  |  |  |  | ✔ |  |  |  |
| 154 | Moia et al. (2013) |  |  |  |  |  |  |  |  |  | ✔ |  |  |  |  |  |  |
| 155 | Monz et al. (2013) |  |  |  | ✔ |  |  |  |  |  |  |  | ✔ |  |  |  | **2** |
| 156 | Motycka et al. (2012) |  |  |  |  |  |  |  |  |  |  |  |  |  | ✔ |  |  |
| 157 | Naganuma et al. (2017) | ✔ |  |  |  |  |  |  |  |  |  |  |  |  |  |  |  |
| 158 | Najafzadeh et al. (2014) |  |  |  |  |  |  |  |  |  |  | ✔ |  |  |  |  |  |
| 159 | Najafzadeh et al. (2015) |  |  |  |  |  |  |  |  |  |  | ✔ |  |  |  |  |  |
| 160 | Nedeloiu et al. (2011) |  |  |  |  | ✔ |  |  |  |  |  |  |  |  |  |  |  |
| 161 | Nelson et al. (2014) |  |  |  |  |  |  |  |  |  | ✔ |  |  |  |  |  |  |
| 162 | Nieuwlaat et al. (2006) |  |  |  |  |  |  |  |  |  |  |  |  | ✔ |  |  |  |
| 163 | Obamiro et al. (2018) |  |  |  | ✔ | ✔ |  |  |  |  |  |  |  |  |  |  | **2** |
| 164 | O’Brien et al. (2014) | ✔ |  |  |  |  |  |  |  |  |  |  |  |  |  |  |  |
| 165 | Ogilvie et al. (2010) |  |  |  |  |  |  |  |  |  |  |  |  | ✔ |  |  |  |
| 166 | Okumura et al. (2012) |  |  |  |  |  |  |  |  |  |  | ✔ |  |  |  |  |  |
| 167 | Okumura et al. (2015) |  |  |  |  |  |  |  | ✔ |  |  | ✔ |  |  |  |  | **2** |
| 168 | Okumura et al. (2018) |  |  | ✔ | ✔ |  |  |  |  |  |  |  |  |  |  |  | **2** |
| 169 | Olaiya et al. (2016) |  |  |  |  |  | ✔ |  |  |  |  |  |  |  |  |  |  |
| 170 | Oldgren et al. (2014) |  |  |  |  |  |  |  |  |  |  |  |  | ✔ |  |  |  |
| 171 | Orensky and Holdford (2005) |  |  |  |  |  |  |  |  |  | ✔ |  |  |  |  |  |  |
| 172 | Palacio et al. (2015) |  |  |  |  |  |  |  | ✔ |  |  | ✔ |  |  |  |  | **2** |
| 173 | Paquette et al. (2017) | ✔ |  |  |  |  |  |  |  |  |  |  |  |  |  |  |  |
| 174 | Paquette et al. (2018) | ✔ |  |  |  |  |  |  |  |  |  |  |  |  |  |  |  |
| 175 | Park et al. (2019) | ✔ |  |  |  |  |  |  |  |  |  |  |  |  |  |  |  |
| 176 | Parker et al. (2007) |  |  |  |  |  |  |  |  |  | ✔ |  |  |  |  |  |  |
| 177 | Patel-Naik et al. (2008) |  |  |  |  |  |  |  |  |  |  |  |  |  | ✔ |  |  |
| 178 | Piyaskulkaew et al. (2014) |  |  |  |  |  |  |  |  |  |  |  |  | ✔ |  |  |  |
| 179 | Platt et al. (2008) |  |  |  |  |  |  |  |  |  | ✔ |  |  |  |  |  |  |
| 180 | Poon et al. (2007) |  |  |  |  |  |  |  |  |  |  |  |  |  | ✔ |  |  |
| 181 | Potpara et al. (2014) |  |  |  |  |  | ✔ |  |  |  |  |  |  |  |  |  |  |
| 182 | Prins et al. (2015) |  |  | ✔ | ✔ |  |  |  |  |  |  |  |  |  |  |  | **2** |
| 183 | Prochaska et al. (2015) |  | ✔ |  |  |  |  |  |  |  |  |  |  |  |  |  |  |
| 184 | Prochaska et al. (2017) |  | ✔ |  |  |  |  |  |  |  |  |  |  |  |  |  |  |
| 185 | Protherhoe et al. (2000) |  |  |  |  |  |  |  | ✔ |  |  | ✔ |  |  |  |  | **2** |
| 186 | Prothero et al. (2001) |  |  |  |  |  |  |  |  |  |  | ✔ |  |  |  |  |  |
| 187 | Radley et al. (1995) |  |  |  |  |  |  |  |  |  |  |  |  |  | ✔ |  |  |
| 188 | Renner et al. (2019) | ✔ |  |  |  |  |  |  |  |  |  |  |  |  |  |  |  |
| 189 | Rewiuk et al. (2007) |  |  |  |  | ✔ |  |  |  |  |  |  |  |  |  |  |  |
| 190 | Reynolds et al. (2006) |  |  |  |  |  |  |  |  |  |  |  |  | ✔ |  |  |  |
| 191 | Robinson et al. (2001) |  |  |  |  |  |  |  | ✔ |  |  | ✔ |  |  |  |  | **2** |
| 192 | Rudd et al. (2010) |  |  |  |  |  |  |  |  |  |  |  |  |  | ✔ |  |  |
| 193 | Sauter et al. (2016) |  |  |  |  |  | ✔ |  |  |  |  |  |  |  |  |  |  |
| 194 | Shafrin et al. (2016) |  |  |  |  |  |  |  | ✔ |  |  |  |  |  |  |  |  |
| 195 | Shah and Gage (2011) |  |  |  |  |  |  |  |  |  |  |  |  | ✔ |  |  |  |
| 196 | Shah et al. (2014) |  |  |  |  |  |  |  |  |  |  |  |  | ✔ |  |  |  |
| 197 | Shiga et al. (2015) | ✔ |  |  |  |  |  |  |  |  |  |  |  |  |  |  |  |
| 198 | Shilbayeh et al. (2019) |  | ✔ |  |  |  |  |  |  |  |  |  |  |  |  |  |  |
| 199 | Smith et al. (2010) |  |  |  |  | ✔ |  |  |  |  |  |  |  |  |  |  |  |
| 200 | Sola et al. (2009) |  |  |  |  |  |  |  |  | ✔ |  |  |  |  |  |  |  |
| 201 | Sorea et al. (2014) |  |  |  |  |  |  |  |  |  |  |  |  | ✔ |  |  |  |
| 202 | Steinberg et al. (2013) |  |  |  |  |  |  |  |  |  |  |  |  | ✔ |  |  |  |
| 203 | Stephan et al. (2018) |  | ✔ |  |  |  |  |  |  |  |  |  |  |  |  |  |  |
| 204 | Stephenson et al. (2018) |  |  | ✔ | ✔ |  |  |  |  |  |  |  |  |  |  |  | **2** |
| 205 | St-Louis et al. (2003) |  |  |  |  | ✔ |  |  |  |  |  |  |  |  |  |  |  |
| 206 | Suarez et al. (2012) |  |  |  |  |  |  |  |  |  |  |  |  | ✔ |  |  |  |
| 207 | Suarez-Fernandez et al. (2018) |  |  | ✔ |  |  |  |  |  |  |  |  |  |  |  |  |  |
| 208 | Sudlow et al. (1998) |  |  |  |  |  |  |  | ✔ |  |  |  |  |  |  |  |  |
| 209 | Sudlow et al. (1999) |  |  |  |  |  |  |  |  |  |  | ✔ |  |  |  |  |  |
| 210 | Suryanarayan and Shulman (2014) |  |  |  |  |  |  |  |  |  |  |  |  | ✔ |  |  |  |
| 211 | Talboom-Kamp et al. (2017) |  | ✔ |  |  |  |  |  |  |  |  |  |  |  |  |  |  |
| 212 | Tan et al. (2012) |  |  |  |  |  |  |  |  |  | ✔ |  |  |  |  |  |  |
| 213 | Thomson et al. (2007) |  |  |  |  |  |  | ✔ |  |  |  |  |  |  |  |  |  |
| 214 | Thorne et al. (2014) |  |  |  |  |  |  |  |  |  | ✔ | ✔ |  |  |  |  | **2** |
| 215 | Vaughan Sarrazin et al. (2014) |  |  |  |  |  |  |  |  |  | ✔ |  |  |  |  |  |  |
| 216 | Verdino et al. (2015) |  |  |  |  |  |  |  |  |  |  |  |  | ✔ |  |  |  |
| 217 | Verret et al. (2012) |  |  |  |  |  |  |  |  |  |  |  |  |  |  | ✔ |  |
| 218 | Vormfelde et al. (2014) |  |  |  |  |  |  | ✔ |  |  |  |  |  |  |  |  |  |
| 219 | Waldo et al. (2005) |  |  |  |  |  |  |  |  |  |  |  |  | ✔ |  |  |  |
| 220 | Wang et al. (2013) |  |  |  |  |  |  |  |  |  |  | ✔ | ✔ |  |  |  | **2** |
| 221 | Wang et al. (2014) |  |  |  |  |  |  |  |  |  |  |  |  | ✔ |  |  |  |
| 222 | Wang et al. (2015) |  |  |  |  |  |  |  |  |  |  | ✔ |  |  |  |  |  |
| 223 | Waterman et al. (2004) |  |  |  |  |  |  |  |  |  | ✔ |  |  |  |  |  |  |
| 224 | Wild et al. (2004) |  |  |  |  |  |  |  |  | ✔ |  |  |  |  |  |  |  |
| 225 | Wild et al. (2009) |  |  |  |  |  |  |  | ✔ |  | ✔ |  |  |  |  |  | **2** |
| 226 | Wilke et al. (2018) |  |  | ✔ |  |  |  |  |  |  |  |  |  |  |  |  |  |
| 227 | Wilson et al. (2003) |  |  |  |  |  |  |  |  |  |  |  |  |  | ✔ |  |  |
| 228 | Wilson et al. (2004) |  |  |  |  |  |  |  |  |  |  |  |  |  | ✔ |  |  |
| 229 | Wilt et al. (1995) |  |  |  |  |  |  |  |  |  |  |  |  |  | ✔ |  |  |
| 230 | Witt et al. (2003) |  |  |  |  |  |  |  |  |  |  |  |  |  | ✔ |  |  |
| 231 | Witt et al. (2005) |  |  |  |  |  |  |  |  |  |  |  |  |  | ✔ |  |  |
| 232 | Witt et al. (2013) |  |  |  |  |  |  |  |  |  | ✔ |  |  |  |  |  |  |
| 233 | Wutzler et al. (2014) |  |  |  |  |  | ✔ |  |  |  |  |  |  |  |  |  |  |
| 234 | Xu et al. (2013) |  |  |  |  |  |  |  |  |  |  |  |  | ✔ |  |  |  |
| 235 | Young et al. (2011) |  |  |  |  |  |  |  |  |  |  |  |  |  | ✔ |  |  |
| 236 | Zamorano et al. (2012) |  |  |  |  |  |  |  |  |  | ✔ | ✔ |  |  |  |  | **2** |
| 237 | Zimetbaum et al. (2010) |  |  |  |  |  |  |  |  |  |  |  |  | ✔ |  |  |  |
|  | **Total (n= 284)** | **12** | **12** | **20** | **21** | **21** | **10** | **9** | **25** | **10** | **32** | **28** | **7** | **50** | **24** | **3** |  |

a and b = Jang (2021) included two studies by Guo et al. (2020); both referenced in the review paper

CCA = (284 - 237)/(237x15 - 237) = 1.4% = slight

**References:**

Pieper, D., Antoine, S. L., Mathes, T., Neugebauer, E. A., & Eikermann, M. (2014). Systematic review finds overlapping reviews were not mentioned in every other overview. *J Clin Epidemiol*, *67*(4), 368-375. <https://doi.org/10.1016/j.jclinepi.2013.11.007>

1. Not mixed AF/VTE population (population not reported) [↑](#footnote-ref-1)
