# Supplementary material for: Oral anticoagulants: a systematic overview of reviews on efficacy and safety, genotyping, self-monitoring, and stakeholder experiences
Source: Syst Rev. 2022 Oct 28;11:232. doi: 10.1186/s13643-022-02098-w (PMC9615370; doi:10.1186/s13643-022-02098-w)
Supplement: Supplementary file 9 — Additional file 9. Risk of bias assessment for reviews of stakeholder experiences [file 13643_2022_2098_MOESM9_ESM.docx]

Additional file 9. Risk of bias assessment for reviews of stakeholder experiences

| First author (year) | 1. Is the review question clearly and explicitly stated? | 2. Were the inclusion criteria appropriate for the review question? | 3. Was the search strategy appropriate? | 4. Were the sources and resources used to search for studies adequate? | 5. Were the criteria for appraising studies appropriate? | 6. Was critical appraisal conducted by two or more reviewers independently? | 7. Were there methods to minimise errors in data extraction? | 8. Were the methods used to combine studies appropriate? | 9. Was the likelihood of publication bias assessed? | 10. Were recommendations for policy and/or practice supported by the reported data? | 11. Were the specific directives for new research appropriate? |
| --- | --- | --- | --- | --- | --- | --- | --- | --- | --- | --- | --- |
| Afzal (2019) | + | + | + | + | + | - | + | + | - | + | + |
| Alamneh (2016) | + | + | + | + | - | - | - | - | - | + | + |
| Buck (2021) | + | + | + | + | + | + | + | + | - | + | + |
| Clarkesmith (2017) | + | + | + | + | + | + | + | + | - | + | + |
| Entezari-Maleki (2016) | + | + | + | + | + | + | - | + | - | + | + |
| Generalova (2018) | + | + | + | + | + | + | + | + | - | + | + |
| Jang (2021) | + | + | + | + | + | + | + | - | - | + | + |
| Katerenchuk (2021) | + | + | - | + | + | + | + | + | + | + | + |
| Loewen (2017) | + | + | + | + | + | + | + | + | - | + | + |
| Mas Dalmau (2017) | + | + | + | + | + | + | + | + | - | + | + |
| Pandya (2017) | + | + | + | + | - | - | - | + | - | + | + |
| Salmasi (2019) | + | + | + | + | + | + | - | + | - | + | + |
| Wilke (2017) | + | + | + | + | + | + | - | + | - | + | + |
| Willett (2017) | + | + | + | + | - | - | - | - | - | + | - |
| Zhou (2016) | + | + | + | + | + | + | - | + | + | + | + |

+ = yes, - = no

**Summary**

Fifteen reviews were included. The most common flaw was no assessment of publication bias (two reviews reported an assessment). Seven reviews did not report data checking or duplicate extraction. Four reviews did not report duplicate bias assessment, and three of these did not report a full assessment of bias. Three reviews did not use appropriate methods to combine studies. One review did not suggest appropriate areas for further research, and one did not report an appropriate search strategy. Alamneh, Pandya and Willett did not meet five, four and six criteria, respectively. Afzal, Entezari-Maleki, Jang, Salmasi, and Wilke did not meet two criteria, and the other seven reviews did not meet one criterion. Overall, most reviews were at a low risk of bias.
